# Global burden of acute lower respiratory infection associated with human parainfluenza virus in children younger than 5 years for 2018: a systematic review and meta-analysis

**DOI:** 10.1016/S2214-109X(21)00218-7

**Published:** 2021-06-21

**Authors:** Xin Wang, You Li, Maria Deloria-Knoll, Shabir A Madhi, Cheryl Cohen, Vina Lea Arguelles, Sudha Basnet, Quique Bassat, W Abdullah Brooks, Marcela Echavarria, Rodrigo A Fasce, Angela Gentile, Doli Goswami, Nusrat Homaira, Stephen R C Howie, Karen L Kotloff, Najwa Khuri-Bulos, Anand Krishnan, Marilla G Lucero, Socorro Lupisan, Maria Mathisen, Kenneth A McLean, Ainara Mira-Iglesias, Cinta Moraleda, Michiko Okamoto, Histoshi Oshitani, Katherine L O'Brien, Betty E Owor, Zeba A Rasmussen, Barbara A Rath, Vahid Salimi, Pongpun Sawatwong, J Anthony G Scott, Eric A F Simões, Viviana Sotomayor, Donald M Thea, Florette K Treurnicht, Lay-Myint Yoshida, Heather J Zar, Harry Campbell, Harish Nair

**Affiliations:** a Centre for Global Health, Usher Institute, Edinburgh Medical School, University of Edinburgh, Edinburgh, UK; b Department of International Health, International Vaccine Access Center, Johns Hopkins Bloomberg School of Public Health, Baltimore, MD, USA; c South African Medical Research Council, Vaccines and Infectious Diseases Analytical Research Unit, Soweto, South Africa; d Department of Science and Technology, National Research Foundation, Vaccine Preventable Diseases, Faculty of Health Sciences, University of the Witwatersrand, Johannesburg, South Africa; e Centre for Respiratory Disease and Meningitis, National Institute for Communicable Diseases, Johannesburg, South Africa; f School of Public Health, Faculty of Health Sciences, University of the Witwatersrand, Johannesburg, South Africa; g Research Institute for Tropical Medicine, Muntinlupa, Metro Manila, Philippines; h Department of Child Health, Tribhuvan University, Katmandu, Nepal; i the Centre for International Health, University of Bergen, Bergen, Norway; j Barcelona Global Health Institute, Hospital Clínic–University of Barcelona, Barcelona, Spain; k Centro de Investigação em Saúde de Manhiça, Maputo, Mozambique; l Institució Catalana de Recerca i Estudis Avançats, Barcelona, Spain; m Paediatric Infectious Diseases Unit, Pediatrics Department, Hospital Sant Joan de Déu, University of Barcelona, Barcelona, Spain; n Consorcio de Investigación Biomédica en Red de Epidemiología y Salud Pública, Madrid, Spain; o Clinical Virology Unit, Centro de Educación Médica e Investigaciones Clínicas, Argentina; p Public Health Institute of Chile, Región Metropolitana, Chile; q Ricardo Gutierrez Children Hospital, Buenos Aires, Argentina; r International Centre for Diarrhoeal Disease Research, Bangladesh, Dhaka, Bangladesh; s Discipline of Paediatrics, School of Women's and Children's Health, The University of New South Wales, Sydney, NSW, Australia; t Medical Research Council Unit, The Gambia at London School of Hygiene & Tropical Medicine, London, UK; u Department of Paediatrics, Child & Youth Health, University of Auckland, Auckland, New Zealand; v Department of Pediatrics and Department of Medicine, Center for Vaccine Development and Global Health, University of Maryland School of Medicine, Baltimore, MD, USA; w Department of Pediatrics, University of Jordan, School of Medicine, Amman, Jordan; x Centre for Community Medicine, All India Institute of Medical Sciences, New Delhi, India; y Research Institute for Tropical Medicine, Muntinlupa, Philippines; z Department of Medical Microbiology, Vestre Viken Hospital Trust, Drammen, Norway; aa Área de Investigación en Vacunas, Fundación para el Fomento de la Investigación Sanitaria y Biomédica de la Comunitat Valenciana, Salud Pública, Valencia, Spain; ab Barcelona Global Health Institute, Hospital Clínic–University of Barcelona, Barcelona, Spain; ac Infectious Pediatric Diseases Section, Hospital Universitario de Octubre, Universidad Complutense, Research Institute Hospital de Octubre, Madrid, Spain; ad Department of Virology, Tohoku University Graduate School of Medicine, Sendai, Japan; ae KEMRI-Wellcome Trust Research Programme, Kilifi, Kenya; af Fogarty International Center, National Institutes of Health, Bethesda, MD, USA; ag Vienna Vaccine Safety Initiative, Berlin, Germany; ah Université Bourgogne-Franche Comté, Besançon, France; ai Department of Virology, School of Public Health, Tehran University of Medical Sciences, Tehran, Iran; aj Division of Global Health Protection, Thailand Ministry of Public Health and US Centers for Disease Control and Prevention Collaboration, Nonthaburi, Thailand; ak KEMRI-Wellcome Trust Research Programme, Centre for Geographic Medicine Research, Kilifi, Kenya; al Nuffield Department of Tropical Medicine, Oxford University, Oxford, UK; am Department of Infectious Disease Epidemiology, London School of Hygiene & Tropical Medicine, London, UK; an Department of Pediatrics, Section of Infectious Diseases, University of Colorado, School of Medicine, Aurora, CO, USA; ao Department of Epidemiology and Center for Global Health, Colorado School of Public Health, Aurora, CO, USA; ap Epidemiology Department, Ministry of Health, Santiago, Chile; aq Department of Global Health and Development, Boston University School of Public Health, Boston, MA, USA; ar Department of Medical Virology, National Health Laboratory Service and School of Pathology, Faculty of Health Sciences, University of the Witwatersrand, Johannesburg, South Africa; as Department of Pediatric Infectious Diseases, Institute of Tropical Medicine, Nagasaki University, Nagasaki, Japan; at Department of Paediatrics & Child Health, Medical Research Council Unit on Child & Adolescent Health, University of Cape Town, Cape Town, South Africa

## Abstract

**Background:**

Human parainfluenza virus (hPIV) is a common virus in childhood acute lower respiratory infections (ALRI). However, no estimates have been made to quantify the global burden of hPIV in childhood ALRI. We aimed to estimate the global and regional hPIV-associated and hPIV-attributable ALRI incidence, hospital admissions, and mortality for children younger than 5 years and stratified by 0–5 months, 6–11 months, and 12–59 months of age.

**Methods:**

We did a systematic review of hPIV-associated ALRI burden studies published between Jan 1, 1995, and Dec 31, 2020, found in MEDLINE, Embase, Global Health, Cumulative Index to Nursing and Allied Health Literature, Web of Science, Global Health Library, three Chinese databases, and Google search, and also identified a further 41 high-quality unpublished studies through an international research network. We included studies reporting community incidence of ALRI with laboratory-confirmed hPIV; hospital admission rates of ALRI or ALRI with hypoxaemia in children with laboratory-confirmed hPIV; proportions of patients with ALRI admitted to hospital with laboratory-confirmed hPIV; or in-hospital case–fatality ratios (hCFRs) of ALRI with laboratory-confirmed hPIV. We used a modified Newcastle-Ottawa Scale to assess risk of bias. We analysed incidence, hospital admission rates, and hCFRs of hPIV-associated ALRI using a generalised linear mixed model. Adjustment was made to account for the non-detection of hPIV-4. We estimated hPIV-associated ALRI cases, hospital admissions, and in-hospital deaths using adjusted incidence, hospital admission rates, and hCFRs. We estimated the overall hPIV-associated ALRI mortality (both in-hospital and out-hospital mortality) on the basis of the number of in-hospital deaths and care-seeking for child pneumonia. We estimated hPIV-attributable ALRI burden by accounting for attributable fractions for hPIV in laboratory-confirmed hPIV cases and deaths. Sensitivity analyses were done to validate the estimates of overall hPIV-associated ALRI mortality and hPIV-attributable ALRI mortality. The systematic review protocol was registered on PROSPERO (CRD42019148570).

**Findings:**

203 studies were identified, including 162 hPIV-associated ALRI burden studies and a further 41 high-quality unpublished studies. Globally in 2018, an estimated 18·8 million (uncertainty range 12·8–28·9) ALRI cases, 725 000 (433 000–1 260 000) ALRI hospital admissions, and 34 400 (16 400–73 800) ALRI deaths were attributable to hPIVs among children younger than 5 years. The age-stratified and region-stratified analyses suggested that about 61% (35% for infants aged 0–5 months and 26% for 6–11 months) of the hospital admissions and 66% (42% for infants aged 0–5 months and 24% for 6–11 months) of the in-hospital deaths were in infants, and 70% of the in-hospital deaths were in low-income and lower-middle-income countries. Between 73% and 100% (varying by outcome) of the data had a low risk in study design; the proportion was 46–65% for the adjustment for health-care use, 59–77% for patient groups excluded, 54–93% for case definition, 42–93% for sampling strategy, and 67–77% for test methods. Heterogeneity in estimates was found between studies for each outcome.

**Interpretation:**

We report the first global burden estimates of hPIV-associated and hPIV-attributable ALRI in young children. Globally, approximately 13% of ALRI cases, 4–14% of ALRI hospital admissions, and 4% of childhood ALRI mortality were attributable to hPIV. These numbers indicate a potentially notable burden of hPIV in ALRI morbidity and mortality in young children. These estimates should encourage and inform investment to accelerate the development of targeted interventions.

**Funding:**

Bill & Melinda Gates Foundation.

**Research in context**

**Evidence before this study**

Human parainfluenza virus (hPIV), including four major serotypes, is commonly detected in childhood acute lower respiratory infections (ALRI). Results from multi-country pneumonia case-control studies show that hPIV is one of the leading causative viruses of childhood ALRI. Two meta-analyses estimated that hPIV could be detected in 2·7–5·8% of children younger than 5 years admitted to hospital for ALRI. The estimates were either developed on the basis of a small number of studies or for a specific country. No estimates have been made to quantify the global burden of ALRI because of hPIV among children younger than 5 years, or by narrow age bands. We searched PubMed for studies published between Jan 1, 1995, and May 25, 2021, which reported the global burden of hPIV, using the search terms “(parainfluenza OR PIV) AND (global) AND (child OR infant)”.

**Added value of this study**

We did a systematic review of 203 studies with data on hPIV-associated ALRI community incidence rates, hospital admission rates, proportion of laboratory-confirmed hPIV among ALRI cases admitted to hospital, and in-hospital case–fatality ratios, including 41 high-quality unpublished studies that provided data by narrow age bands among children younger than 5 years, and 45 studies from low-income and lower middle-income countries. We estimated that, in 2018, approximately 18·8 million (uncertainty range [UR] 12·8–28·9) ALRI cases, 725 000 (UR 433 000–1 260 000) ALRI hospital admissions, and 34 400 (UR 16 400–73 800) ALRI deaths were attributable to hPIV globally among children younger than 5 years. When analysed in the context of all-cause ALRI burden estimates, hPIV accounts for 13% of ALRI cases, 4–14% of ALRI hospital admissions, and 4% of ALRI mortality. Similar to other respiratory viruses, a larger proportion of hPIV hospital admissions (61%) and in-hospital deaths (66%) occurred in infants younger than 1 year.

**Implications of all the available evidence**

Our systematic review provides the first global burden estimates of hPIV-associated and hPIV-attributable ALRI among children younger than 5 years, and estimates by narrow age groups. These burden estimates show the role of hPIV in causing child ALRI morbidity and mortality, and should inform investment to accelerate the development of targeted interventions.

## Introduction

Acute lower respiratory infection (ALRI) is one of the leading causes of morbidity and mortality in children globally, accounting for 10% of mortality in children younger than 5 years in 2017.^1^ Human parainfluenza virus (hPIV), primarily from four major serotypes (hPIV-1 to hPIV-4), usually causes epidemics in the spring and early summer. hPIV can cause ALRI and more severe infections in young children, leading to a considerable disease burden.^2^, ^3^

Previous systematic reviews have estimated that hPIV could be detected in 2·7–5·8% of ALRI cases in children younger than 5 years, but the estimates were either developed on the basis of a small number of studies or for the population of only one country.^4^, ^5^ Results from pooled analyses have shown evidence for the causal attribution of hPIV, and estimated that 43–87% of ALRI cases with laboratory-confirmed hPIV in children younger than 5 years are attributable to hPIV.^6^, ^7^ There are no licensed hPIV vaccines or antiviral treatments.^8^ A few hPIV-3 candidate vaccines are under development, and have been assessed in phase 1 and 2 trials showing safety and immunogenicity in seronegative children older than 6 months.^9^, ^10^, ^11^ hPIV-1 and hPIV-2 candidate vaccines are also under development.^12^, ^13^, ^14^

Estimates have not been made to quantify the global burden of hPIV in children younger than 5 years. Thus, we estimated the global and regional number of hPIV-associated ALRI cases, hospital admissions, and deaths by age bands (ie, 0–5 months, 6–11 months, and 12–59 months) among children younger than 5 years for 2018. We also estimated the global burden of ALRI that is attributable to hPIV by accounting for the causal attribution of hPIV. These estimates could help to identify age groups that are at a high risk of severe hPIV respiratory infections, and guide health investment priorities, resource allocation, and development of targeted pharmaceutical and non-pharmaceutical intervention strategies.

## Methods

### Systematic review, definitions, and assessment of risk of bias

We did a systematic review of hPIV-associated ALRI burden in children younger than 5 years (appendix pp 5, 6). We searched MEDLINE, Embase, Global Health, Cumulative Index to Nursing and Allied Health Literature, Web of Science, Global Health Library, three Chinese databases (China National Knowledge Infrastructure, Wanfang, and Chongqing VIP), and Google search (for grey literature) for studies published between Jan 1, 1995, and Dec 31, 2020. We also contacted authors who might have relevant data, especially incidence, hospital admission rates, and in-hospital case–fatality ratios (hCFR) data. Several of these authors agreed to share more detailed and recent data (we included these in the research network). We used the terms and related words “parainfluenza virus” AND “acute lower respiratory infections” AND “burden” AND “children”. No language or publication restrictions were applied (languages other than English or Chinese were translated with Google translate), and three reviewers (XW, YL, and KAM) screened the titles and abstracts for eligibility. For potentially eligible studies, reviewers screened full-text articles for final inclusion, and extracted eligible data independently. Disagreements were resolved by discussion between reviewers. For datasets that overlapped in study population, location, and period, we included either the more detailed dataset (eg, stratified by finer age groups) or the more recent version. We supplemented the data from published studies with additional high-quality unpublished data (from ongoing studies or the re-analysis of previously published studies) using agreed standard approaches and definitions within the Respiratory Virus Global Epidemiology Network.^15^

We reported any of these data for children younger than 5 years: community incidence of ALRI (clinical pneumonia, according to the 2005 WHO Integrated Management of Childhood Illnesses)^16^ with laboratory-confirmed hPIV (ie, molecular test, culture, and antigen detection test); hospital admission rates of ALRI (a physician-confirmed diagnosis of ALRI) or ALRI with hypoxaemia in children with laboratory-confirmed hPIV; proportion of laboratory-confirmed hPIV among ALRI cases admitted to hospital; or hCFRs of ALRI with laboratory-confirmed hPIV. Details of case definitions are in the appendix (pp 3, 4).

Studies had to use a clear case definition for specimen collection and testing, and studies that reported incidence and hospital admission rate data had to show data for at least 1 complete calendar year (or at least one full season if in a temperate region with defined hPIV seasons). We included hCFR data for any length of study period. We included data on the proportion of hPIV positives if they were from at least 1 full calendar year. We excluded studies without a clear denominator population at risk (limited to those reporting incidence and hospital admission rate data), those in which hPIV was detected only in samples that were tested negative for other viruses, those which reported modelled burden estimates, those in which hPIV infections were diagnosed on the basis of serology alone, or those which only included population subgroups with high-risk conditions.

The risk of bias in included studies was assessed using a modified Newcastle-Ottawa Scale, containing seven domains: study design, adjustment for health-care use, patient groups excluded, case definition, sampling strategy, diagnostic testing, and hypoxaemia ascertainment (appendix pp 30, 31).^17^

### Statistical analysis

Our approach to burden estimation, including main analyses and sensitivity analyses, is summarised in the appendix (p 9). We estimated hPIV-associated ALRI cases, hospital admissions, and in-hospital deaths using a strategy similar to our previous analysis.^15^ We pooled incidence rates, hospital admission rates, and hCFRs of hPIV-associated ALRI using a generalised linear mixed model.^18^ Differences between studies were anticipated, thus we used the generalised linear mixed model that accounts for the differences. Before meta-analysis, for the incidence rate and hospital admission rate, we scaled the population-at-risk for the level of testing per study where available before meta-analyses (appendix p 28). Not all studies reported data for hPIV-4, so we adjusted the hPIV case number to account for missing hPIV-4 cases in incidence and hospital admission rates and proportion positives on the basis of an estimated proportion of hPIV-4 in all hPIV cases (12% based on 24 studies; appendix p 17). Similarly, we adjusted hCFR estimates to account for missing hPIV-4 hospital admissions and deaths on the basis of estimated proportion and hCFRs of four hPIV types (appendix p 18).

After meta-analyses, we chose the Monte Carlo Simulation to estimate morbidity burden because it allows us to combine meta-estimates and population estimates (UN population estimates for 2018).^19^ The median value of 10 000 samples simulated from a log–normal distribution was used as the point burden estimate and the 2·5th and 97·5th percentiles as the 95% uncertainty range (UR). In the main analysis, we reported estimates stratified by three non-overlapping age bands (0–5 months, 6–11 months, and 12–59 months) and by 2018 child mortality settings (low or high, with a cut-off point of the median value of the mortality rate of those younger than 5 years) for each outcome where available.^20^ The global results were calculated as the sum of age-specific and region-specific estimates. The numbers of cases were rounded to the nearest thousand and the number of deaths to the nearest hundred. In community settings, we reported the incidence for the overall age band (0–59 months) because data were insufficient to allow disaggregation by narrower age bands. We imputed the numbers of cases for 0–59 months using a multiple imputation approach if data were reported for other age bands (eg, 0–11 months; appendix p 28).^15^

We estimated the number of hPIV-associated in-hospital ALRI deaths by combining the estimates of hospital admissions and hCFRs of hPIV-associated ALRI.^21^ The adjusted hospital admissions and hCFRs (accounting for missing hPIV-4) were used in the analysis. Similar to morbidity estimation, the global estimates of mortality were calculated as the sum of the estimates by the three age groups and by child mortality settings.

We estimated the number of overall hPIV-associated ALRI deaths by combining the estimates of hPIV-associated in-hospital deaths and the ratio of overall deaths to in-hospital deaths (labelled as an inflation factor). Details of data sources and analyses of the inflation factor have been described previously (appendix pp 19, 20).^15^ Briefly, the median ratio of overall ALRI deaths to in-hospital ALRI deaths at eight sites in six countries with high child mortality was extrapolated to other countries with high child mortality. For settings with low child mortality, the reciprocal of the proportion of children with pneumonia symptoms who received care, measured in Multiple Indicator Cluster Surveys, Demographic and Health Surveys, and other national surveys, was used as a proxy for inflation factor.^22^ The median value was extrapolated to other countries with low child mortality without data.

On the basis of hPIV-associated ALRI burden estimates, we estimated the ALRI burden attributable to hPIV by accounting for the attributable fraction of hPIV in hPIV-associated ALRI cases and deaths. Two pooled analyses of multi-country data showed that the attributable fraction of hPIV-associated ALRI cases varied by type;^6^, ^7^ we estimated the average attributable fraction of hPIV cases and hospital admissions using type-specific attributable fraction estimates and the pooled proportion of four hPIV-type cases (appendix p 22). The attributable fraction of hPIV-associated ALRI deaths was calculated using the attributable fraction of hPIV-associated ALRI cases and the ratio between the hCFRs of hPIV-positive ALRI and hPIV-negative ALRI. We assumed that the hCFR of hPIV-negative ALRI was equal to the hCFR of hPIV-positive ALRI that were not deemed attributable to hPIV (appendix pp 23, 24).

### Sensitivity analyses

For hPIV-associated ALRI morbidity and in-hospital deaths, we reported estimates by country development regions according to UNICEF definitions and by World Bank income levels (low-income and lower-middle-income, upper-middle-income, and high-income) in sensitivity analyses.^23^, ^24^ Additionally, we estimated the range of hPIV-associated ALRI hospital admissions by applying the proportion of those positive for hPIV in ALRI hospital admissions and the estimates of all-cause ALRI hospital admissions among children younger 5 years for 2015–16 (appendix p 13).^25^, ^26^ We estimated the overall hPIV-associated ALRI mortality for settings with high child mortality in a sensitivity analysis by applying the proportion of ALRI deaths positive for hPIV to the number of ALRI deaths among children younger than 5 years for 2017.^1^ The proportion of ALRI deaths positive for hPIV was estimated using data from 12 hospital-based studies retrieved with our systematic review (including five PERCH sites) from high mortality burden settings in which at least 90% of ALRI cases were tested and at least five ALRI deaths were identified during the study period (appendix p 21). In the sensitivity analysis, the hPIV-attributable ALRI mortality for settings with high child mortality was estimated using another approach by applying the proportion of hPIV-attributable ALRI deaths to the number of ALRI deaths among children younger than 5 years. The proportion of hPIV-attributable ALRI deaths was estimated using data in the period from December, 2016, to October, 2019, from the Child Health and Mortality Prevention Surveillance (also known as CHAMPS; appendix p 25).^27^

We report the estimates from the main analysis. Sensitivity results are in the appendix (pp 10–16, 18, 21, 25), as are details of the included studies (pp 39–58). All analyses were done in R (version 3.6.1).^28^, ^29^ This study was done and reported in accordance with the Guidelines for Accurate and Transparent Health Estimates Reporting recommendations (appendix p 72).^30^ The systematic review protocol was registered on PROSPERO (CRD42019148570).

### Role of the funding source

The funder of the study had no role in study design, data collection, data analysis, data interpretation, or writing of the report.

## Results

We identified 203 studies with data on hPIV-associated ALRI community incidence rates (13 studies), hospital admission rates (38 studies), proportion of those positive for hPIV in ALRI hospital admissions (168 studies), and hCFRs (58 studies; figure). There were 41 unpublished studies from the collaborative network and 162 studies from published literature. By World Bank income levels, eight studies were from low-income countries, 37 from lower-middle-income countries, 109 from upper-middle-income countries, and 49 from high-income countries. The number of studies is summarised in the appendix (pp 7–8).

**Figure:**
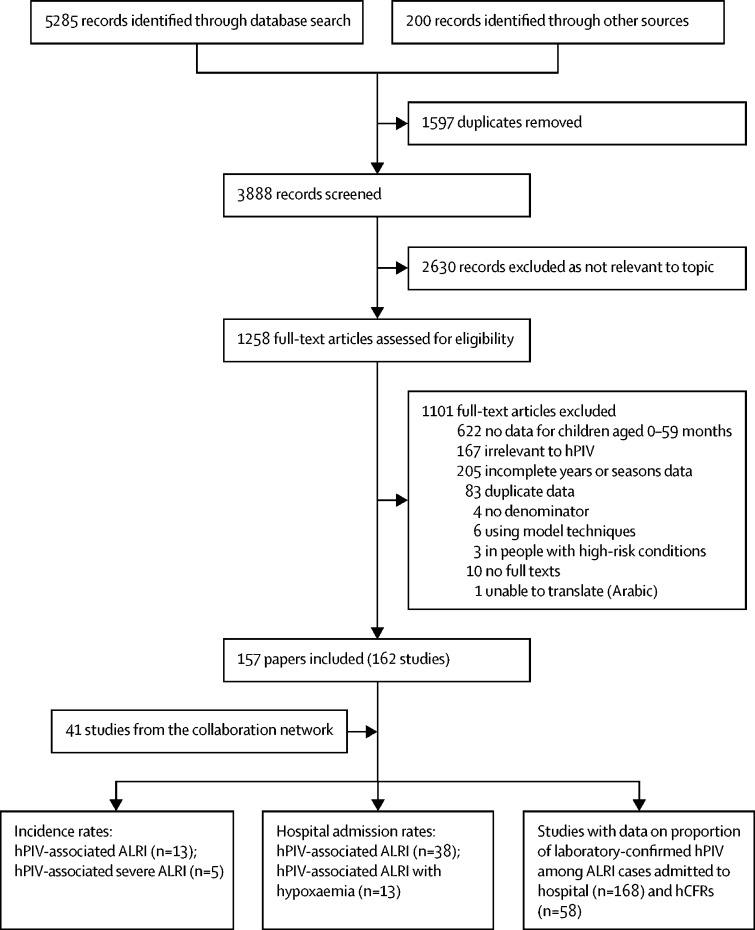
Flow diagram for study selection for hPIV-associated ALRI burden A study was defined as a dataset from one site in one published paper or from one research group in the network. Some studies provided more than one type of data, so the sum of studies across types was larger than the total number of included studies. ALRI=acute lower respiratory infection. hCFR=in-hospital case–fatality ratio. hPIV=human parainfluenza virus.

We identified 13 studies with incidence rates of hPIV-associated ALRI. There were 12 studies with data for 0–59 months (including imputed data). Seven studies reported the rates for before 2010. One study detected all four hPIV types, one study only detected hPIV-3, and other studies detected hPIV-1 to hPIV-3. The adjusted hPIV-associated ALRI incidence rate meta-estimate was 38·8 (95% CI 30·1–50·2) per 1000 children per year for ages 0–59 months for settings with high child mortality, and 37·8 (18·5–77·3) per 1000 children per year for settings with low child mortality. The high incidence in low child mortality settings was mainly driven by two Australian studies (one for 1996–99, and the other for 2010–14). We estimated 26·1 million (UR 17·8–40·1) hPIV-associated ALRI cases globally in children younger than 5 years (table 1).

**Table 1:** Estimates of the incidence (per 1000 children per year), and number of hPIV-associated ALRI cases in age groups of children younger than 5 years in 2018, by World Bank income level and child mortality setting

	**Low-income and lower-middle-income countries**	**Upper-middle-income countries**	**High-income countries**	**Low child mortality settings**	**High child mortality settings**	**Global estimates (low and high child mortality settings)**
**0–5 months**						
Number of studies	5	1	0	0	6	..
Incidence (95% CI)*	43·2 (11·7–146·4)	..	..	..	51·1 (18·1–136·0)	..
ALRI cases (thousands; UR)	1913 (545–6723)	..	..	..	2352 (863–6415)	..
**6–11 months**						
Number of studies	5	1	0	0	6	..
Incidence (95% CI)*	89·3 (65·7–120·2)	..	..	..	85·7 (66·0–110·6)	..
ALRI cases (thousands; UR)	3920 (2903–5294)	..	..	..	3911 (3025–5056)	..
**12–59 months**						
Number of studies	4	1	0	0	5	..
Incidence (95% CI)*	28·5 (15·9–50·6)	..	..	..	28·5 (17·9–45·2)	..
ALRI cases (thousands; UR)	9774 (5497–17386)	..	..	..	10 164 (6413–16 115)	..
**0–59 months**						
Number of studies†	7 (3)	1	4 (3)	4 (3)	8 (3)	..
Incidence (95% CI)*	37·7 (27·8–51·0)	..	37·8 (18·5–77·3)	37·8 (18·5–77·3)	38·8 (30·1–50·2)	..
ALRI cases (thousands; UR)	16 247 (12 013–21 978)	..	2395 (1176–4880)	8670 (4258–17 664)	17 417 (13 504–22 467)	26 087 (17 762–40 131)

The median value of the 2018 younger than 5 years mortality rate was used as the cutoff point for high child mortality and low child mortality settings. The incidence rate was adjusted to account for the missing hPIV-4 (ten studies). In one study, only the hPIV-3 rate was available; the rate was adjusted to account for the missing hPIV-1, hPIV-2, and hPIV-4. For the remaining one study, four types were detected and the rate was not adjusted. ALRI=acute lower respiratory infections. hPIV=human parainfluenza virus. UR=uncertainty range.

* Incidence (per 1000 children per year) from meta-analyses.

† The number in the parentheses shows the number of imputed studies.

There were 38 studies with hPIV-associated ALRI hospital admission rates, including 26 studies reporting data by three narrow age bands: 0–5 months, 6–11 months, or 12–59 months. Only nine studies reported data for all four hPIV types. The adjusted hospital admission rate was higher in infants (0–11 months) than children aged 12–59 months across World Bank income levels and child mortality settings. Particularly in settings with high child mortality, the rate was 5·8 (95% CI 3·7–9·2) per 1000 children per year for ages 0–5 months, 4·7 (3·2–6·7) for 6–11 months, and 0·7 (0·5–1·2) for children aged 12–59 months (table 2). In the main analysis (by child mortality settings), we estimated 1 007 000 (UR 601 000–1 750 000) hPIV-associated ALRI hospital admissions globally in children younger than 5 years. Of these hospital admissions, 35% were in children aged 0–5 months, 26% were in children aged 6–11 months, and 39% were in children aged 12–59 months.

**Table 2:** Hospital admission rates (per 1000 children per year), and hospital admissions of hPIV-associated ALRI in age groups of children younger than 5 years in 2018, by World Bank income level and child mortality setting

	**Low-income and lower-middle-income countries**	**Upper-middle-income countries**	**High-income countries**	**Low child mortality settings**	**High child mortality settings**	**Global estimates (low and high child mortality settings)**
**hPIV-associated ALRI 0–5 months**						
Number of studies	7	6	4	7	10	..
Hospital admission rate (95% CI)	3·8 (1·8–7·8)	5·7 (3·0–10·5)	5·5 (3·1–9·9)	3·6 (1·8–7·0)	5·8 (3·7–9·2)	..
Hospital admissions (thousands; UR)	168 (81–349)	105 (56–196)	35 (20–62)	83 (42–163)	267 (170–420)	350 (212–583)
**hPIV-associated ALRI 6–11 months**						
Number of studies	7	5	3	5	10	..
Hospital admission rate (95% CI)	3·5 (1·7–7·0)	3·8 (1·9–7·6)	3·5 (1·9–6·5)	2·0 (0·9–4·6)	4·7 (3·2–6·7)	..
Hospital admissions (thousands; UR)	154 (76–311)	70 (35–139)	22 (12–41)	46 (20–104)	214 (149–310)	260 (169–413)
**hPIV-associated ALRI 12–59 months**						
Number of studies	8	8	4	8	12	..
Hospital admission rate (95% CI)	0·8 (0·4–1·4)	0·8 (0·4–1·6)	0·8 (0·2–2·9)	0·8 (0·3–1·9)	0·7 (0·5–1·2)	..
Hospital admissions (thousands; UR)	274 (147–512)	117 (59–233)	41 (11–153)	147 (59–368)	250 (162–386)	396 (220–753)
**hPIV-associated ALRI 0–59 months**						
Hospital admissions (thousands; UR)	596 (304–1171)	292 (150–569)	98 (42–257)	276 (121–634)	731 (480–1116)	1007 (601–1750)
**hPIV-associated ALRI with hypoxaemia 0–5 months**						
Number of studies	6	3	1	2	7	..
Hospital admission rate (95% CI)	0·6 (0·3–1·6)	1·8 (1·1–3·0)	..	0·2 (0·1–0·3)	1·4 (0·8–2·4)	..
Hospital admissions (thousands; UR)	27 (12–61)	33 (20–55)	..	5 (3–8)	64 (37–111)	69 (40–119)
**hPIV-associated ALRI with hypoxaemia 6–11 months**						
Number of studies	6	3	1	2	7	..
Hospital admission rate (95% CI)	0·3 (0·1–0·9)	1·0 (0·5–1·8)	..	0·1 (0·0–0·2)	0·8 (0·5–1·4)	..
Hospital admissions (thousands; UR)	13 (4–39)	18 (10–35)	..	2 (0–14)	36 (22–61)	39 (22–75)
**hPIV-associated ALRI with hypoxaemia 12–59 months**						
Number of studies	6	6	1	4	8	..
Hospital admission rate (95% CI)	0·1 (0·1–0·2)	0·1 (0·0–1·2)	..	0·1 (0·0–4·4)	0·1 (0·1–0·2)	..
Hospital admissions (thousands; UR)	34 (24–48)	15 (1–223)	..	18 (1–534)	36 (25–50)	54 (26–584)
**hPIV-associated ALRI with hypoxaemia 0–59 months**						
Hospital admissions (thousands; UR)	74 (40–149)	66 (31–313)	..	25 (4–556)	137 (84–223)	162 (88–779)

The median value of the 2018 younger than 5 years mortality rate was used as the cutoff point for settings with high child mortality and low child mortality. The hospital admission rate was adjusted to account for the missing hPIV-4. Hospital admission rates from meta-analyses. ALRI=acute lower respiratory infections. hPIV=human parainfluenza virus. UR=uncertainty range.

There were 13 studies reporting hospital admission rates for hPIV-associated ALRI with hypoxaemia by age band, including nine studies reporting data for all four hPIV types. In the analysis stratified by child mortality settings, we estimated 162 000 (UR 88 000–779 000) hospital admissions for hPIV-associated ALRI with hypoxaemia (adjusted for missing hPIV-4) in children aged 0–59 months globally, accounting for 16% of the hPIV-associated ALRI hospital admissions.

We identified 58 studies reporting the hCFRs of hPIV-associated ALRI in children younger than 5 years, including 27 studies with data stratified by three narrow age bands. Eight studies did not report data for hPIV-4. The hCFR meta-estimates did not vary much by age band; however, we observed a 1–2-times difference across settings. Children in countries with high child mortality and in lower-middle-income countries generally had the highest hCFRs (2·3–3·6% for high child mortality settings; 2·0–3·9% for lower-middle-income countries; table 3). We estimated 25 700 (UR 12 000–56 500) hPIV-associated in-hospital ALRI deaths in children younger than 5 years. Of these deaths, approximately 42% were in children aged 0–5 months, 24% were in children aged 6–11 months, and 34% were in children aged 12–59 months (table 3).

**Table 3:** hCFR meta-estimates of hPIV-associated ALRI and in-hospital deaths in age groups of children younger than 5 years in 2018, by World Bank income level and child mortality setting

		**Low-income and lower-middle-income countries**	**Upper-middle-income countries**	**High-income countries**	**Low child mortality settings**	**High child mortality settings**	**Global estimates (low and high child mortality settings)**
Number of studies		15	8	4	7	20	..
0–5 months							
	hCFR, % (95% CI)	3·9 (2·1–7·3)	2·4 (1·3–4·6)	0·9 (0·2–3·6)	1·3 (0·6–3·1)	3·6 (2·2–5·8)	..
	Deaths (UR)	6600 (2600–17 000)	2500 (1100–6100)	300 (100–1500)	1100 (400–3100)	9600 (5000–18 600)	10 700 (5400–2 1700)
6–11 months							
	hCFR, % (95% CI)	2·0 (0·5–7·4)	3·8 (2·2–6·6)	1·2 (0·3–4·7)	2·6 (1·0–6·9)	2·3 (0·9–5·8)	..
	Deaths (UR)	3100 (700–13 800)	2700 (1100–6400)	300 (100–1200)	1200 (300–4200)	4900 (1800–13 300)	6100 (2200–17300)
12–59 months							
	hCFR, % (95% CI)	3·5 (2·2–5·6)	1·9 (0·8–4·1)	0·9 (0·4–1·9)	1·2 (0·7–2·3)	2·8 (1·8–4·4)	..
	Deaths (UR)	9600 (4500–20 900)	2200 (800–6400)	400 (100–1700)	1800 (600–5200)	7000 (3800–13 000)	8800 (4400–18 100)
0–59 months							
	Deaths (UR)	19 400 (7800–50 800)	7400 (3000–18 900)	1000 (200–4100)	4100 (1400–12 400)	21 600 (10 600–44 100)	25 700 (12 000–56 500)

The median value of the 2018 younger than 5 years mortality rate was used as the cutoff point for settings with high child mortality and low child mortality. The in-hospital deaths were estimated using adjusted hospital admissions and adjusted hCFRs to account for the missing hPIV-4. hCFR estimates were from meta-analyses. ALRI=acute lower respiratory infections. hCFR=In-hospital case-fatality ratio. hPIV=human parainfluenza virus. UR=uncertainty range.

We estimated a median inflation factor of 2·2 for settings with high child mortality and 1·3 for those with low child mortality (appendix p 20). Combining the estimates of inflation factors and hPIV-associated in-hospital ALRI mortality, we estimated 47 600 (UR 23 400–97 100) overall hPIV-associated ALRI deaths in high child mortality settings, and 53 000 (25 300–113 500) deaths globally.

We estimated an average attributable fraction of 72% for hPIV-associated ALRI cases, and 65% for hPIV-associated ALRI deaths among children younger than 5 years (table 4; appendix pp 20–22). Thus, we estimated that 18·8 million (UR 12·8–28·9) ALRI cases, 725 000 (433 000–1 260 000) ALRI hospital admissions, and 34 400 (16 400–73 800) ALRI deaths could be attributed to hPIV in children younger than 5 years globally. There were 30 900 (UR 15 200–63 100) hPIV-attributable ALRI deaths in settings with high child mortality.

**Table 4:** Estimates of global number of hPIV-attributable ALRI cases, hospital admissions, and deaths among children younger than 5 years in 2018 using attributable fraction of hPIV-associated ALRI

	Attributable fraction (%)*	**Global hPIV–associated burden estimates (UR)**	**Global hPIV–attributable burden estimates**†**(UR)**
ALRI cases (millions)	72%‡	26·1 (17·8–40·1)	18·8 (12·8–28·9)
ALRI hospital admissions (thousands)	72%	1007 (601–1750)	725 (433–1260)
ALRI deaths	65%§	53 000 (25 300–113 500)	34 400 (16 400–73 800)

ALRI=acute lower respiratory infections. hPIV=human parainfluenza virus. UR=uncertainty range.

* The fraction of ALRI cases and deaths with laboratory-confirmed hPIV that are attributable to hPIV.

† Applying the corresponding attributable fraction to the estimates of hPIV–associated burden.

‡ The attributable fraction for hPIV–associated ALRI cases was calculated using type-specific attributable fraction and prevalence. Details and the references are in the appendix (p 19).

§ The attributable fraction for hPIV-associated ALRI deaths was modelled using the attributable fraction for hPIV cases and the ratio of case-fatality between hPIV-attributable cases and hPIV-associated cases. Details are in the appendix (pp 20,21).

We did several sensitivity analyses to estimate hospital admissions, in-hospital deaths, and overall deaths of hPIV-associated ALRI, as well as hPIV-attributable ALRI deaths. For global hPIV-associated ALRI hospital admissions, the estimates in children younger than 5 years ranged from 986 000 to 1 007 000 in analyses by different stratification groups (appendix pp 11, 12); the proportion-based approach yielded a broader range (ie, from 452 000 to 1 443 000 admissions; appendix p 13). The point estimate of global in-hospital deaths ranged from 25 700 to 27 800 in children younger than 5 years, and an estimated 70% of the global in-hospital deaths occurred in lower-middle-income countries (19 400 of 27 800) based on the results by World Bank income level (appendix pp 14, 15). We estimated 56 100 (UR 36 500–87 400) hPIV-associated ALRI deaths and 45 500 (24 900–91 700) hPIV-attributable ALRI deaths in children younger than 5 years in settings with high child mortality in sensitivity analyses (appendix pp 21, 25).

Between 73% and 100% (varying by outcome) of the studies had a low risk in study design, 46–65% of the studies had a low risk in adjustment for health-care use, 59–77% of studies had a low risk in patient groups excluded, 54–93% of studies had a low risk in case definition, 42–93% of studies had a low risk in sampling strategy, and 67–77% of studies had a low risk in test methods (appendix p 8). Differences in estimates were found for all outcomes (appendix pp 43–44). For example, the unadjusted incidence rate of hPIV-associated ALRI ranged from 17·3 (95% CI 9·3–31·8) to 56·5 (38·1–82·9) per 1000 children per year for ages 0–59 months between studies. Hospital admission rates of hPIV-associated ALRI ranged from 0·8 (0·1–5·4) to 30·1 (18·2–49·3) per 1000 children per year for 0–5 months, 0·6 (0·4–0·8) to 18·1 (9·8–33·4) for 6–11 months, and 0·0 (0·0-1000·0) to 6·8 (5·3–8·7) for 12–59 months between studies.

## Discussion

We report the first global hPIV-associated and hPIV-attributable ALRI burden estimates among children younger than 5 years. A comparison between available all-cause ALRI burden estimates and our hPIV-specific estimates suggests that hPIV could be detected in 19% of ALRI cases (26 of 138 million), 6–20% of ALRI hospital admissions (1 of 5–16 million), and 7% of ALRI mortality (53 000 of 809 000) among children younger than 5 years globally.^1^, ^25^, ^26^ Of note, the number of hPIV-associated ALRI cases was developed on the basis of few data, and therefore needs to be verified with additional data. hPIV can be detected in the upper respiratory tract in healthy children and mere isolation does not indicate a causal association for ALRI;^7^ the use of attributable fraction therefore allowed us to estimate the proportion of ALRI cases in which hPIV is isolated and is the probable cause. Estimates from this analysis suggested that 13% of ALRI cases (18·8 of 138·0 million), 4–14% ALRI hospital admissions (0·7 of 5·0–16·0 million), and 4% of ALRI mortality (34 400 of 809 000) could be attributed to hPIV. When considered in the context of our other virus-specific ALRI burden estimates, hPIV-attributable ALRI hospital admissions and mortality in children younger than 5 years seem higher than for human metapneumovirus (approximately 500 000 human metapneumovirus-attributable ALRI hospital admissions and 11 000 deaths), but similar to that for seasonal influenza virus (approximately 700 000 influenza virus-attributable ALRI hospital admissions), and lower than the burden for respiratory syncytial virus.^15^, ^21^, ^31^ These estimates indicate that hPIV is an important virus, in that it is a substantial burden, related to ALRI in young children.

Similar to other viruses, a large proportion of the hPIV-associated ALRI burden occurred in infants younger than 1 year (61% of hPIV hospital admissions and 66% of hPIV in-hospital deaths), and children in low-income and lower-middle-income countries had the highest hCFRs.^15^, ^21^, ^26^ The high burden in infants might reflect their immature immune systems and decay of maternal antibodies.^32^, ^33^ Children aged 12–59 months still had high hCFRs compared with infants, which might reflect the virulence of hPIV and have implications for development of prevention strategies. Differences in data availability exist for different viruses, and the current hPIV burden estimates are based on fewer datapoints than those for seasonal influenza virus and respiratory syncytial virus, and will probably be refined when more data are available in future.

Our global burden estimates were generally robust in sensitivity analyses. The estimates for global hPIV-associated ALRI hospital admissions and in-hospital mortality, and overall hPIV-associated ALRI mortality, were all similar in different stratification groups and other sensitivity analyses (appendix pp 11–15, 21, 25). We reported a more conservative estimate of hPIV-attributable mortality in our main analysis (using the attributable fraction approach), and the point estimate increased by 47% (30 900/45 500) in settings with high child mortality in a sensitivity analysis (appendix p 25). We calculated the attributable fraction of hPIV in hPIV-confirmed cases and deaths using data from two pooled analyses, which were mainly from settings with high child mortality.^6^, ^7^ The estimates might have limited the representativeness of these data for low child mortality settings. The attributable fraction approach, estimated using data from case-control studies, indicates the probable true burden of hPIV in ALRI. However, making a causal inference is a challenge when using data from observational studies.

In general, we used the same analytical strategy as used previously,^15^, ^21^ with one adaptation in the current analysis to enable us to report burden estimates for the four hPIV types based on studies that reported data either for three types (hPIV-1 to hPIV-3) or four types. Our four hPIV-type ALRI burden estimates were adjusted on the basis of two key variables. One variable was the proportion of hPIV-4 in all hPIVs (12%), which was estimated in a pooled analysis of 24 hospital-based studies. We extrapolated this prevalence to community settings. There was only one community-based study detecting all four types and reporting hPIV-4 in 20% of hPIV cases. We consider the extrapolation to improve the generalisation of this adjustment, although it might have led to a conservative estimate of hPIV-4 associated ALRI cases. The second set of variables, hCFRs for four hPIV types, was estimated using data from five studies from countries with high child mortality. The type-specific hCFRs had wide and overlapping confidence intervals, especially for hPIV-2 and hPIV-4, reflecting the substantial variation across studies and limited precision because of the small case numbers for the two types. Additional data on hPIV type-specific hCFRs might help to refine the analyses and estimates.

The wide URs of the burden estimates reflected the differences in hPIV epidemiology between populations, methodological differences, and paucity of data. As shown in the described risk of bias of studies, methodological heterogeneity between studies could have probably biased our estimates (appendix p 8). hPIV-associated ALRI hospital admissions could have been overestimated in 27% (seven of 26 studies; appendix p 8) of hospital admission studies, which adopted broader case definitions (eg, patients with ALRI admitted to hospital; patients with ALRI or croup admitted to hospital). Underdetection (a detection rate of less than 90%) was observed in 23% of studies in the analysis of hPIV-associated ALRI hospital admissions. We adjusted for underdetection as previously described by assuming the percent positivity for hPIV was the same in those tested and untested, and incorporated differences in rates of underdetection between studies in the meta-analyses of incidence and hospital admission rates.^15^ We did not adjust for underdetection when estimating hCFRs; the underdetection could cause an underestimation of hCFR estimates, because severe cases and deaths are usually less likely to be sampled compared with non-severe cases.^34^ We found a consistent result in our data showing that the hCFR in those tested was lower than in those untested (appendix p 29). Approximately 32% (12/38; appendix p 8) of studies with data on incidence and hospital admission rates used traditional test methods (eg, indirect immunofluorescence assay, culture, and mixed test methods). These traditional test methods have a similar specificity but lower sensitivity than molecular tests, and thus could result in an underestimation of the true burden.^35^, ^36^ Most hospital-based studies used nasopharyngeal specimens solely or in combination with other respiratory tract specimens (appendix pp 49–51), and it has been reported that nasopharyngeal specimens have similar or higher sensitivity in detecting hPIV than do other upper respiratory tract specimens.^37^, ^38^, ^39^, ^40^ Additionally, the inclusion of old data (34% [17 of 50 studies] of the data in the main analysis were from the pre-2010 period) could have biased our estimates. We chose 2010 as the cutoff because more studies have started to use molecular tests since the 2009 influenza pandemic. The included data suggest that approximately 81% (13/16) of hospital-based studies used molecular tests since 2010 compared with 50% (five of ten studies) before 2010. Estimation of inflation factors (and overall hPIV-associated ALRI mortality) in this study was based on the same data as we used previously (data on care-seeking for non-specific pneumonia symptoms and deaths) and so was susceptible to similar potential limitations and assumptions (appendix p 19).^15^

In general, our hPIV ALRI burden estimates, derived from laboratory-confirmed data, probably show the lower limit of the true burden of hPIV because of the challenge in systematically diagnosing and detecting hPIV infections, and many factors that can affect test results and lead to a false negative diagnosis. Additionally, the estimates of hospital admissions have probably underestimated the potential burden of hPIV-associated ALRI on health-care services, especially in regions with a low health-care capacity and poor access to care. Nevertheless, our hPIV-associated and hPIV-attributable ALRI burden estimates suggest that hPIV is a notable virus in childhood ALRI. These estimates should help to guide health investment priorities and resource allocation. By raising awareness of the disease and health-care burden these estimates might also encourage and inform investment to accelerate the development of targeted prevention and treatment interventions.

## Data sharing

The study-level data for all outcomes and detailed analyses are provided in the appendix. In conformity with the GATHER statement, all aggregate data included in this analysis (in an Excel format) will be made publicly available upon publication of the study on Edinburgh DataShare platform.

## Declaration of interests

YL reports grants from WHO outside the submitted work. MD-K reports grants from Merck and Pfizer, and personal fees from Merck outside the submitted work. SAM reports grants from the Bill & Melinda Gates Foundation, GlaxoSmithKline, Minervax, and Pfizer; and personal fees from the Bill & Melinda Gates Foundation outside the submitted work. CC reports grants from PATH, Sanofi Pasteur, and the US Centers for Disease Control and Prevention; and non-financial support (funds to travel to meeting) from Parexel during the conduct of the study. SRCH reports grants from Bill & Melinda Gates Foundation during the conduct of the study. HO reports grants from the Japan Agency for Medical Research and Development during the conduct of the study. EAFS reports grants, personal fees, and non-financial support (travel to Investigator meetings and to consultation meetings) from AstraZeneca, Merck, Pfizer, Regeneron, and Roche; personal fees from AbbVie, Alere, and Cidara; non-financial support (travel to meetings) from AbbVie and Novavax; other support fees for being on data and safety monitoring board from AbbVie and GlaxoSmithKline; and grants from Johnson and Johnson and Novavax, outside the submitted work. JAGS reports grants from the Bill & Melinda Gates Foundation, Gavi, The Vaccine Alliance, the UK Medical Research Council, the UK National Institute for Health Research, and the Wellcome Trust, outside the submitted work. L-MY reports grants from Japan Initiative for Global Research Network on Infectious Diseases and Agency for Medical Research and Development during the conduct of the study. HJZ reports grants from the Bill & Melinda Gates Foundation, the South Africa Medical Research Council, and the South Africa National Research Foundation, outside the submitted work. HC reports grants from the Bill & Melinda Gates Foundation, Johns Hopkins University, Sanofi, and WHO; and personal fees from the Bill & Melinda Gates Foundation, Johns Hopkins University, Sanofi, and WHO, during the conduct of the study. HN reports grants from the Bill & Melinda Gates Foundation and personal fees from the Bill & Melinda Gates Foundation during the conduct of the study; and grants from the Foundation for Influenza Epidemiology, Innovative Medicines Initiative, Sanofi, UK National Institute for Health Research, and WHO, and personal fees from AbbVie, Foundation for Influenza Epidemiology, Janssen, Reviral, and Sanofi, outside the submitted work. All other authors declare no competing interests.
