# Supplementary material for: Global burden of acute lower respiratory infection associated with human parainfluenza virus in children younger than 5 years for 2018: a systematic review and meta-analysis
Source: Lancet Glob Health. 2021 Jun 21;9(8):e1077–87. doi: 10.1016/S2214-109X(21)00218-7 (PMC8298256; doi:10.1016/S2214-109X(21)00218-7)
Supplement: Supplementary appendix [file mmc1.pdf]

THE LANCET
Global Health

Supplementary appendix

This appendix formed part of the original submission and has been peer reviewed. We post it as supplied by the authors.

Supplement to: Wang X, Li Y, Deloria-Knoll M, et al. Global burden of acute lower respiratory infection associated with human parainfluenza virus in children younger than 5 years for 2018: a systematic review and meta-analysis. *Lancet Glob Health* 2021; published online June 21. http://dx.doi.org/10.1016/S2214-109X(21)00218-7.

Supplementary materials for “Global burden of acute lower respiratory infections associated with human parainfluenza virus in children under five years in 2018: a systematic review and meta-analysis”

Contents

Appendix 1. Case definitions and glossary 3

Appendix 2. Search strategy for hPIV 5

Appendix 3. Characteristics of included studies 7

Appendix 4. Details of sensitivity analysis 10

Appendix 5. Details of adjustment for missing hPIV-4 17

Appendix 6. Overall hPIV-associated ALRI mortality 19

Appendix 7. ALRI burden attributable to hPIV 22

Appendix 8. Yearly variation in the hPIV-associated ALRI hospital admission rate 26

Appendix 9. Data imputation 27

Appendix 10. Adjusting for under-detection of hPIV 28

Appendix 11. Assessment tool for risk of bias in individual studies 30

Appendix 12. Risk of bias in included studies 32

Appendix 13. Details of individual studies 41

Appendix 14. Meta-estimates by narrow age groups 69

Appendix 15. Checklist of information that should be included in new reports of global health estimates 74

References 75

Appendix 1. Case definitions and glossary

Community-based studies: studies where eligible cases were actively identified through regular visits to households. We considered studies conducted in outpatient department or general practitioner in industrialised countries as good proxies of community-based studies.
Hospital-based studies: studies where children are enrolled when they are admitted into hospital.
Case definition for community-based studies
hPIV-associated ALRI: cough or difficulty breathing with increased respiratory rate for age (cut-offs same as in WHO IMCI case definition) AND laboratory confirmed hPIV.
hPIV-associated severe ALRI: children aged 2-59 months - cough or difficulty in breathing with chest wall indrawing AND laboratory confirmed hPIV; children aged <2 months - increased respiratory rate (>60 breaths/min) OR chest wall indrawing AND laboratory confirmed hPIV.
Case definition for hospital-based studies
Hospitalised hPIV-associated ALRI: all children with physician confirmed diagnosis of ALRI (pneumonia or bronchiolitis) that are hospitalised, or recommended hospital admission AND laboratory confirmed hPIV.
Hospitalised hPIV-associated ALRI with hypoxaemia: hospitalised ALRI cases with hypoxaemia (as defined below) AND laboratory confirmed hPIV.
Hypoxaemia: at altitude <=2500 m above seas level, SpO_2 <90% in children aged 1-59 months and <88% for neonates; at altitude >2500 m above sea level, SpO_2 <87% in children aged 1-59 months and <85% for neonates.
The relationship between the case definitions of different severity is displayed in Figure S1.1 A and B.

LIC: low income countries; LMIC: lower-middle income countries; UMIC: upper-middle income countries; HIC: high income countries as per World Bank Classification.
AFR: WHO African region; AMR: WHO Region of the Americas; EMR: WHO Eastern Mediterranean region; EUR: WHO European region; SEAR: WHO South-East Asian region; WPR: WHO Western Pacific region.
Neonates: children aged 0-27 days.
hPIV: human parainfluenza virus.
ALRI: acute lower respiratory infection.
hCFR: in-hospital case-fatality ratio.

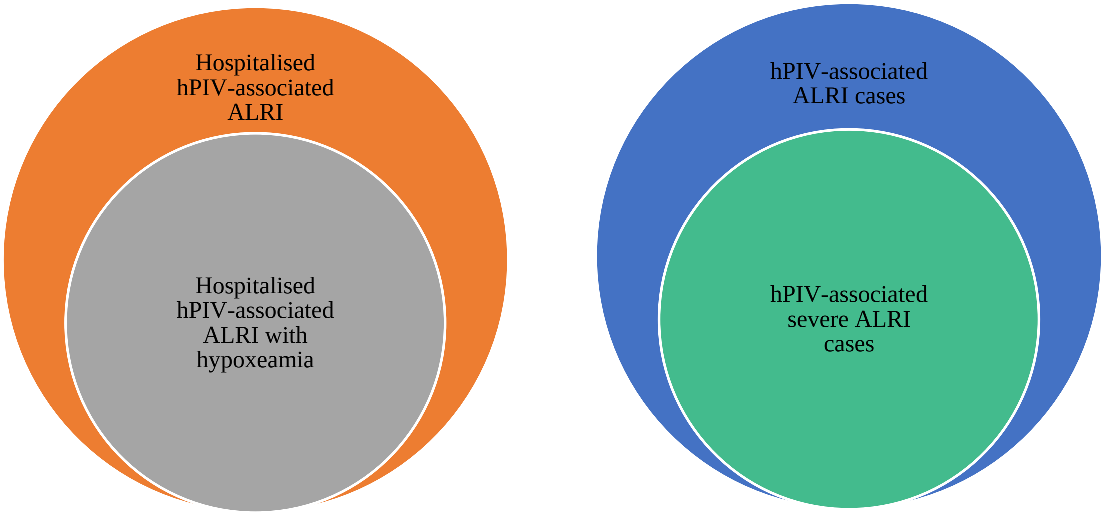

A B

Figure S1.1 The relationship between hospitalised hPIV-associated ALRI and hPIV-associated ALRI with hypoxaemia (A). The relationship between community hPIV-associated ALRI and hPIV-associated severe ALRI (B). The size of each circle is not proportionate to the number of cases for each severity.

Appendix 2. Search strategy for hPIV

Medline (Ovid)
1. exp Parainfluenza Virus 1, Human/ or exp Parainfluenza Virus 2, Human/ or exp Parainfluenza Virus 3, Human/ or exp Parainfluenza Virus 4, Human/ or exp Parainfluenza virus infection/ or infection, parainfluenza virus.mp. or infections, parainfluenza virus.mp. or virus infection, parainfluenza.mp. or virus infections, parainfluenza.mp. or parainfluenza vaccine.mp. or exp Parainfluenza Vaccines/ or PIV.mp. or Parainfluenza.mp.
2. metapneumovirus.mp. or exp metapneumovirus/ or hMPV.mp.
3. Bronchiolitis.mp. or exp Bronchiolitis/ or Bronchiolitis, Viral/
4. exp Respiratory Tract Diseases/
5. exp Respiratory Tract Infections/
6. acute respiratory infections.mp.
7. exp Pneumonia, Viral/ or *Pneumonia/ or exp Pneumonia/ or Pneumonia.mp.
8. acute lower respiratory infections.mp.
9. exp Incidence/ or exp Prevalence/ or exp morbidity/ or exp child mortality/ or exp infant mortality/ or exp hospital mortality/ or *hospital mortality/ or hospitalization rate.mp. or hospitalisation rate.mp. or exp Death/ or exp Cause of Death/ or burden.mp. /or proportion.mp.
10. 1 or 2
11. 3 or 4 or 5 or 6 or 7 or 8
12. 9 and 10 and 11
13. limit 12 to (humans and ("all infant (birth to 23 months)" or "newborn infant (birth to 1 month)" or "infant (1 to 23 months)" or "preschool child (2 to 5 years)"))
14. limit 14 to yr="1995 -2020"

Embase (Ovid)
1. exp parainfluenza vaccine/ or exp parainfluenza virus infection/ or para influenza virus.mp. or parainfluenza virus.mp. or parainfluenza viruses.mp. or Parainfluenzavirus.mp. or virus, parainfluenza.mp. or piv.mp. or exp Paramyxovirinae/
2. exp metapneumovirus/ or exp metapneumovirus infection/ or mpv.mp.
3. exp respiratory tract infection/ or exp pneumonia/ or exp bronchiolitis/ or exp viral bronchiolitis/
4. exp incidence/ or exp prevalence/ or exp morbidity/ or hospitalization rate.mp. or hospitalisation rate.mp. or exp hospital mortality/ or exp mortality/ or exp childhood mortality/ or exp infant mortality/ or exp death/ or exp child death/ or burden.mp./ or proportion.mp.
5. 1 and 3 and 4
6. 2 and 3 and 4
7. limit 5 to (human and (infant <to one year> or preschool child <1 to 6 years>))
8. limit 6 to (human and (infant <to one year> or preschool child <1 to 6 years>))
9. 7 or 8
10. limit 9 to yr="1995 -2020"

CINAHL
TI parainfluenza OR TI HPIV
TI metapneumovirus OR TI HMPV
AND
TI acute respiratory infection
AND
TI children
1995-2020

Global Health Library
(tw:(parainfluenza)) OR (tw:(piv)) OR (tw:(hpiv)) OR (tw:(metapneumovirus)) OR (tw:(mpv)) OR (tw:(hmpv)) AND (limit:("infant" OR "child, preschool" OR "child" OR "newborn"))
1995-2020

Web of Science
TITLE: (parainfluenza) OR TITLE: (HPIV) OR TITLE: (metapneumovirus) OR TITLE: (HMPV)
AND Title= (Acute Respiratory Infections) OR Title= (Pneumonia)
AND TOPIC: (children) OR TOPIC: (child) OR TOPIC: (infant)
1995-2020

Global Health (Ovid)
1. exp parainfluenza/ or exp parainfluenza viruses/ or exp human parainfluenza virus 1/ or exp human parainfluenza virus 2/ or exp human parainfluenza virus 3/ or exp human parainfluenza virus 4/ or piv.mp.
2. exp metapneumovirus/ or exp human metapneumovirus/ or metapneumovirus.mp.
3. exp respiratory diseases/ or exp bronchiolitis/ or exp lower respiratory tract infections/ or exp pneumonia/ or (respiratory diseases or lower respiratory tract infections).sh. or pneumonia.mp. or bronchiolitis.mp.
4. exp incidence/ or proportion.mp. or exp morbidity/ or hospitalization rate.mp. or hospitalisation rate.mp. or exp infant mortality/ or exp neonatal mortality/ or exp mortality/ or exp death/ or exp "causes of death"/
5. 1 or 2
6. 5 and 3 and 4
7. limit 6 to yr="1995 -2020"

CNKI
Topic: respiratory infections or pneumonia or bronchiolitis
And topic: parainfluenza virus or metapneumovirus
And topic: prevalence or deaths or incidence or disease burden or hospitalisation
And topic: children or infant
1995-2020

Wanfang
Topic: respiratory infections or pneumonia or bronchiolitis
And topic: parainfluenza virus or metapneumovirus
And topic: prevalence or deaths or incidence or disease burden or hospitalisation rate
And topic: children or infant
1995-2020

Chongqingvip
Any field: parainfluenza virus or metapneumovirus
AND title or key words: respiratory infection or respiratory tract infection or pneumonia or lung infection or severe pneumonia or bronchiolitis
AND title or key words: incidence or prevalence or death or hospitalisation or burden of disease
AND title or key words: children or infant.
1995-2020

Appendix 3. Summary of included studies and the overall approach

Table S3.1 Number of studies by age, region, and period for each outcome.

No of studies	Incidence rate of hPIV-associated ALRI	Hospital admission rate of hPIV-associated ALRI	Hospital admission rate of hPIV-associated ALRI with hypoxaemia	Proportion of hospitalised hPIV-associated ALRI	In-hospital case-fatality ratio of hPIV-associated ALRI
**All studies**	13	38	13	168	58
**From collaboration network**	5	19	13	37	30
**0-59 m**	12	34	13	94	46
**Reporting data by 0-5 m, 6-11 m, and 12-59 m**	6	26	13	78	27
**Developing countries**	8	28	13	143	53
**By World Bank income level**					
LIC	1	4	2	5	5
LMIC	6	10	5	26	23
UMIC	1	10	6	101	19
HIC	5	14	0	36	11
**By WHO region**					
AFR	1	11	5	17	16
AMR	2	9	2	19	11
EMR	1	1	1	16	8
EUR	1	4	0	15	3
SEAR	6	6	3	14	8
WPR	2	7	2	87	12
**By median study year**					
~2005	3	7	1	34	9
2006-2010	3	11	2	41	15
2011~	5	20	11	93	33
**No of hPIV-associated ALRI cases**					
0-99	9	24	NA	109	40
100-199	2	7	NA	29	10
200- ~	1	7	NA	30	8

Table S3.2 Number of studies that were included in the main analysis by risk of bias for each outcome.

No of studies	Incidence rate of hPIV-associated ALRI	Hospital admission rate of hPIV-associated ALRI	Hospital admission rate of hPIV-associated ALRI with hypoxaemia	Proportion of hospitalised hPIV-associated ALRI	In-hospital case-fatality ratio of hPIV-associated ALRI
**All studies**	12	26	13	94	27
Study design	12	25	13	69	27
Adjustment for healthcare utilization	..	17	6	..	..
Patient groups excluded	8	18	8	72	16
Case definition	10	19	12	51	25
Sampling strategy	5	22	11	63	25
Test method	8	18	10	64	..
Hypoxaemia ascertainment	..	..	10	..	..

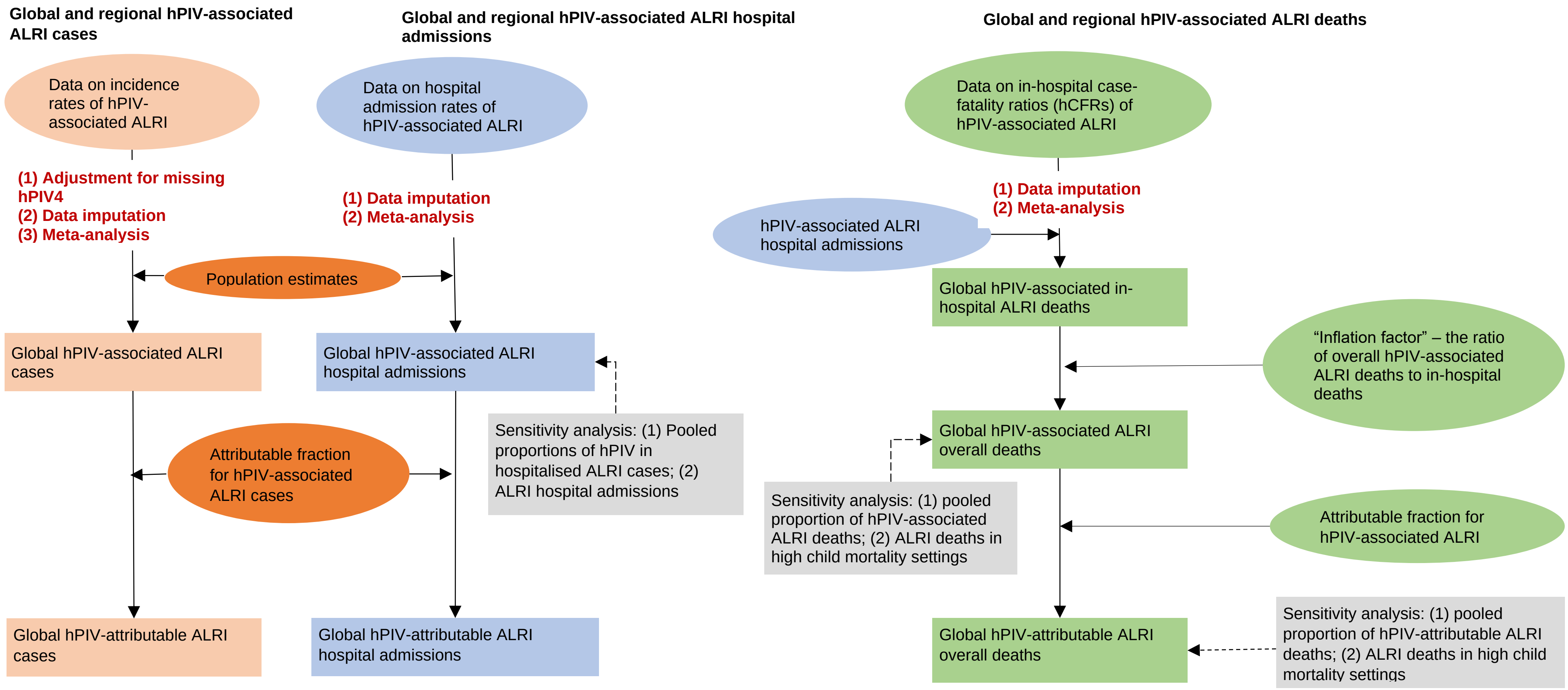

We report the global estimates of hPIV-associated ALRI cases, hPIV-associated ALRI hospital admissions, and hPIV-associated ALRI deaths in hospital and overall deaths (in-hospital and out-hospital). This figure summarises our approach for each outcome and also shows how they relate to each other. The oval shapes show input data and the square shapes show outputs. The solid lines show the main analyses and dashed lines show the sensitivity analyses. Global hospital admissions of hPIV-associated ALRI were estimated using an incidence-based approach in the main analysis (a proportion-based approach in the sensitivity analysis). hPIV-associated in-hospital ALRI deaths were estimated by combining hCFRs and hospital admissions of hPIV-associated ALRI. The overall mortality of hPIV-associated ALRI was estimated using the "inflation factor" approach in the main analysis. The inflation factor, defined as the ratio of overall hPIV-associated ALRI deaths to in-hospital deaths, was applied to the in-hospital mortality. In a sensitivity analysis, we estimated the overall hPIV-associated ALRI deaths by combining the proportion of hPIV-associated ALRI deaths and the total ALRI mortality for children aged 0-59 months. For hPIV-attributable morbidity and mortality burden, we applied the attributable fraction to the associated burden estimates in the main analysis. In a sensitivity analysis for hPIV-attributable mortality, we combined the proportion of hPIV-attributable ALRI death and the total number of ALRI deaths. hPIV: human parainfluenza virus. ALRI: acute lower respiratory infection. hCFR: in-hospital case-fatality ratio.

Figure S3.1. Approaches for global human parainfluenza-virus-associated ALRI and human parainfluenza-virus-attributable morbidity and mortality estimation in children under five years

Appendix 4. Details of sensitivity analysis

In this section, we present the pooled estimates adjusted for missing hPIV-4 where applicable. We assessed how the global burden estimates changed in different stratification groups or different approaches.

In Table S4.1, we estimated global hPIV-associated ALRI hospital admissions by summing up the estimates by World Bank income level, country development status, WHO regions where available.

In Table S4.2A and S4.2B, we estimated the hPIV-associated ALRI hospital admissions using a proportion-based approach. For the proportion-based approach, we applied the proportion of hPIV positives in hospitalised ALRI to the global ALRI hospital admissions for 0-59 months.[1,2]

In Table S4.3, we estimated global hPIV-associated ALRI mortality by summing up the estimates by World Bank income level, country development status, WHO region where available.

In Table S4.4, we estimated the number of hPIV-severe ALRI cases for 0-59 months in the community for high child mortality settings. We did not identify relevant data in low child mortality settings, so we were unable to estimate the number of hPIV-severe ALRI cases for this setting.

Table S4.1A Adjusted hospital admission rates (per 1,000 children per year) and hospital admissions for hPIV-associated ALRI by World Bank income level and country development status*†

Age		LMIC	UMIC	HIC	Global estimates by income levels	Developing	Industrialised	Global estimates by development status
**0-5 m (A)**	No. of studies	7	6	4		14	3	
	Hospital admission rate (/1,000)	3.8 (1.8-7.8)	5.7 (3-10.5)	5.5 (3.1-9.9)		4.9 (3-7.9)	3.7 (3-4.5)	
	Hospital admissions (*1,000)	168 (81-349)	105 (56-196)	35 (20-62)	308 (157-607)	304 (188-493)	26 (21-32)	330 (209-524)
**6-11 m (B)**	No. of studies	7	5	3		13	2	
	Hospital admission rate (/1,000)	3.5 (1.7-7)	3.8 (1.9-7.6)	3.5 (1.9-6.5)		3.8 (2.4-6.1)	2.5 (1.7-3.7)	
	Hospital admissions (*1,000)	154 (76-311)	70 (35-139)	22 (12-41)	246 (123-491)	234 (147-373)	17 (12-26)	252 (159-398)
**12-59 m (C)**	No. of studies	8	8	4		19	1	
	Hospital admission rate (/1,000)	0.8 (0.4-1.4)	0.8 (0.4-1.6)	0.8 (0.2-2.9)		0.8 (0.5-1.3)	..	
	Hospital admissions (*1,000)	274 (147-512)	117 (59-233)	41 (11-153)	432 (217-898)	388 (241-623)	..	..
**0-59 m (A+B+C)**	Hospital admissions (*1,000)	596 (304-1171)	292 (150-569)	98 (42-257)	986 (497-1997)	926 (576-1489)	..	..

* Hospitalisation rates from meta-analysis. Global estimates were calculated as the sum of estimates by age and regions.
† Hospitalisation rates were adjusted for missing hPIV-4.

Table S4.1B Adjusted hospital admission rates (per 1,000 children per year) and hospital admissions for hPIV-associated ALRI by WHO regions*†

Age		AFR	AMR	EMR	EUR	SEAR	WPR
**0-5 m (A)**	No. of studies	7	3	1	1	2	3
	Hospital admission rate (/1,000)	5.9 (3.7-9.5)	3.5 (2.7-4.4)	..	..	2.8 (0.7-11.6)	11.8 (9-15.5)
	Hospital admissions (*1,000)	104 (65-166)	26 (21-34)	..	..	50 (12-200)	144 (110-188)
**6-11 m (B)**	No. of studies	7	2	1	1	1	3
	Hospital admission rate (/1,000)	4.1 (2.7-6.1)	1.9 (1.4-2.6)	..	..	..	8.0 (6.1-10.6)
	Hospital admissions (*1,000)	71 (47-107)	14 (11-19)	..	..	..	97 (74-128)
**12-59 m (C)**	No. of studies	8	4	0	1	4	3
	Hospital admission rate (/1,000)	0.8 (0.4-1.6)	0.8 (0.3-2.2)	..	..	0.3 (0.2-0.6)	1.6 (0.5-5.5)
	Hospital admissions (*1,000)	107 (54-214)	48 (18-129)	..	..	42 (24-72)	154 (47-509)
**0-59 m (A+B+C)**	Hospital admissions (*1,000)	283 (166-487)	89 (49-182)	..	..	..	395 (230-825)

* Hospitalisation rates from meta-analysis. Global estimates were calculated as the sum of estimates by age and regions.

† Hospitalisation rates were adjusted for missing hPIV-4.

Table S4.2A. Adjusted proportion of hospitalised hPIV-associated ALRI for 0-59 months by World Bank income level*†

	No. of studies	Proportion (%) for 0-59 m
**All studies for 0-59 months**	94	8.8 (7.6-10.2)
**By World Bank income group**		
Low income (L)	4	11.1 (6.1-19.3)
Middle income (M)	71	9.1 (7.6-11.0)
High income (H)	19	7.1 (5.4-9.1)

Table S4.2B. The adjusted hospital admissions of hPIV-associated ALRI for 0-59 months using the proportion-based approach

No. of studies	Proportion (%) for 0-59 m	Hospital admissions of all-cause ALRI (thousand)	Hospital admissions of hPIV-associated ALRI (thousand)
94	8.8 (7.6-10.2)	5,133 – 16,400[1,2]	452– 1,443

* Proportions from meta-analysis.
† Proportions were adjusted for missing hPIV-4.

Table S4.3A. Adjusted hCFRs (%) and in-hospital deaths of hPIV-associated ALRI by different stratification groups.*†

Age		LMIC	UMIC	HIC	Global by income group	Developing	Industrialised	Global by country development status
	No. of studies	15	8	4		25	2	
0-5 m (A)	hCFR (%)	3.9 (2.1-7.3)	2.4 (1.3-4.6)	0.9 (0.2-3.6)		3.2 (2.0-5.0)	1.0 (0.3-3.9)	
	Deaths	6600 (2600-17000)	2500 (1100-6100)	300 (100-1500)	9400 (3700-24500)	9700 (5100-18900)	300 (100-900)	10000 (5200-19600)
6-11 m (B)	hCFR (%)	3.5 (2.2-5.6)	1.9 (0.8-4.1)	0.9 (0.4-1.9)		2.6 (1.2-5.8)	1.3 (0.3-4.9)	
	Deaths	9600 (4500-20900)	2200 (800-6400)	400 (100-1700)	6000 (1900-20800)	6100 (2400-14900)	200 (100-900)	6300 (2500-15900)
12-59 m (C)	hCFR (%)	2 (0.5-7.4)	3.8 (2.2-6.6)	1.2 (0.3-4.7)		2.7 (1.8-4.1)	0.9 (0.4-2.0)	
	Deaths	3100 (700-13800)	2700 (1100-6400)	300 (100-1200)	12200 (5300-28800)	10500 (5600-19500)	..‡	..
0-59 m (A+B+C)	Deaths	19400 (7800-50800)	7400 (3000-18900)	1000 (200-4100)	27800 (11000-73700)	26400 (13300-53000)	..	..

* hCFR estimates from meta-analysis. Global estimates were calculated as the sum of estimates by age and regions.
† hCFR estimates were adjusted for missing hPIV-4.
‡ There was only one study with hPIV-associated ALRI hospitalisation rates for the stratum, thus we were unable to estimate the hPIV-associated ALRI hospitalisations and in-hospital deaths.

Table S4.3B. Adjusted hCFRs (%) and in-hospital deaths of hPIV-associated ALRI by WHO regions.[*†]

Age		AFR	AMR	EMR	EUR	SEAR[‡]	WPR
	No. of studies	11	5	1	1	4	5
0-5 m (A)	hCFR (%)	3.2 (1.9-5.3)	1.2 (0.5-3.2)	..	..	..	3.4 (0.1-63.5)
	Deaths	3300 (1700-6600)	300 (100-800)	..	..	..	4900 (200-118000)
6-11 m (B)	hCFR (%)	3.4 (1.3-8.2)	3.2 (1.7-6)	..	..	..	1.8 (0.3-11.6)
	Deaths	2400 (900-6500)	500 (200-900)	..	..	..	1700 (300-10800)
12-59 m (C)	hCFR (%)	3 (1.7-5.3)	1.4 (0.8-2.6)	..	..	..	2.4 (0.6-9.2)
	Deaths	3200 (1300-7800)	700 (200-2100)	..	..	..	3700 (600-22200)
0-59 m (A+B+C)	Deaths	9000 (4000-20700)	1500 (600-3700)	..	..	..	10900 (1200-149500)

[*] hCFR estimates from meta-analysis. Global estimates were calculated as the sum of estimates by age and regions.
[†] hCFR estimates were adjusted for missing hPIV-4.
[‡] We were unable to calculate hCFR meta-estimates in SEAR as the four studies all reported zero death.

Table S4.4. Adjusted incidence and number of hPIV-associated severe ALRI cases for 2018 in high child mortality setting. [*†]

Age		High child mortality setting
0-5 m (A)	No. of studies	4
	Incidence rate (/1,000)	20.6 (2.8-134.8)
	Cases (*1,000)	948 (138-6512)
6-11 m (B)	No. of studies	3
	Incidence rate (/1,000)	30.7 (8.3-106.6)
	Cases (*1,000)	1400 (393-4985)
12-59 m (C)	No. of studies	3
	Incidence rate (/1,000)	8.1 (2.2-29.8)
	Cases (*1,000)	2887 (790-10557)
0-59 m	No. of studies	5 (2)
	Incidence rate (/1,000)	9.3 (3.5-24.9)
	Cases (*1,000)	4190 (1585-11083)

[*] Incidence rates from meta-analysis.
[†] Incidence rates were adjusted for missing hPIV-4.

Appendix 5. Details of adjustment for missing hPIV-4

Since we included a mix of three-type (hPIV-1 to hPIV-3) and four-type (hPIV-1 to hPIV-4) data, we adjusted for missing hPIV-4 as shown in Figure S5.1 and Figure S5.2.

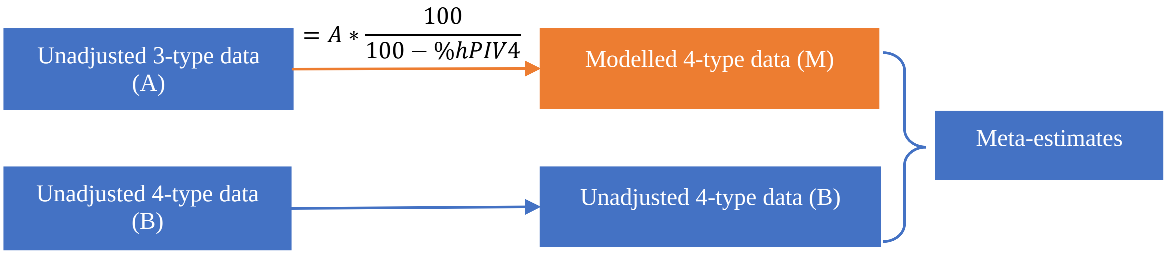

Figure S5.1. Adjustment for the missing hPIV-4 in incidence rates and hospital admission rates of hPIV-associated ALRI for children under five years.

Table S5.1. Prevalence of each hPIV type for children aged 0-59 months.[*]

	**Prevalence of hPIV types**
No. of studies	24
Prevalence of hPIV-1 (%)	26.7 (22.1-31.9)
Prevalence of hPIV-2 (%)	9.5 (6.7-13.2)
Prevalence of hPIV-3 (%)	49.9 (43.6-56.2)
Prevalence of hPIV-4 (%)	12.0 (8.2-17.2)

Table S5.2. Prevalence of each hPIV type in hPIV-associated ALRI deaths for children aged 0-59 months.[†]

	**Prevalence of hPIV cases (%)**	**hCFRs (%)**	**Prevalence of hPIV deaths (%)[‡]**
hPIV-1	26.7	9.4 (5.4-15.8)	36.9
hPIV-2	9.5	9.1 (3.8-20.1)	12.6
hPIV-3	49.9	6.0 (3.4-10.3)	44.1
hPIV-4	12.0	3.7 (0.6-19.1)	6.5

[*] Data were from hospital-based studies; data were eligible if there were at least five hPIV-associated ALRI cases, and four hPIV types were detected.

[†] Data were from five hospital-based studies where at least 90% of cases were tested, and there were at least five hPIV-positive ALRI deaths. Data were from Zambia, South Africa, Mali, Morocco, Philippines.

[‡] Calculated based on the prevalence and hCFRs of four hPIV types. So hPIV-4 accounted for 6.5% of hPIV-associated ALRI deaths, and hPIV-1 to hPIV-3 accounted for 93.5%.

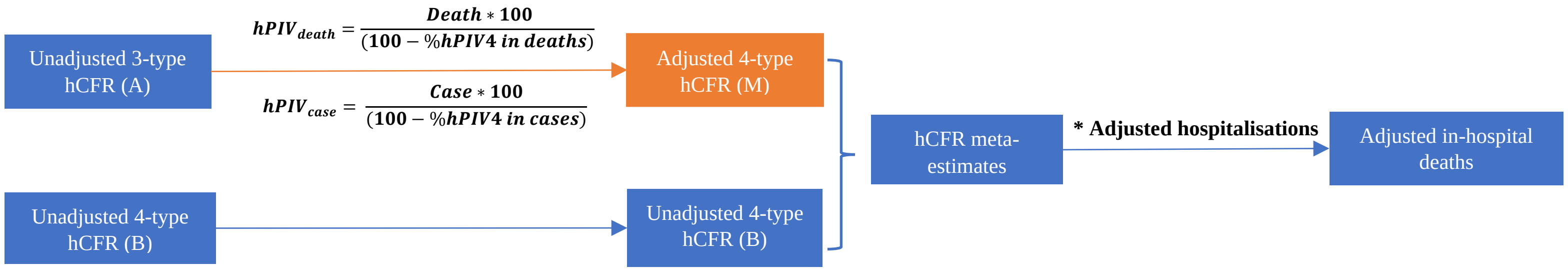

Figure S5.2. Adjustment for missing hPIV-4 in hCFRs of hPIV-associated ALRI for 0-59 months.

Table S5.3. Unadjusted and adjusted burden estimates of hPIV-associated ALRI for children under five years by outcome.

	Unadjusted estimates	Adjusted estimates
**Global hPIV-associated ALRI cases in the community (thousand)**	22140 (UR 16132-31951)	25636 (UR 17694-38599)
**Global hPIV-associated ALRI hospital admissions (thousand)**		
Using the incidence-based approach	947 (UR 561-1644)	1007 (UR 601-1,750)
Using the proportion-based approach	411-1,312	452-1,443
**Global hPIV-associated ALRI in-hospital deaths**	26100 (UR 12900-54200)	25700 (UR 12000-56500)

Appendix 6. Overall hPIV-associated ALRI mortality

(1) "inflation factor" approach - main analysis

The inflation factor of hPIV-associated ALRI in-hospital deaths was estimated by child mortality settings. For high child mortality settings, the details of the data for inflation factor estimation were described previously.[3] Eight sites reported (1) the number of pneumonia deaths occurring in-hospital and (2) the number of deaths occurring out-hospital for the same observation period were obtained. We divided the overall pneumonia deaths by in-hospital pneumonia deaths at each site, and used the median ratio across sites as a proxy for the inflation factor for hPIV-associated ALRI deaths for high child mortality settings (Table S6.1). Using this approach, we assumed that the hPIV prevalence in community ALRI deaths was the same as that in fatal hospitalised ALRI cases.

For low child mortality settings, the inflation factor was estimated using the measure for childhood pneumonia care-seeking: the proportion of children with pneumonia symptoms who received care at health providers as measured in Multiple Indicator Cluster Surveys, Demographic and Health Surveys, and other national surveys.[4] In this analysis, the reciprocal of percent of children with pneumonia symptoms who received care at health providers was estimated and used as a proxy for the inflation factor. The median inflation factor across regions and countries was applied to the in–hospital mortality estimate for low child mortality countries to yield the overall mortality of virus–associated ALRI deaths in that setting.

Assumptions and potential limitations

For high child mortality settings, the inflation factor estimate was based on limited data, and was extrapolated to other high child mortality countries and regions, which could lead to bias. The overall mortality estimates might be biased if hPIV-associated ALRI deaths are more or less likely to occur in hospitals compared with ALRI deaths due to other pathogens. For low child mortality countries, the inflation factor was estimated using the proportion of children with pneumonia symptoms seeking care. Using this measure, the inflation factor and overall mortality estimate is likely to be underestimated because the definition of "care–seeking" is broader than the definition of "in–hospital": contact with primary care is included as "care–seeking" in surveys, but are not included in the "in–hospital mortality" estimates in the present analysis. The US vital statistics data show that about 40% of under–five ALRI deaths (ICD–10 J09–22; U04) occurred in outpatient or emergency departments during 2010–2017.[5] Additionally, this analysis was based on one further assumption that CFR for hospitalised pneumonia cases was the same as CFR for pneumonia not hospitalised. The direction of bias related to this assumption could be difficult to determine: children with more severe symptoms are more likely to receive hospital care than those without; on the other hand, supportive care in hospitals can reduce the risk of death, and lack of appropriate care or delays in care can lead to rapid deterioration.[6-8]

Table S6.1. Main analysis - Number of all-cause pneumonia deaths in-hospital and out-hospital, and overall hPIV-associated ALRI mortality estimation.

Setting	Site	Ratio of all pneumonia deaths over in-hospital deaths for 0-59 months (A)	Inflation factor (B=median of A)	In-hospital mortality of hPIV-associated ALRI for high child mortality settings (C)	Overall hPIV-associated ALRI mortality for high child mortality settings (D=B*C)
**High child mortality settings**	Nairobi, Kenya (urban), 2008, 2010-2015	1.7	2.2	21600 (10600-44100)	47600 (UR 23400-97100)
	Siaya, Kenya (rural), 2011–2016	3.5			
	Nouna, Burkina Faso (rural), 2014–2016	1.5			
	Dodowa, Ghana (rural), 2011–2015	2.1			
	Manhiça, Mozambique (mixed), 2012–2016	2.8			
	Agincourt, South Africa (rural); 2010–2015	2.3			
	Mirzapur, Bangladesh (rural), 2008–2012 [9]	2.5			
	Multi–sites, Bangladesh (mixed), 2010–2012 [10*]	1.8			
**Low child mortality settings**	28 countries and regions [4]	Range from 1.1 to 4.5	1.3	4100 (1400-12400)	5300 (UR 1800-16200)
**Global estimates**[†]					53000 (UR 25300-113500)

Table S6.2. Proportion of children with pneumonia symptoms seeking care by country. [4]

Country	Data Source	Year	Care seeking (%)
Albania	DHS_2008-2009	2009	70
Argentina	MICS_2011-2012	2012	94
Armenia	DHS _2010	2010	57
Belarus	MICS_2012	2012	93
Belize	MICS_2011	2011	82
Bosnia and Herzegovina	MICS_2011-2012	2012	87
Brazil	MoH_PNDS_2006	2006	50
Colombia	DHS_2010	2010	64
Costa Rica	MICS_2011	2011	77
Cuba	MICS_2014	2014	93
El Salvador	MICS and MDG indicators(Prelim)_2014	2014	80
Georgia	MICS_2005	2005	74
Iran (Islamic Republic of)	IrMIDHS(Prelim)_2010-2011	2010	76
Jamaica	MICS_2011	2011	82
Jordan	DHS_2012	2012	77
Kazakhstan	MICS_2010-2011	2011	81
Lebanon	MICS_2000	2000	74
Maldives	MICS_2001	2001	22
Mongolia	MICS_2013-2014	2014	70
Montenegro	MICS_2005	2005	89
Panama	MICS(prelim)_2013	2013	82
Peru	DHS_2014	2014	60
Republic of Moldova	MICS_2012	2012	79
Serbia	MICS_2010	2010	90
Sri Lanka	DHS_2006-2007	2007	58
Thailand	MICS_2012	2012	83
The former Yugoslav Republic of Macedonia	MICS_2005-2006	2006	93
Ukraine	MICS_2012	2012	92

[*] Including ARI deaths identified by community survey. ARI deaths were defined as for children under 5 years, sudden onset cough or difficulty in breathing within 2 weeks of death.
[†] Global estimates are the sum of estimates by child mortality setting.

(2) Sensitivity analysis of hPIV-associated ALRI overall mortality in high child mortality settings based on the proportion positives of hPIV in ALRI deaths

In sensitivity analysis, we estimated the overall hPIV-associated ALRI mortality for the high child mortality setting using the following formula:

$$\text{Overall hPIV\_associated ALRI mortality} = \%hPIV\ in\ ALRI\ deaths * ALRI\ mortality$$

We estimated the proportion of hPIV positives in ALRI deaths using data from 12 hospital-based studies (including five PERCH sites) from high mortality burden settings in which at least 90% of ALRI cases were tested and at least five ALRI deaths were identified during the study period. All the studies detected four hPIV types. Neonatal hPIV-associated ALRI deaths were reported. The proportion of hPIV was estimated for the overall age band (i.e., 0-59 months) as the data were insufficient to allow disaggregation by narrower age bands (e.g., 0-27 d and 1-59 m).

Table S6.2. Using the proportion of hPIV-associated ALRI deaths based on hospital-based studies to estimate the overall hPIV-associated ALRI mortality for children aged 0-59 months in high child mortality settings.

	**hPIV-associated ALRI deaths (A)**	**ALRI deaths (B)**	**% of hPIV positives in ALRI deaths (C=A*100/B)**	**2017 ALRI deaths for high child mortality settings (D)**	**Overall hPIV-associated ALRI deaths for high child mortality settings (E=C*D/100)**
12 hospital-based studies (including five PERCH sites) *	42	584	7.3% (95%CI 4.6-11.3)	769,712	56,100 (UR 36,500-87,400)

* We did meta-analysis of 12 studies from Philippines, Bangladesh, Gambia, Zambia, Mali, Kenya, South Africa, and Mozambique. The percent was 9.4% (95%CI 6.6-13.3) when only pooling five PERCH sites in Gambia, Zambia, Mali, Kenya, and South Africa.

Appendix 7. ALRI burden attributable to hPIV

1. Estimating the attributable fraction for hPIV-associated ALRI cases and hospital admissions

We estimated the hPIV-attributable burden by applying the attributable fraction of hPIV-associated ALRI cases to the number of hPIV-associated ALRI cases/hospital admissions. Since the attributable fraction varies by hPIV types, we estimated the average attributable fraction by accounting for the prevalence of each hPIV type using formula 7.1. In the formula, %hPIVi denote the prevalence of hPIV-1 to hPIV-4; AFi denote the AF for each type. Input data and final estimates are listed in Table S7.1. Type-specific odds ratios are from two pooled analyses of multi-country data.[11,12]

Formula 7.1 - $AFEaverage = \sum_{i}^{4} \%\, hPIVi * AFi$

Table S7.1. The estimation of the average attributable fraction for hPIV-associated ALRI cases.

	Odds ratio (OR)[11,12]	AFi (%) = (OR-1)*100/OR*	% hPIVi	Average AF (%)
hPIV1	7.5	87%	27	72%
hPIV2	1.0 ~ 2.0	25%	10	
hPIV3	2.6 ~ 6.7	79%	50	
hPIV4	1.7 ~ 2.6	55%	12	

* We used the median value of the ORs which were available in two pooled analysis of multi-country data.

2. Estimating the attributable fraction for hPIV-associated ALRI mortality

The hPIV-attributable ALRI mortality was estimated by combining the hPIV-associated ALRI mortality and the attributable fraction of hPIV-associated ALRI deaths. The attributable fraction was estimated by assuming the hCFR for ALRI cases unattributed to hPIV was the same with the hCFR for hPIV–negative cases (Figure S7.1).

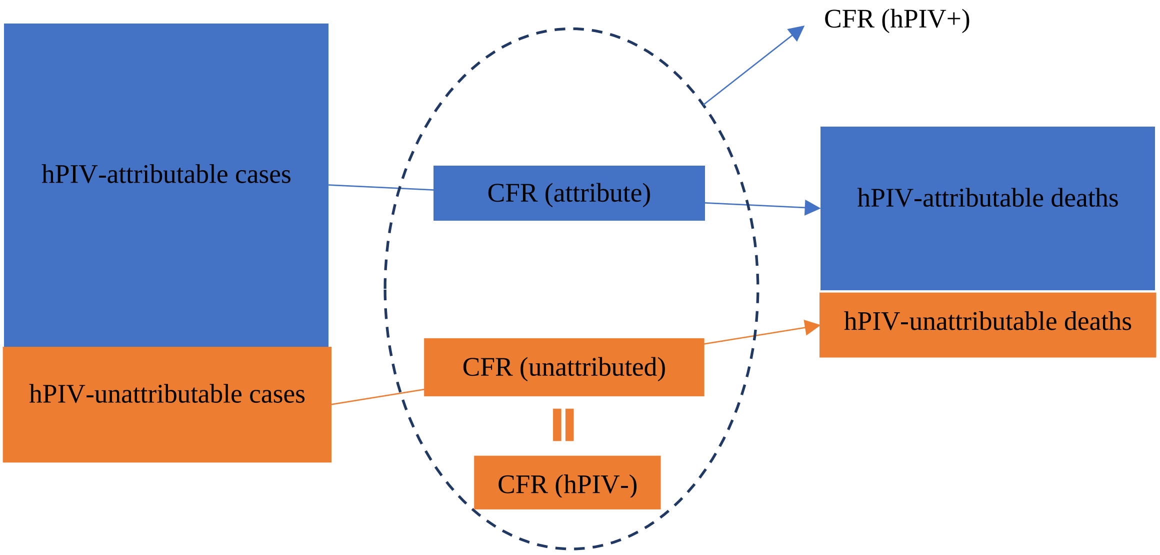

Figure S7.1. Estimating the attributable fraction of hPIV-associated ALRI deaths for children under five years.

As shown in the above figure, the key was to split the CFR for hPIV-associated ALRI cases into (1) the CFR for hPIV-attributable ALRI cases; (2) the CFR for hPIV-unattributable ALRI cases. We assumed the CFR for hPIV-unattributable cases was equal to the CFR for hPIV-negative cases. The AF for hPIV-associated ALRI deaths was estimated based on the following formulas:

Formula A - $Deaths\ (hPIV+) = Cases(hPIV+) * hCFR(hPIV+)$

Formula B - $Deaths\ (hPIVattri) = [Cases(hPIV\ +) * \frac{AFcase(\%)}{100}] * hCFR(hPIVattri)$

The Deaths (hPIV+) and Cases (hPIV+) represented for the number of ALRI deaths and cases positive for hPIV; the hCFR (hPIV+) represented for the in-hospital case-fatality ratio for hPIV positive ALRI cases. Similarly, the Deaths (hPIVattri), Cases (hPIVattri), and hCFR (hPIVattri) represented for the measures for cases or deaths attributed to hPIV. The AFcase represented for the AF (%) for hPIV-associated ALRI cases. By definition, the AF (%) for hPIV-associated ALRI deaths could be estimated using formula A and B as follows:

Formula C (Formula B/A) - $AFdeaths(\%) = AFcase(\%) * \frac{hCFR(hPIVattri)}{hCFR(hPIV+)}$

Here, we used the average AF of hPIV cases as the input for AFcases (%). Then we estimated the ratio of case-fatality of hPIV-attributable ALRI cases versus hPIV-positive cases using the formula below. The association between hCFR (hPIVattri), hCFR (hPIVnon-attri), and hCFR (hPIV+) is shown in Formula D and E:

Formula D - $Deaths\ (hPIV\ +) = Deaths\ (hPIVattri) +\ Deaths(hPIVnon - attri)$

$$= [Cases(hPIV\ +) * \frac{AFcase(\%)}{100}] * hCFR(hPIVattri) + [Cases(hPIV\ +) * \frac{100 - AFcase(\%)}{100}] * hCFR(hPIVnon - attri)$$

Formula E (transformed from Formula D) -

$$hCFR\ (hPIV\ +) = \frac{AFcase(\%)}{100} * hCFR(hPIVattri) + \frac{100 - AFcase(\%)}{100} * hCFR(hPIVnon - attri)$$

Table S7.2. Estimation of the attributable fraction for hPIV-associated ALRI deaths for 0–59 months.[*]

hCFR (%) in hPIV-positive cases (A)	hCFR (%) in hPIV-negative cases (B)	Ratio of case-fatality for hPIV-unattributable cases versus hPIV-associated cases (C=B/A)	Ratio of case-fatality for hPIV-attributable cases versus hPIV-associated cases (D, estimated using A and C)	AF for hPIV-associated ALRI deaths (=AF for hPIV-associated ALRI cases * D)[†]
4.1 (2.3-6.9)	4.8 (3.2-7.3)	1.2	0.9	65%

[*] We only included hospital-based studies with high level of testing (>=90% of ALRI cases were tested) and with at least five ALRI deaths to ensure the precision of estimates. We did the meta-analysis of 12 studies from Philippines, Bangladesh, Gambia, Zambia, Mali, Kenya, South Africa, and Mozambique. The ratio was 0.75 when only including five PERCH sites in Gambia, Zambia, Mali, Kenya, and South Africa.
[†] We used the average AF for hPIV-associated ALRI cases (72%), as calculated in Table S7.1.

3. Estimating hPIV-attributable ALRI mortality in high child mortality settings using CHAMPS data

CHAMPS investigates the causes of under-five mortality at seven sites in sub-Saharan Africa and South Asia, which are from high child mortality settings.[13] For this analysis, we extracted the number of all-cause ALRI deaths and the number of hPIV-attributable ALRI deaths, where ALRI and hPIV could be anywhere in the causal pathway (including underlying cause or condition, immediate cause or condition, co-morbid causes or conditions) for the period December 2016 to October 2019.[14] The input data for this analysis and the results are in Table S7.3.

Table S7.3. Estimating hPIV-attributable ALRI mortality using CHAMPS data

	**hPIV-associated ALRI deaths (A)** *	**ALRI deaths (B)** †	**% of hPIV-attributable ALRI (C=A*100/B)**	**2017 ALRI deaths for high child mortality settings (D)**	**hPIV-attributable ALRI deaths for high child mortality settings (E=C*D/100)**
0-27 days	2	91	2.2% (95%CI 0.3-7.7)	146,967	3,200 (UR 700-16,800)
1-59 months	13	191	6.8% (95%CI 3.7-11.4)	622,742	42,300 (UR 24,300-74,900)
0-59 months					45,500 (UR 24,900-91,700)

* hPIV as any of immediate, co-morbid, and underlying cause of death (hPIV appeared anywhere in the causal chain of deaths).

† ALRI as any of immediate, co-morbid, and underlying cause of death (ALRI appeared anywhere in the causal chain of deaths).

Appendix 8. Yearly variation in the hPIV-associated ALRI hospital admission rate

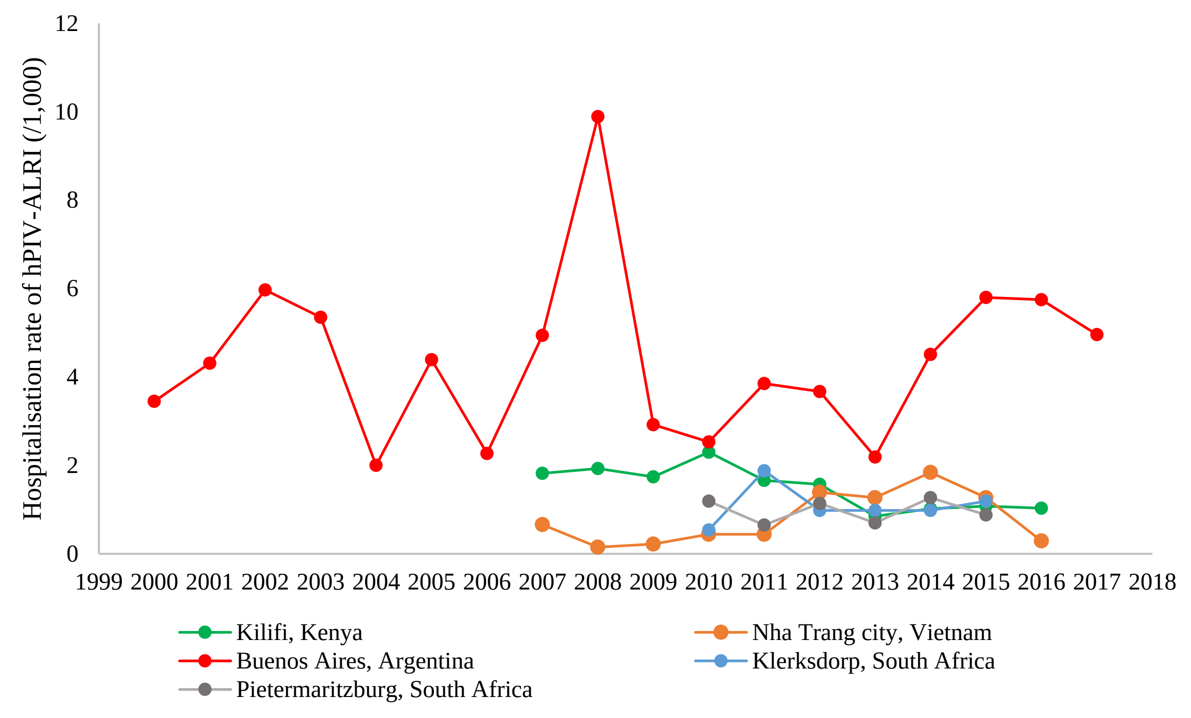

Figure S8.1. Yearly unadjusted hospital admission rates of hPIV-associated ALRI in children under five years (with at least five years' data).

Table S8.1. The average hPIV-associated ALRI hospital admission rates (unadjusted) in the above five studies for 0-59 months

Location	Period	Average rate (per 1,000 children per year)
Nha Trang, Vietnam	2007-2010	0.4
	2011-2014	1.1
Kilifi, Kenya	2007-2010	2.0
	2011-2016	1.3
Klerksdorp, South Africa	2010-2012	1.1
	2013-2015	1.0
Pietermaritzburg, South Africa	2010-2012	0.8
	2013-2015	0.9
Buenos Aires, Argentina	2001-2009	4.5
	2010-2017	4.2

Appendix 9. Data imputation

Several studies reported data for 0–11 months, 0–23 months, and 0–35 months; to incorporate the information from these studies, the missing incidence rate for 0–59 months was imputed. The imputation was done as previously[15]: (1) imputing the denominator; (2) imputing the rate; (3) calculating the case number using the denominator and rate. Steps (2) and (3) were skipped if the case number was available. The reference group referred to the age group with available rate data and could be one of 0–35 months, 0–23 months or 0–11 months. When two or more age groups were available, the reference group was chosen in the following order: 0–35 months, 0–23 months, and 0–11 months. Details of each step are:

(1) The denominator was imputed by country income regions based on the probability of dying between age n and n+x (nqx) obtained from WHO life tables.[16] The proportion of total under–five population that are in the reference age group was calculated using the nqx estimates. Using this proportion and the denominator in the reference group, the denominator for 0–59 months was estimated.

(2) The case number was imputed using a multiple imputation approach assuming the rates for 0–59 months were missing at random.[17] Figure S8.1 shows the process. First, we estimated the rate ratios between 0–59 months and any of 0–11 months, 0–23 months, and 0–35 months (meta–analysis was only done when there were three or more studies). Second, the pooled rate ratio was assumed to follow a log–normal distribution, and 100 samples of rate ratios were simulated. Third, 100 samples of rates for 0–59 months were generated based on the rate in the reference group and the corresponding rate ratios. Fourth, case numbers were calculated using the denominator and imputed rates. Using the method, 100 datasets of imputed case numbers were generated. Fifth, meta–analysis was done for each dataset, and the meta–estimates were combined together using the Rubin's rules.[18,19]

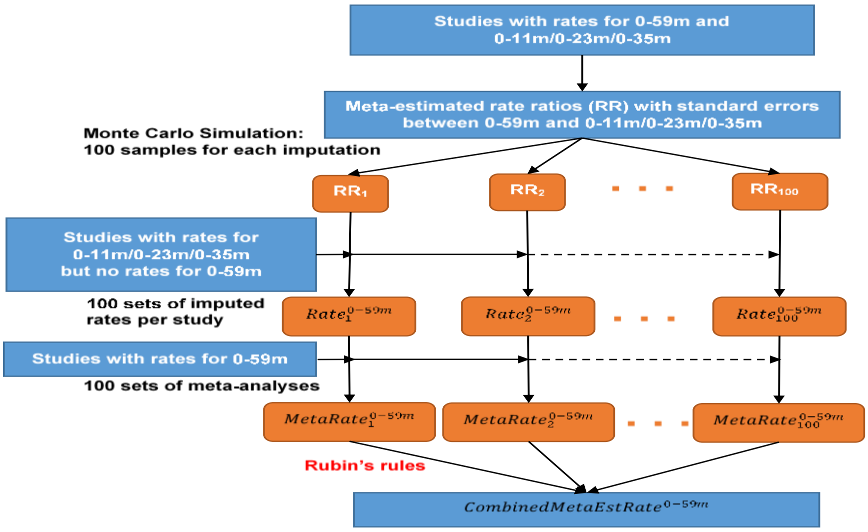

Figure S9.1. Imputing missing rates for 0–59 months using the multiple imputation approach.

Table S9.1. Pooled incidence rates of hPIV–associated ALRI for 0–59 months including and excluding imputed data*

	Pooled rates with imputed data		Pooled rates without imputed data	
	**No of studies (No. of imputed studies)**	**Rate**	**No of studies**	**Rate**
Low child mortality setting	4 (3)	37.8 (18.5-77.3)	1	18.8 (14.3-24.2)†
High child mortality setting	8 (3)	38.8 (30.1-50.2)	5	32.0 (25.6-39.9)‡

* Using multiple imputation method.
† Only one study reported data for 0-59 months in low child mortality settings. 95% confidence intervals were calculated based on the binomial distribution.
‡ This rate was mainly driven by three studies in Bangladesh (39.5 per 1,000 children per year for 0-59 m), South Africa (43.1 per 1,000 children per year for 0-59 m), and Nepal (48 per 1,000 children per year for 0-23 m) reporting high rates. The three studies had the largest sample sizes. After imputation, the rates for 0-59 m in the Nepali study were included in the meta-analysis.

Appendix 10. Adjusting for under-detection of hPIV

1. Details of denominator scaling in incidence rate and hospital admission rate data

Since not all ALRI cases were tested, we adjusted for the level of testing at study levels: we scaled the population-at-risk by applying the original denominator to the proportion of eligible cases who are tested (Formula 10.1). The scaled denominator could better reflect the true size of each study than the original denominator, thus the weight of each study in the meta-analysis.

Formula 10.1 - scaling the denominator:

$$\text{Rate} = \text{No. of observed cases}/(\text{Proportion of test} * \text{Original denominator})$$

2. hCFR data

For hCFR estimation, we only used the information from the cases that were tested, and did not adjust for the under-detection. The hCFR of tested ALRI cases was higher than the hCFR of untested ALRI cases (Table S10.1).

Table S10.1. The hCFR for tested ALRI cases and the hCFR for untested ALRI cases for 0-59 months using available data.*†

ID	Location	Year	Tested cases (A1)	Tested deaths (A2)	Untested cases (B1)	Untested deaths (B2)	hCFRs for tested (A=A2/A1, %)	hCFR for untested (C=B2/B1, %)
up_14	Kilifi, Kenya	2007-2011; 2013-2017	2757	93	1270	79	3.4	6.2
up_21	Basse, Gambia	2012-2013	623	17	12	5	2.7	41.7
up_22	Lusaka, Zambia	2011-2014	590	105	16	9	17.8	56.2
up_24	Soweto, South Africa	2011-2013	866	33	8	0	3.8	0
up_27	Kilifi, Kenya	2011-2013	566	27	2	2	4.8	100
up_28	Bamako, Mali	2012-2014	659	100	1	0	15.2	0
up_29	Rabat, Morocco	2010-2011	771	29	18	1	3.8	5.6
up_3	Buenos Aires, Argentina	2000-2017	12311	227	1626	25	1.8	1.5
up_30	Manhiça, Mozambique	2011-2014	478	12	14	2	2.5	14.3
up_38	Soweto, South Africa	1998-2005	2602	138	119	32	5.3	26.9
up_5	Klerksdorp, South Africa	2010-2015	1259	31	45	2	2.5	4.4
up_6	Pietermaritzburg, South Africa	2010-2015	2164	18	52	1	0.8	1.9
up_7	Colorado, United States of America	2010-2016	6424	60	9261	18	0.9	0.2
up_8	Berlin, Germany	2010-2014	2512	9	13	0	0.4	0
Meta-estimates							3.0 (1.7-5.0)	6.3 (1.9-18.8)

* Studies with small number of ALRI deaths (<5 ALRI deaths) were excluded in this analysis. The hCFRs in these studies were very imprecise. Also, very few hPIV-deaths would be missed due to under-detection.
† Studies were not excluded in this analysis if all ALRI cases were tested. Studies were excluded in this analysis if information of testing levels was unavailable.

Appendix 11. Assessment tool for risk of bias in individual studies

We used a modified Newcastle-Ottawa Scale when assessing risk of bias. Since our study does not include comparison groups, we removed the domains in the original scale that are related to comparison groups (e.g., case vs control; exposed vs non-exposed). The original scale includes domains related to biases in different aspects of one study, such as representativeness, case definition, and exposure ascertainment. We modified these domains to assess the biases specific to our study. For example, for the representativeness of study population in the original scale, we modified to “patient group excluded” and “adjustment for health utilization”. The adequacy of case definition in the original scale is maintained in the modified scale. For the non-response rate and loss to follow-up in the original scale, we modified it to “sampling strategy for detecting influenza”. For the ascertainment of the exposure in the original scale, we modify to “test method” for virus detection. Moreover, we add a category of “study design” as retrospective studies are usually susceptible to more biases and usually provide less information than prospective studies.

Table S11.1 Assessment of risk of biases for community-based studies

Category	Description	Risk of bias
Study design	Studies where the cases were prospectively enrolled	Low
	Other studies	High
Patient groups excluded	No exclusions that may affect estimates	Low
	Exclusions that may affect estimates, e.g., any of the following:<br>1. Not including very young children (e.g., neonates).<br>2. Excluding children with high-risk conditions.<br>3. Other exclusions that may affect estimates	High
Case definition	Using common/standard definitions	Low
	Using non-standard/inconsistent definitions	High
Sampling strategy	The proportion of testing is available AND either of the following:<br>1. >=90% of eligible cases have been tested.<br>2. Testing a systematic sample of patients.	Low
	<90% of eligible cases have been tested<br>OR<br>The proportion of eligible cases who have been tested is unavailable.	High
Diagnostic test	PCR;<br>Or using other diagnostic tests, but confirming negative samples with PCR	Low
	1. Other diagnostic tests, e.g., culture, IFA, DFA.<br>2. No mention of diagnostic tests	High

Table S11.2 Assessment of risk of biases for hospital-based studies

Category	Description	Risk of bias
Study design	Studies where the cases were prospectively enrolled	Low
	Other study designs	High
Adjustment for healthcare utilization (only for hospital admission rate studies)	Meeting either of the following:<br>1. Including all or main hospitals;<br>2. Not including main hospitals, but adjusting for the proportion of patients admitted in the study hospitals	Low
	Not including main hospitals OR no related description; AND no adjustment for the proportion of patients admitted in the study hospitals	High
Patient groups excluded	No exclusions that may affect estimates	Low
	Exclusions that may affect estimates, e.g., any of the following:<br>1. Not including very young children (e.g., neonates).<br>2. Excluding children with high-risk conditions.<br>3. Other exclusions that may affect estimates	High
Case definition	Using common/standard definitions	Low
	Using non-standard/inconsistent definitions	High
Sampling strategy	The proportion of testing is available AND either of the following:<br>1. >=90% of eligible patients have been tested.<br>2. Testing a systematic sample of patients.	Low
	<90% of eligible cases have been tested<br>OR<br>The proportion of eligible cases who have been tested is unavailable.	High
Diagnostic test (only for hospital admission rate studies)	PCR;<br>Or using other diagnostic tests, but confirming negative samples with PCR or culture	Low
	1. Other diagnostic tests, e.g., culture, IFA, DFA.<br>2. No mention of diagnostic tests	High
Hypoxaemia ascertainment (only for studies providing hypoxaemia data)	SpO2 was recorded for all metapneumovirus-confirmed cases	Low
	1. SpO2 was recorded for a proportion of metapneumovirus-confirmed cases.<br>2. No mention of how many metapneumovirus-confirmed cases have been assessed for hypoxaemia.	High

Appendix 12. Risk of bias in included studies

Table S12.1 Risk of bias for community-based studies reporting incidence rates of hPIV-associated ALRI (13 studies)

ID	Location; period	Study Design	Patient groups excluded	Case definition	Sampling strategy	Test method
442	India; 2001-2005	Low	Low	Low	High	High
742	USA; 2009	Low	Low	Low	High	Low
806	Bangladesh; 2004-2008	Low	Low	Low	Low	Low
up_2	India; Aug 2012-Aug 2014	Low	Low	Low	Low	Low
up_39	Pakistan; Dec 2012-Dec 2013	Low	Low	Low	High	Low
up_4	Bangladesh; 2013-2014	Low	Low	Low	High	Low
up_40	South Africa; 2012-2017	Low	Low	Low	Low	Low
up_41	Nepal; 2004-2007	Low	High	Low	Low	Low
388	USA; 1976-2001	Low	High	High	High	High
421	Bangladesh; 1993-1994	Low	Low	Low	High	High
735	Australia; 2010-2014	Low	High	Low	High	Low
737	Australia; 1996-1999	Low	High	Low	Low	Low
743	Spain; 1996-1999	Low	Low	High	High	High

Table S12.2 Risk of bias for community-based studies reporting incidence rates of hPIV-associated severe ALRI (5 studies).

ID	Location; period	Study Design	Patient groups excluded	Case definition	Sampling strategy	Test method
12	Pakistan; 2011-2014	Low	Low	Low	Low	Low
442	India; 2001-2005	Low	Low	Low	High	High
up_2	India; 2012-2014	Low	Low	Low	Low	Low
up_4	Bangladesh; 2013-2014	Low	Low	Low	High	Low
up_40	South Africa; 2012-2017	Low	Low	Low	Low	Low

Table S12.3 Risk of bias for hospital-based studies reporting hospital admission rate of hPIV-associated ALRI (38 studies)

ID	Location; period	Study Design	Adjustment for healthcare utilization	Patient groups excluded	Case definition	Sampling strategy	Test method
158	Thailand; 2005-2010	Low	Low	Low	High	Low	High
174	USA; 2010-2012	Low	Low	High	Low	Low	High
27	Nunavut; 1997-1998	Low	Low	Low	Low	High	High
386	USA; 2000-2004	Low	Low	High	High	Low	Low
51	India; 2009-2011	Low	Low	Low	High	Low	Low
6	Kenya; 2007-2010	Low	Low	Low	Low	Low	Low
649	Germany; 1996-2000	High	Low	Low	High	High	Low
742	USA; 2009	Low	Low	Low	Low	High	Low
78	China; 2003-2006	Low	Low	High	High	Low	High
815	the Gambia; 2015	Low	Low	High	Low	Low	Low
85	Spain; 2004-2007	Low	Low	Low	High	Low	Low
up_10	Philippines; 2014-2016	Low	Low	Low	Low	Low	Low
up_14	Kenya; 2007-2017	Low	Low	Low	Low	High	Low
up_15	Viet Nam; 2007-2016	Low	High	Low	Low	Low	Low
up_16	Argentina; Jun 2008-Dec 2010	Low	High	High	High	Low	High
up_17	Philippines; 2000-2004	Low	Low	High	Low	Low	Low
up_18	Jordan; Mar 2010-Mar 2013	Low	High	Low	Low	Low	Low
up_21	Gambia; 2012-2013	Low	High	High	Low	Low	Low
up_23	Thailand; Jan 2012-Dec 2013	Low	High	High	Low	Low	Low
up_25	Thailand; Jan 2012-Dec 2013	Low	High	High	Low	Low	Low
up_3	Argentina; 2000-2017	Low	High	Low	Low	Low	High
up_30	Mozambique; 2011-2014	Low	Low	Low	Low	Low	Low
up_31	Chile; 2012-2013	Low	Low	Low	Low	Low	High
up_32	Chile; 2012-2013	Low	Low	Low	Low	Low	High
up_38	South Africa; 1998-2005	Low	Low	High	Low	Low	High
up_4	Bangladesh; 2013-2014	Low	Low	Low	Low	High	Low
up_40	South Africa; 2012-2017	Low	Low	Low	Low	Low	Low
up_5	South Africa; 2010-2015	Low	High	Low	Low	Low	Low
up_6	South Africa; 2010-2015	Low	High	Low	Low	Low	Low
122	Germany; 1999-2001	Low	Low	Low	High	High	Low
176	China; 2007-2008	High	Low	Low	Low	High	High
253	Canada; 2007-2012	High	Low	Low	Low	High	High
569	Spain; 2011-2012	Low	Low	High	High	Low	Low
738	Mozambique; 2006-2007	Low	Low	Low	Low	Low	Low
739	Kenya; 2007-2010	Low	Low	Low	Low	High	Low
up_19	Bangladesh; 2010-2014	Low	Low	Low	High	Low	Low
865	Taiwan; 2010-2013	Low	Low	High	High	High	Low
891	Australia; 2019-2014	Low	Low	High	Low	High	Low

Table S12.4 Risk of bias for hospital-based studies reporting proportions of hPIV-associated ALRI (168 studies)

ID	Location; period	Study Design	Patient groups excluded	Case definition	Sampling strategy	Test method
104	Brazil; 2008-2010	Low	Low	High	Low	Low
110	France; 2002-2004	High	Low	Low	High	High
122	Germany; 1999-2001	Low	Low	High	High	Low
123	France; 2003-2004	Low	Low	High	High	High
131	Argentina; 1998-2002	High	Low	Low	High	High
151	Brazil; 2012-2013	Low	Low	Low	High	Low
158	Thailand; 2005-2010	Low	Low	High	Low	High
161	USA; 1996-1998	High	Low	High	High	High
167	France; 2007-2008	High	Low	High	High	Low
189	Cameroon; 2011-2013	Low	Low	High	Low	Low
190	Oman; 2007-2008	Low	High	High	Low	Low
191	Malaysia; 1992-2008	High	Low	High	High	High
206	Ghana; 2008	Low	Low	Low	Low	Low
223	China; 2011-2013	High	Low	High	Low	Low
229	China; 2013-2015	Low	Low	Low	Low	High
253	Canada; 2007-2012	High	Low	Low	High	High
284	Brazil; 2005-2007	Low	High	High	Low	High
291	Thailand; 2013-2014	Low	High	Low	High	Low
329	USA; 2005-2007	Low	Low	High	High	Low
340	Brazil; 1992	High	Low	High	High	High
349	China; 2001-2006	High	Low	Low	Low	High
355	Taiwan; 1997-1999	High	Low	High	Low	High
373	Argentina; 1998-2002	High	Low	Low	High	High
378	China; 2004-2012	High	Low	High	High	High
379	China; 2012-2015	Low	Low	High	Low	High
383	USA; 2010-2014	Low	Low	High	Low	Low
386	USA; 2000-2004	Low	High	High	Low	Low
391	Israel; 2001-2005	Low	Low	Low	Low	Low
401	China; 2010-2011	Low	Low	Low	Low	Low
410	Italy; 2004-2008	High	Low	Low	Low	Low
421	Bangladesh; 1993-1994	Low	Low	Low	High	High
428	Saudi Arabia; 1997-2001	Low	Low	High	High	High
436	Saudi Arabia; 1993-1996	High	Low	High	Low	High
443	Saudi Arabia; 2005-2010	High	Low	Low	Low	High
449	China; 2014	High	High	Low	Low	Low
463	Viet Nam; 2009-2010	Low	High	High	Low	Low
469	Republic of Korea; 2011-2012	High	Low	High	High	Low
488	Japan; 2007-2012	Low	Low	Low	High	Low
503	China; 2011	Low	Low	High	Low	Low
51	India; 2009-2011	Low	Low	High	Low	Low

ID	Location; period	Study Design	Patient groups excluded	Case definition	Sampling strategy	Test method
530	China; 2009-2014	Low	Low	High	High	Low
534	China; 2010-2012	High	Low	High	Low	Low
556	Australia; 2000-2005	High	Low	Low	Low	Low
566	Mexico; 2002-2004	Low	Low	High	Low	Low
57	Spain; 2005-2008	Low	Low	High	High	Low
570	China; 2007	High	Low	Low	Low	Low
573	Poland; 2008-2011	High	Low	High	High	Low
576	China; 2014	High	High	Low	Low	Low
593	Spain; 2011	Low	High	High	Low	High
604	India; 2011-2012	Low	Low	Low	Low	Low
605	Thailand; 1998-2001	Low	Low	High	High	High
613	Brazil; 1987-1989	Low	Low	Low	High	High
630	Paraguay; 2009	High	Low	Low	High	Low
642	China; 2001-2003	High	High	Low	Low	High
649	Germany; 1996-2000	High	Low	High	High	Low
660	China; 2007-2008	Low	Low	Low	High	Low
675	China; 2011-2012	Low	Low	Low	High	Low
695	Bangladesh; 2014-2015	Low	High	High	High	Low
7	Republic of Korea; 1996-1998	High	High	High	Low	High
730	Spain; 2011-2013	Low	Low	Low	High	Low
731	United Kingdom; 2009-2012	Low	High	Low	High	Low
733	Cyprus; 2010-2013	Low	Low	High	High	Low
736A	Egypt; 2007-2014	Low	Low	High	High	Low
736B	Jordan; 2008-2010	Low	Low	High	High	Low
736C	Oman; 2008-2009	Low	Low	High	High	Low
736D	Qatar; 2008-2009	Low	Low	High	High	Low
736E	Yemen; 2010-2014	Low	Low	High	High	Low
738	Mozambique; 2006-2007	Low	Low	Low	Low	Low
739	Kenya; 2007-2010	Low	Low	Low	High	Low
740	China; 2007-2010	Low	Low	High	Low	Low
741	Brazil; 2008-2009	Low	Low	High	Low	Low
742	USA; 2009	Low	Low	Low	High	Low
744	South Africa; 2003-2004	High	Low	High	High	High
745	China; 2010-2011	Low	Low	High	Low	Low
769	China; 2016-2017	Low	Low	High	Low	Low
785	China; 2012-2015	Low	Low	High	Low	Low
827	China; 2014-2016	Low	High	Low	Low	Low
831	China; 2008-2014	Low	Low	Low	High	Low
833	China; 2006-2015	Low	Low	High	High	High
838	China; 2017-2018	Low	Low	Low	Low	Low
841	China; 2014-2017	Low	Low	High	Low	Low
843	China; 2017	High	Low	High	High	High
847	China; 2017	High	Low	High	High	Low

ID	Location; period	Study Design	Patient groups excluded	Case definition	Sampling strategy	Test method
85	Spain; 2004-2007	Low	Low	High	Low	Low
c100	China; 2012-2013	Low	Low	Low	Low	Low
c112	China; 2003-2006	High	Low	High	Low	High
c12	China; 2011-2012	High	Low	Low	Low	High
c139	China; 2012-2013	High	Low	High	Low	High
c14	China; 2011-2012	High	Low	High	Low	Low
c143	China; 2014	High	Low	High	Low	High
c155	China; 2014-2015	High	Low	High	Low	High
c195	China; 2011-2013	High	Low	High	Low	High
c203	China; 2013-2014	High	Low	Low	Low	High
c213	China; 2011	High	Low	High	Low	High
c231	China; 2009-2012	High	Low	High	Low	High
c233	China; 2000	High	Low	Low	Low	High
c235	China; 2014-2015	High	Low	High	Low	High
c283	China; 2013-2014	Low	Low	High	Low	Low
c287	China; 2014	Low	High	Low	Low	High
c290	China; 2014-2015	High	High	High	Low	High
c293	China; 2015	High	Low	High	Low	Low
c294	China; 2015	High	Low	High	Low	High
c295	China; 2016	High	Low	High	Low	High
c296	China; 2013-2015	High	Low	High	Low	High
c297	China; 2015-2016	High	Low	High	Low	High
c299	China; 2009-2010	Low	Low	Low	Low	Low
c300	China; 2001-2002	Low	High	Low	Low	High
c301	China; 2006-2007	Low	High	Low	Low	High
c302	China; 2003-2006	Low	Low	High	Low	High
c303	China; 2012-2013	Low	Low	Low	Low	Low
c304	China; 2013	High	Low	Low	Low	High
c305	China; 2010-2011	Low	Low	High	Low	Low
c311	China; 1994-1997	High	Low	Low	Low	High
c312	China; 2007-2009	High	High	Low	High	High
c313	China; 1996-1997	Low	High	Low	Low	High
c314	China; 2005-2007	Low	Low	Low	Low	High
c315	China; 2001-2003	High	Low	Low	High	High
c36	China; 2013-2014	Low	Low	Low	Low	Low
c362	China; 2016-2017	Low	High	High	Low	Low
c44	China; 2009-2012	Low	Low	High	Low	Low
c47	China; 2010	High	Low	Low	Low	Low
c59	China; 2005-2006	High	Low	Low	Low	High
c61	China; 2013-2014	High	Low	High	Low	High
c83	China; 2006-2010	High	Low	Low	Low	High
c87	China; 2013-2014	Low	Low	Low	Low	High
c95	China; 2007	High	Low	Low	Low	High
c97	China; 2011-2012	High	Low	Low	Low	High
up_10	Philippines; 2014-2016	Low	Low	Low	Low	Low

ID	Location; period	Study Design	Patient groups excluded	Case definition	Sampling strategy	Test method
up_11	Philippines; Sep 2012-Jul 2016	Low	Low	Low	Low	Low
up_12	Philippines; Sep 2012-Feb 2015	Low	Low	Low	Low	Low
up_13	Philippines; Aug 2012-Feb 2015	Low	Low	Low	Low	Low
up_14	Kenya; 2007-2017	Low	Low	Low	High	Low
up_15	Viet Nam; 2007-2016	Low	Low	Low	Low	Low
up_16	Argentina; Jun 2008-Dec 2010	Low	High	High	Low	High
up_17	Philippines; 2000-2004	Low	High	Low	Low	Low
up_18	Jordan; Mar 2010-Mar 2013	Low	Low	Low	Low	Low
up_19	Bangladesh; 2010-2014	Low	Low	High	Low	Low
up_20	Bangladesh; Jan 2012 - Dec 2013	Low	High	Low	Low	Low
up_21	Gambia; 2012-2013	Low	High	Low	Low	Low
up_22	Zambia; Oct 2011 - Oct 2014	Low	High	Low	Low	Low
up_23	Thailand; Jan 2012-Dec 2013	Low	High	Low	Low	Low
up_24	South Africa; Aug 2011 - Aug 2013	Low	High	Low	Low	Low
up_25	Thailand; Jan 2012-Dec 2013	Low	High	Low	Low	Low
up_26	Bangladesh; Jan 2012 - Dec 2013	Low	High	Low	Low	Low
up_27	Kenya; Aug 2011 - Nov 2011	Low	High	Low	Low	Low
up_28	Mali; Jan 2012 - Jan 2014	Low	High	Low	Low	Low
up_29	Morocco; Nov 2010-Dec 2011	Low	High	Low	Low	Low
up_3	Argentina; 2000-2017	Low	Low	Low	Low	High
up_30	Mozambique; 2011-2014	Low	Low	Low	Low	Low
up_31	Chile; 2012-2013	Low	Low	Low	Low	High
up_32	Chile; 2012-2013	Low	Low	Low	Low	High
up_33	Iran (Islamic Republic of); 2008-2009	High	Low	High	High	Low
up_34	Iran (Islamic Republic of); 2017	High	Low	High	Low	Low
up_35	Iran (Islamic Republic of); Sep 2012- Sep 2013	High	Low	High	Low	Low
up_36	Iran (Islamic Republic of); Jan 2003 to Jan 2004	High	Low	High	Low	High
up_37	Iran (Islamic Republic of); Oct 1998-Oct 2000	High	Low	High	Low	Low
up_38	South Africa; 1998-2005	Low	High	Low	Low	High
up_4	Bangladesh; 2013-2014	Low	Low	Low	High	Low
up_40	South Africa; 2012-2017	Low	Low	Low	Low	Low
up_42	Nepal; Jan 2006-Jan 2008	Low	High	Low	Low	Low
up_5	South Africa; 2010-2015	Low	Low	Low	Low	Low
up_6	South Africa; 2010-2015	Low	Low	Low	Low	Low
up_8	Germany; Jan 2010-Dec 2014	Low	Low	Low	Low	Low

ID	Location; period	Study Design	Patient groups excluded	Case definition	Sampling strategy	Test method
up_9	Philippines; May 2008-Feb 2015	Low	Low	Low	Low	Low
865	Taiwan; 2010-2013	Low	High	High	High	Low
874	South Korea (Republic of Korea); 2010-2015	High	Low	Low	High	Low
875	China; 2017-2018	High	High	High	High	High
886	China; 2008-2017	High	Low	Low	High	High

Table S12.5. Risk of bias for hospital-based studies reporting in-hospital case-fatality ratios (hCFRs) of hPIV-associated ALRI (58 studies)

ID	Location; period	Study Design	Patient groups excluded	Case definition	Sampling strategy
104	Brazil; 2008-2010	Low	Low	High	Low
148	Cambodia; 2007-2010	Low	High	Low	High
263	Thailand; 2003-2007	Low	Low	High	High
284	Brazil; 2005-2007	Low	High	High	Low
329	USA; 2005-2007	Low	Low	High	High
368	Chile; 2001-2004	High	Low	Low	High
406	India; 2002-2004	Low	Low	High	Low
438	Mali; 2011-2012	Low	High	Low	Low
458	South Africa; 2009-2014	Low	Low	Low	Low
459	South Africa; 2010-2013	Low	Low	Low	Low
462	Viet Nam; 2004-2008	Low	High	High	Low
463	Viet Nam; 2009-2010	Low	High	High	Low
480	Spain; 1994-2000	Low	Low	High	High
492	Turkey; 2006-2007	Low	High	High	Low
51	India; 2009-2011	Low	Low	High	Low
519	Republic of Korea; 1994-1998	High	Low	High	High
64	Argentina; 1993-1994	Low	High	Low	Low
696	Tunisia; 2013-2014	High	High	High	High
736A	Egypt; 2007-2014	Low	Low	High	High
736B	Jordan; 2008-2010	Low	Low	High	High
736C	Oman; 2008-2009	Low	Low	High	High
736D	Qatar; 2008-2009	Low	Low	High	High
736E	Yemen; 2010-2014	Low	Low	High	High
738	Mozambique; 2006-2007	Low	Low	Low	Low
78	China; 2003-2006	Low	High	High	Low
801	Brazil; 1990-2017	Low	Low	High	Low
c310	China; 2009-2010	Low	High	High	Low
up_10	Philippines; 2014-2016	Low	Low	Low	Low
up_11	Philippines; Sep 2012-Jul 2016	Low	Low	Low	Low
up_12	Philippines; Sep 2012-Feb 2015	Low	Low	Low	Low
up_13	Philippines; Aug 2012-Feb 2015	Low	Low	Low	Low
up_14	Kenya; 2007-2017	Low	Low	Low	High
up_16	Argentina; Jun 2008-Dec 2010	Low	High	High	Low
up_17	Philippines; 2000-2004	Low	High	Low	Low
up_18	Jordan; Mar 2010-Mar 2013	Low	Low	Low	Low
up_19	Bangladesh; 2010-2014	Low	Low	High	Low
up_20	Bangladesh; Jan 2012 - Dec 2013	Low	High	Low	Low
up_21	Gambia; 2012-2013	Low	High	Low	Low
up_22	Zambia; Oct 2011 - Oct 2014	Low	High	Low	Low
up_23	Thailand; Jan 2012-Dec 2013	Low	High	Low	Low
up_24	South Africa; Aug 2011 - Aug 2013	Low	High	Low	Low
up_25	Thailand; Jan 2012-Dec 2013	Low	High	Low	Low

up_26	Bangladesh; Jan 2012 - Dec 2013	Low	High	Low	Low
up_27	Kenya; Aug 2011 - Nov 2011	Low	High	Low	Low
up_28	Mali; Jan 2012 - Jan 2014	Low	High	Low	Low
up_29	Morocco; Nov 2010-Dec 2011	Low	High	Low	Low
up_3	Argentina; 2000-2017	Low	Low	Low	Low
up_30	Mozambique; 2011-2014	Low	Low	Low	Low
up_31	Chile; 2012-2013	Low	Low	Low	Low
up_32	Chile; 2012-2013	Low	Low	Low	Low
up_38	South Africa; 1998-2005	Low	High	Low	Low
up_40	South Africa; 2012-2017	Low	Low	Low	Low
up_5	South Africa; 2010-2015	Low	Low	Low	Low
up_6	South Africa; 2010-2015	Low	Low	Low	Low
up_7	USA; 2010-2016	Low	Low	Low	High
up_8	Germany; Jan 2010-Dec 2014	Low	Low	Low	Low
up_9	Philippines; May 2008-Feb 2015	Low	Low	Low	Low
861	Jordan; Jan-Apr 2016	Low	Low	High	Low

Appendix 13. Details of individual studies

Table S13.1 Glossary of abbreviations used in this section

**Abbreviation**	**Full name**
**ALRI**	acute lower respiratory infection
**APAAP**	alkaline phosphatase and monoclonal anti–alkaline phosphatase
**ARI**	acute respiratory infection
**BAL**	bronchoalveolar lavage
**DFA**	Direct fluorescent antibody test
**EIA**	enzyme immunoassay
**ELISA**	enzyme-linked immunosorbent assay
**hPIV**	human parainfluenza virus
**IFA**	indirect immunofluorescence assay
**m**	month(s)
**NA**	Not applicable
**NPA**	nasopharyngeal aspirate
**NPS**	nasopharyngeal swab
**NPW**	nasopharyngeal wash
**NS**	nasal swab
**NW**	Nasal wash
**OP specimen**	oropharyngeal specimen
**OPS**	oropharyngeal swab
**PCR**	polymerase chain reaction
**TS**	throat swab

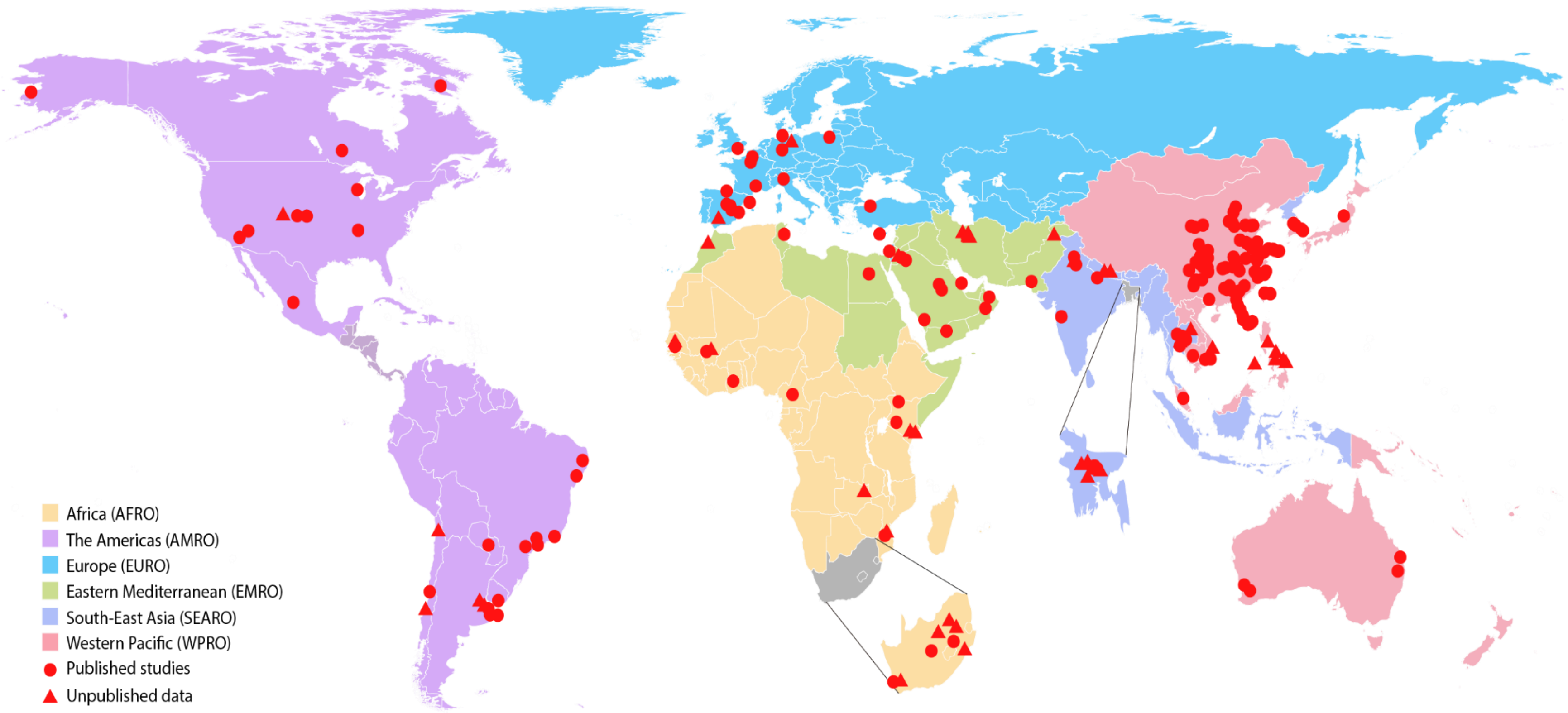

Figure S13.1. Location of included studies with data on human parainfluenza virus-associated incidence rates, hospital admission rates, proportion, and in-hospital case-fatality ratios.

Study	Case	Rate/1,000 and 95%CI
Jordan; Mar 2010-Mar 2013	51	0.8 (0.6-1.1)
Argentina; Jun 2008-Dec 2010	1	3 (0.4-21.3)
USA; 2000-2004	66	3.4 (2.7-4.4)
Thailand; 2005-2010	28	4 (2.8-5.8)
Spain; 2004-2007	26	4.5 (3.1-6.7)
Nunavut; 1997-1998	1	8.9 (1.3-60.6)
China; 2003-2006	14	12.5 (7.4-21.1)
Overall (I2=93.6%; P=0.000)		3.6 (1.8-7)

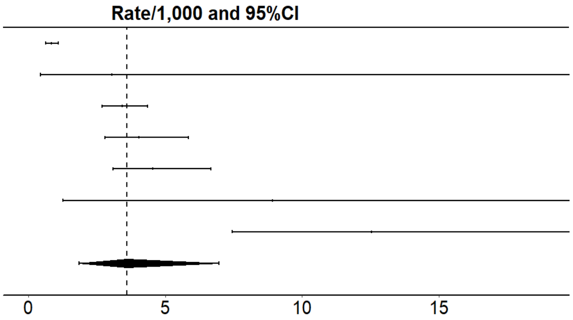

Study	Case	Rate/1,000 and 95%CI
Bangladesh; 2013-2014	1	0.8 (0.1-5.4)
Mozambique; 2011-2014	16	3.5 (2.1-5.7)
South Africa; 2010-2015	67	3.7 (2.9-4.6)
South Africa; 2010-2015	42	3.9 (2.9-5.3)
Kenya; 2007-2017	106	5 (4.1-6)
South Africa; 1998-2005	40	5.7 (4.2-7.7)
Gambia; 2012-2013	50	7.6 (5.8-10)
Philippines; 2014-2016	3	9.1 (2.9-27.8)
Philippines; 2000-2004	35	11.9 (8.5-16.5)
South Africa; 2012-2017	15	30.1 (18.2-49.3)
Overall (I2=94.3%; P=0.000)		5.8 (3.7-9.2)

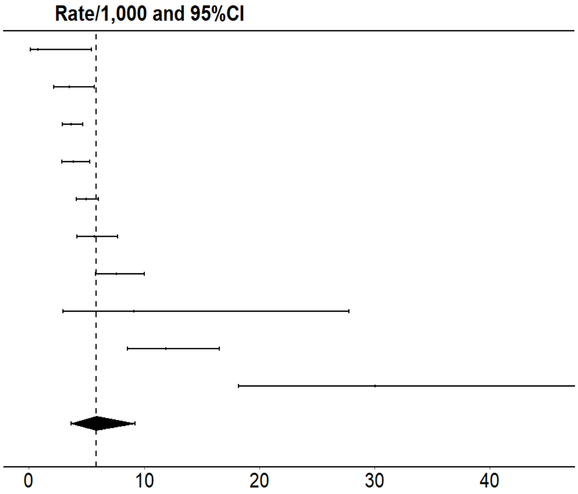

Figure S13.2 Forest plot of hospital admission rates of hPIV-ALRI for age 0-5 months for low child mortality settings (above) and high child mortality settings (below)

Study	Case	Rate/1,000 and 95%CI
Jordan; Mar 2010-Mar 2013	35	0.6 (0.4-0.8)
Argentina; Jun 2008-Dec 2010	1	1 (0.1-7.2)
USA; 2000-2004	39	2 (1.5-2.7)
Spain; 2004-2007	20	3.6 (2.3-5.5)
China; 2003-2006	9	8.1 (4.2-15.4)
Overall (I2=93.4%; P=0.000)		2 (0.9-4.6)

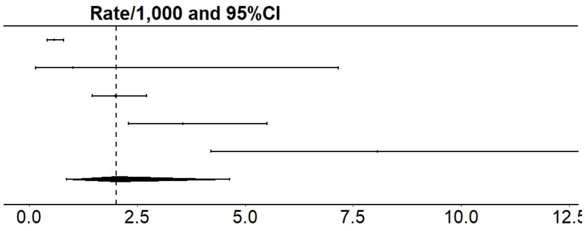

Study	Case	Rate/1,000 and 95%CI
Mozambique; 2011-2014	11	2.3 (1.3-4.2)
South Africa; 2010-2015	48	2.6 (2-3.5)
South Africa; 2010-2015	35	3.2 (2.3-4.4)
South Africa; 1998-2005	36	3.8 (2.7-5.2)
Bangladesh; 2013-2014	2	3.8 (1-15.1)
Kenya; 2007-2017	63	3.9 (3-5)
Gambia; 2012-2013	43	5.8 (4.3-7.8)
Philippines; 2000-2004	35	7.8 (5.6-10.8)
Philippines; 2014-2016	5	10.9 (4.6-26)
South Africa; 2012-2017	10	18.1 (9.8-33.4)
Overall (I2=87.8%; P=0.000)		4.7 (3.2-6.7)

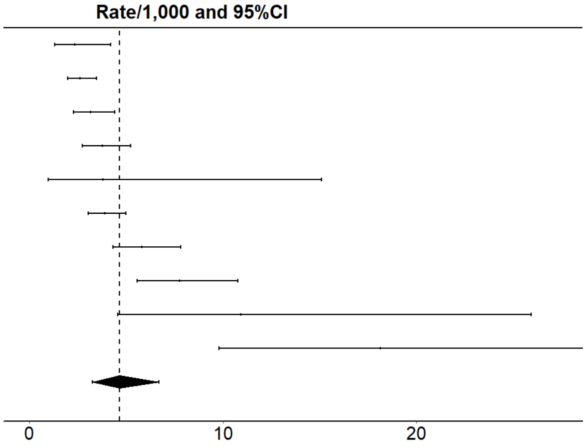

Figure S13.3 Forest plot of hospital admission rates of hPIV-ALRI for age 6-11 months for low child mortality settings (above) and high child mortality settings (below).

Study	Case	Rate/1,000 and 95%CI
Thailand; Jan 2012-Dec 2013	2	0.2 (0-0.8)
Chile; 2012-2013	18	0.3 (0.2-0.4)
Thailand; Jan 2012-Dec 2013	3	0.4 (0.1-1.1)
Chile; 2012-2013	15	0.4 (0.2-0.6)
Germany; 1996-2000	11	0.7 (0.4-1.2)
Argentina; 2000-2017	125	2.2 (1.9-2.6)
Argentina; Jun 2008-Dec 2010	5	2.7 (1.1-6.4)
China; 2003-2006	61	6.8 (5.3-8.7)
Overall (I2=96.9%; P=0.000)		0.8 (0.3-1.9)

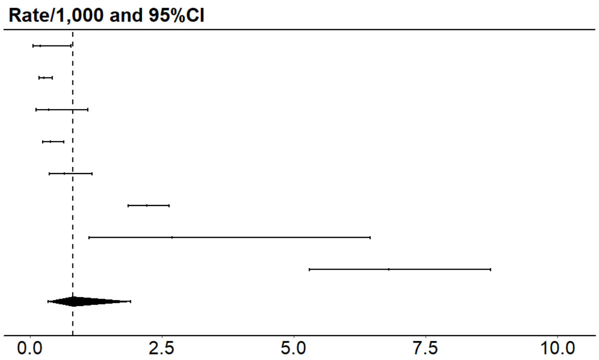

Study	Case	Rate/1,000 and 95%CI
Bangladesh; 2013-2014	0	0 (0-1000)
South Africa; 1998-2005	24	0.3 (0.2-0.5)
Mozambique; 2011-2014	14	0.4 (0.2-0.6)
South Africa; 2010-2015	68	0.5 (0.4-0.6)
India; 2009-2011	6	0.5 (0.2-1.2)
Viet Nam; 2007-2016	72	0.6 (0.5-0.8)
South Africa; 2010-2015	56	0.7 (0.5-0.9)
Kenya; 2007-2017	92	0.7 (0.6-0.9)
Gambia; 2012-2013	39	0.8 (0.6-1)
Philippines; 2014-2016	4	0.9 (0.4-2.5)
South Africa; 2012-2017	10	3.7 (2-6.9)
Kenya; 2007-2010	225	4.4 (3.9-5)
Overall (I2=96.6%; P=0.000)		0.7 (0.5-1.2)

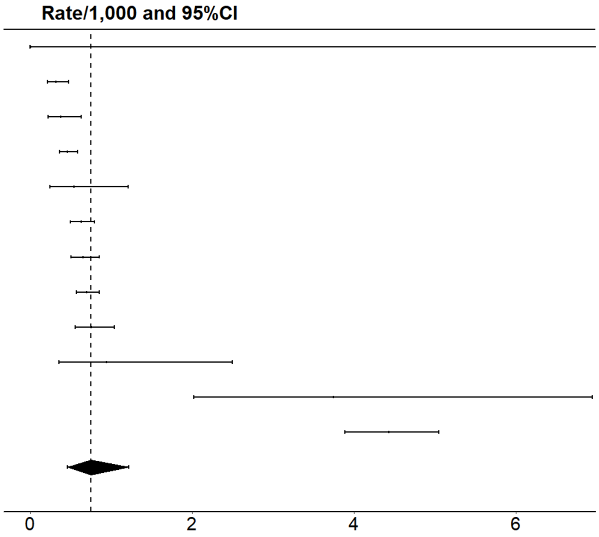

Figure S13.4 Forest plot of hospital admission rates of hPIV-ALRI for age 12-59 months for low child mortality settings (above) and high child mortality settings (below).

Study	Case	Rate/1,000 and 95%CI
Pakistan; Dec 2012-Dec 2013	10	17.3 (9.3-31.8)
Bangladesh; 2004-2008	174	27.4 (23.7-31.8)
India; Aug 2012-Aug 2014	84	29.4 (23.8-36.3)
Bangladesh; 2013-2014	151	39.5 (33.7-46.1)
South Africa; 2012-2017	160	43.1 (37-50.1)
Overall (I2=83.8%; P=0.000)		32 (25.6-39.9)

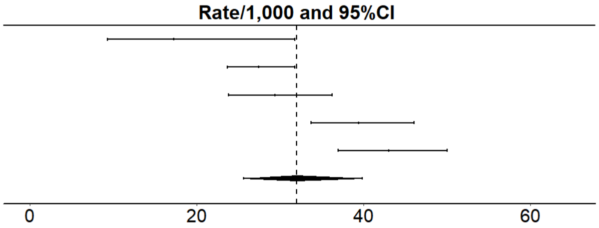

Figure S13.5 Forest plot of community incidence rates of hPIV-ALRI for age 0-59 months in high child mortality settings. Note: the pooled estimate differed from that in main Table 1 as only the 5 non-imputed studies were included here. The remaining 3 studies were imputed using a multiple imputation method, so were not presented in this figure.

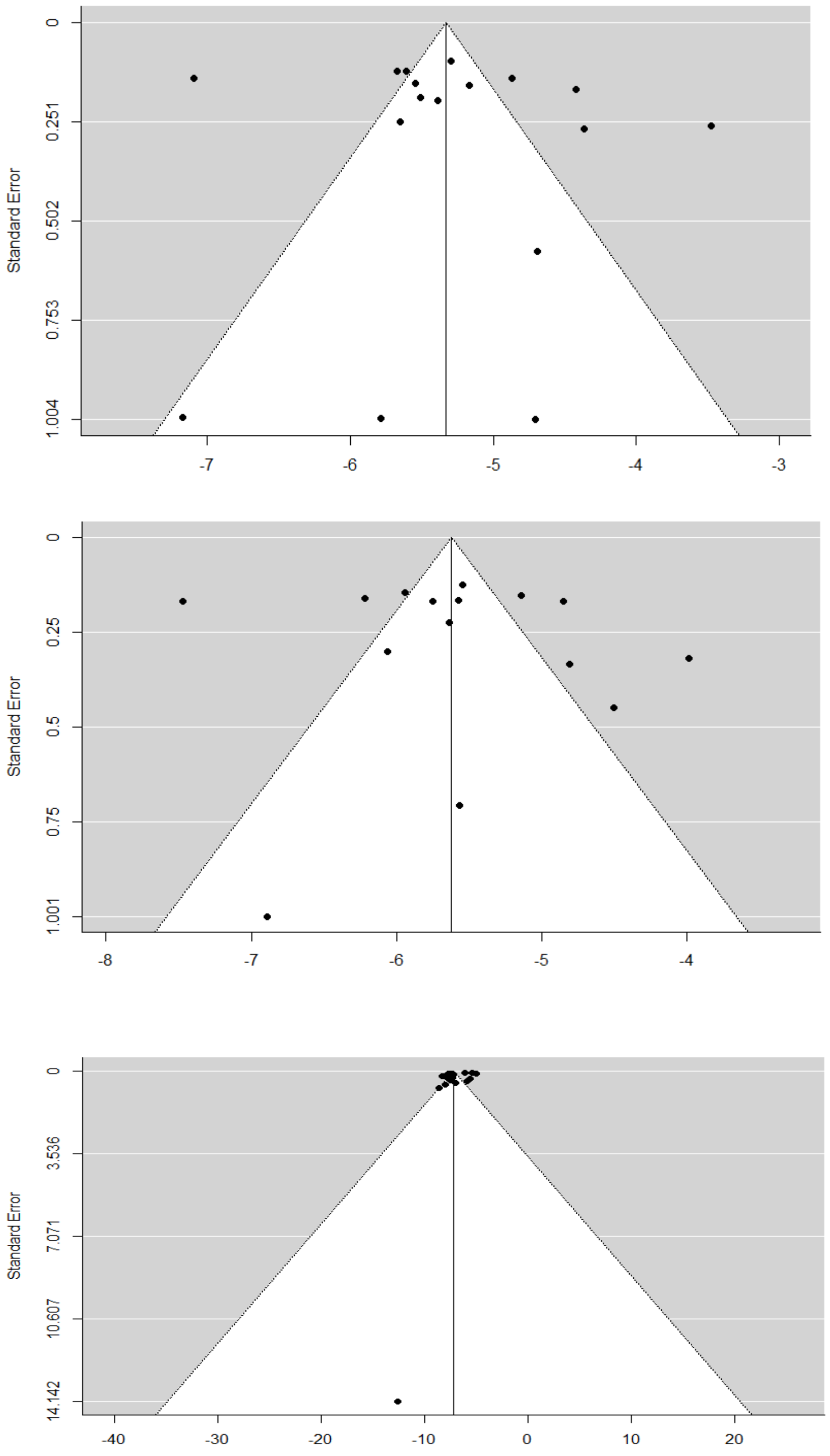

Figure S13.6 Funnel plots for hospital admission rates of hPIV-ALRI for ages 0-5 months (above), 6-11 months (middle) and 12-59 months (below).

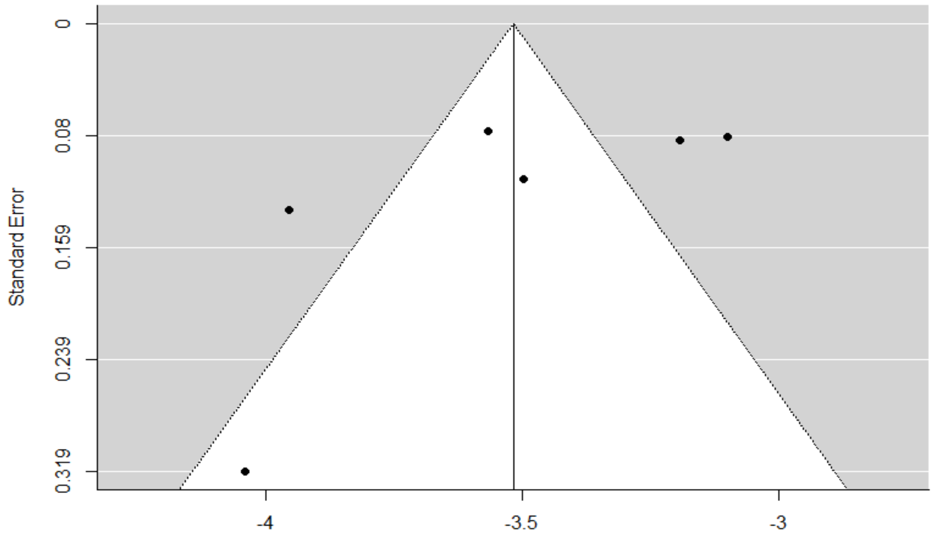

Figure S13.7 Funnel plot of community incidence rates of hPIV-ALRI for 0-59 months. Note: only non-imputed studies were presented in this plot. The remaining 4 studies were imputed using a multiple imputation method, and were not presented in this plot.

Table S13.2. Description of included studies reporting incidence rates of hPIV-associated ALRI cases in children younger than five years (13 studies)*†

				Incidence rates (per 1,000 children per year)					
**Location (reference)**	**Case Definition**	**Denominator type**	**Specimen and test**	**0–5 m**	**6–11 m**	**12–23 m**	**24–59 m**	**0–59 m**	**Published references (for unpublished data)**
**Ballabgarh, India (2001–2005) (Broor et al. 2007)** [20]	ALRI	Defined population base	NPA; DFA	··	··	80.4	··	··	··
**Navajo and White Mountain Apache, USA (2009) (Bhat et al. 2013)** [21]	ALRI	Defined population base	Nasal wash; PCR	··	··	··	··	··	··
**Faridabad, India (Aug 2012–Aug 2014) (Krishnan and colleagues, unpublished)**	ALRI	Census derived estimate	OP and nasal specimens; PCR	45.5	90	46.2	11.9	29.4	[22]
**Oshikhandass, Pakistan (Dec 2012–Dec 2013) (Rasmussen and colleagues, unpublished)**	ALRI	Defined population base	NPS; PCR	0	15.9	46.2	9.2	17.3	[23]
**Kamalapur, Bangladesh (2013–2014) (Brooks and colleagues, unpublished)**	ALRI	Defined population base	NPW; PCR	11.5	59.8	92.1	45.2	39.5	[24]
**Paarl, South Africa (Jun 2012–Dec 2017) (Zar and colleagues, unpublished)**	ALRI	Defined population base	NPS; PCR	91.6	70.4	52.8	18	43.1	[25]
**Bhaktapur, Nepal (2004–2007) (Strand and colleagues, unpublished)**	ALRI	Defined population base	NPA; PCR	64.3	81.4	53.6	··	··	[26]
**Nashville, USA (1976–2001) (Williams et al. 2004)**[27]	ALRI; croup	Defined population base	NW; Culture	··	··	··	··	18.8	··
**Mirzapur, Bangladesh (1993–1994)**[28]	ALRI	Defined population base	NPA; ELISA	··	··	··	··	··	··
**Brisbane, Australia (2010–2014) (Sarna et al. 2018)**[29]	ALRI	Defined population base	NS; PCR	··	··	··	··	··	··
**Perth, Australia (1996–1999) (Kusel et al. 2006)**[30]	ALRI	Defined population base	NPA; PCR	··	··	··	··	··	··
**Barcelona, Spain (1996–1999) (Puig et al. 2008)**	ALRI; croup	Defined population base	NPA; Culture with IFA	··	··	··	··	··	··
**Kamalapur, Dhaka, Bangladesh (2004-2008) (Havers, et al. 2019)** [31]	ALRI	Defined population base	NPW; PCR	248.7	123.0	64.8	7.3	27.4	··

* ALRI: acute lower respiratory infections according to 2005 WHO IMCI. NP specimens: nasopharyngeal specimens. OP specimens: oropharyngeal specimens. NS: nasal swab. TS: throat swab. NPA: nasopharyngeal aspirate. NPS: nasopharyngeal swab. NPW: nasopharyngeal wash. NW: nasal wash. IFA: indirect immunofluorescence assay. DFA: direct immunofluorescence assay. PCR: polymerase chain reaction.

† Some of the studies had no data presented as they reported data for other age bands (eg, 0-23 months).

Table S13.3. Description of included studies reporting incidence rates of hPIV-associated severe ALRI cases in children younger than five years (5 studies)*

Location (reference)	Case Definition	Denominator type	Specimen and test	Incidence rates per 1,000 children per year					Published references (for unpublished data)
				0–5 m	6–11 m	12–23 m	24–59 m	0–59 m	
**Karachi, Pakistan (2011–2014) (Ali et al. 2016)[32]**	sALRI	Defined population base	NPS; PCR	72.7	··	··	··	··	··
**Ballabgarh, India (2001–2005) (Broor et al. 2007)[20]**	sALRI	Defined population base	NPA; DFA	··	··	8.7	··	··	··
**Faridabad, India (Aug 2012–Aug 2014) (Krishnan and colleagues, unpublished)**	sALRI	Census derived estimate	OP and nasal specimens; PCR	25.3	58.8	29.7	9.1	19.6	[22]
**Kamalapur, Bangladesh (2013–2014) (Brooks and colleagues, unpublished)**	sALRI	Defined population base	NPW; PCR	0.6	5.4	0	2.2	1.6	[24]
**Paarl, South Africa (Jun 2012–Dec 2017) (Zar and colleagues, unpublished)**	sALRI	Defined population base	NPS; PCR	76.9	58	29.9	10.2	30.4	[25]

* sALRI: severe acute lower respiratory infections (with chest wall indrawing and danger signs) according to 2005 WHO IMCI. OP specimens: oropharyngeal specimens. NPA: nasopharyngeal aspirate. NPS: nasopharyngeal swab. NPW: nasopharyngeal wash. DFA: direct immunofluorescence assay. PCR: polymerase chain reaction.

Table S13.4. Description of included studies reporting hospital admission rates of hPIV-associated ALRI cases in children younger than five years (38 studies)*

Location (reference)	Case Definition	Denominator type	Specimen and test	Hospital admission rates per 1,000 children per year					Published references (for unpublished data)
				0–5 m	6–11 m	12–23 m	24–59 m	0–59 m	
**Sa Kaeo and Nakhon Phanom, Thailand (2005–2010) (Hasan et al. 2014)[33]**	ARI	Census derived estimate	NPS and serum specimens; PCR, serologic test and culture	3.6	··	··	3.2	5.5	··
**Memphis, Nashville, and Salt Lake City (EPIC), USA (2010–2012) (Jain et al. 2015)[34]**	ALRI	Census derived estimate	NPS and OPS; PCR and serologic test	··	··	··	0.2	0.2	··
**Iqaluit, Nunavut (1997–1998) (Banerji et al. 2001) [35]**	ALRI	Census derived estimate	NPA; IFA	8.9	··	··	··	··	··
**NVSN sites, USA (2000–2004) (Weinberg et al. 2009) [36]**	Fever; ARI	Census derived estimate	NS and TS; PCR	3	1.7	1.5	0.4	1	··
**Haryana, India (2009–2011) (Broor et al. 2014)[37]**	ARI	Census derived estimate	NS and TS; PCR	··	··	··	··	0.7	··
**Kakuma and Dadaab, Kenya (2007–2010) (Ahmed et al. 2012)[38]**	ALRI	Census derived estimate	NPS and OPS; PCR	··	··	··	··	6.2	··
**Kiel, Germany (1996–2000) (Weigl et al. 2005)[39]**	ALRI; croup	Census derived estimate	NPA; PCR	··	··	··	··	0.5	··
**Navajo and White Mountain Apache, USA (2009) (Bhat et al. 2013) [21]**	ALRI	Defined population base	NW; PCR	··	··	··	··	··	··
**PYNEH and QMH, Hong Kong (2003–2006) (Chiu et al. 2010)[40]**	ARI–Fever	Census derived estimate	NPA; DFA and culture	10.8	7.2	9.9	4.8	6.6	··
**Gipuzkoa, Spain (2004–2007) (Cilla et al. 2009)[41]**	ARI; ARI–Fever	Census derived estimate	NPA; PCR and culture	4	3.2	1.2	··	··	··
**Kawayan and Caibiran, Philippines (2014–2016) (Oshitani and colleagues, unpublished**	ALRI	Defined population base	NPS; PCR	9.1	10.9	0	1.2	2.4	[42]
**Kilifi, Kenya (2007–2017) (Nokes and colleagues, unpublished)**	ALRI	Census derived estimate	NPS; PCR	5	3.9	1.6	0.4	1.5	[43]
**Nha Trang city, Viet Nam (2007–2016) (Yoshida and colleagues, unpublished)**	ALRI	Census derived estimate	NPS; PCR	··	··	1.6	0.2	0.8	[44]
**Buenos Aires, Argentina (Jun 2008–Dec 2010) (Echavarria and colleagues, unpublished)**	ARI	Defined population base	NPA; IFA	3	1	··	··	2.2	[45]
**multiple areas, Philippines (Jul 2000–Dec 2004) (Lucero and colleagues, unpublished)**	ALRI	Defined population base	Blood, NPS and NPA; serum and culture	11.9	7.8	2.3	··	··	[46]

* ARI: acute respiratory infections requiring hospital admission. ALRI: physician diagnosed acute lower respiratory infections requiring hospital admission. ARI-Fever: hospitalised acute respiratory infections with fever. NS: nasal swab. TS: throat swab. NW: nasal wash. PCR: polymerase chain reaction. NPA: nasopharyngeal aspirate. NPS: nasopharyngeal swab. OPS: oropharyngeal swab. NPW: nasopharyngeal wash. IFA: indirect immunofluorescence assay. DFA: direct immunofluorescence assay. IF: immunofluorescence.

Location (reference)	Case Definition	Denominator type	Specimen and test	Hospital admission rates per 1,000 children per year					Published references (for unpublished data)
				0–5 m	6–11 m	12–23 m	24–59 m	0–59 m	
**Amman, Jordan (Mar 2010–Mar 2013) (Khuri-Bulos and colleagues, unpublished)**	ALRI	Census derived estimate	NS and TS; PCR	0.7	0.5	0.3	··	··	[47]
**Basse, Gambia (2012–2013) (Deloria-Knoll and colleagues, unpublished)**	ALRI	Census derived estimate	NPS/OPS, Induced sputum; PCR	7.6	5.8	1.6	0.5	2	[11]
**Nakhon Phanom, Thailand (Jan 2012–Dec 2013) (Deloria-Knoll and colleagues, unpublished)**	ALRI	Census derived estimate	NP/OP and induced sputum; PCR	··	··	0.3	0.1	0.3	[11]
**Sa Kaeo, Thailand (Jan 2012–Dec 2013) (Deloria-Knoll and colleagues, unpublished)**	ALRI	Census derived estimate	NP/OP and induced sputum; PCR	··	··	0.7	0.1	0.5	[11]
**Buenos Aires, Argentina (2000–2017) (Gentile and colleagues, unpublished)**	ALRI	Defined population base	NPA; IFA	··	··	3.5	1.1	4.4	[48]
**Manhiça, Mozambique (Jan 2011–Jul 2014) (Bassat and colleagues, unpublished)**	ALRI	Defined population base	NPA; PCR	3.5	2.3	0.8	0.2	0.9	[11]
**Iquique, Chile (2012–2013) (Fasce and colleagues, unpublished)**	ALRI	Censue derived estimate	NPA; IF	··	··	1	0.2	0.7	[49]
**Concepcion, Chile (2012–2013) (Fasce and colleagues, unpublished)**	ALRI	Censue derived estimate	NPA; IF	··	··	0.4	0.2	0.4	[49]
**Soweto, South Africa (Mar 1998–Oct 2005) (Madhi and colleagues, unpublished)**	ALRI	Defined population base	NPA; IFA	5	3.3	0.9	0.1	1	[50]
**Kamalapur, Bangladesh (2013–2014) (Brooks and colleagues, unpublished)**	ALRI	Defined population base	NPW; PCR	0.8	3.8	0	0	0.9	[24]
**Paarl, South Africa (Jun 2012–Dec 2017) (Zar and colleagues, unpublished)**	ALRI	Defined population base	NPS; PCR	26.1	16.3	4.2	3.1	8.5	[25]
**Klerksdorp, South Africa (2010–2015) (Cohen and colleagues, unpublished)**	ALRI	Census derived estimate	NPA; PCR	3.4	2.8	1.4	0.3	1.1	[51]
**Pietermaritzburg, South Africa (2010–2015) (Cohen and colleagues, unpublished)**	ALRI	Census derived estimate	NPA; PCR	3.2	2.3	0.9	0.2	0.9	[51]
**Hamburg, Dresden, Freiburg, Bochum, Germany (1999–2001) (Forster et al. 2004)[52]**	ALRI; croup	Defined population base	NP secretion; PCR	··	··	··	··	··	··
**Suzhou, China (2007–2008) (Ji et al. 2010)[53]**	ALRI	Census derived estimate	nasal aspirate; DFA	··	··	··	··	0.6	··
**Sioux Lookout, Ontario, Canada (2007–2012) (McCuskee et al. 2014) [54]**	ALRI	Census derived estimate	··; immunochromatography assay and culture (all tested by culture)	··	··	··	··	··	··
**Severo Ochoa Hospital, Madrid, Spain (2011–2012) (Olabarrieta et al. 2015) [55]**	ALRI	Defined population base	NPA; PCR	··	··	··	··	··	··
**Manhica, Mozambique (2006–2007) (O'Callaghan-Gordo et al. 2011)[56]**	ALRI	Census derived estimate	NPA; PCR	··	··	··	··	1.5	··

Location (reference)	Case Definition	Denominator type	Specimen and test	Hospital admission rates per 1,000 children per year					
				0–5 m	6–11 m	12–23 m	24–59 m	0–59 m	Published references (for unpublished data)
**Asembo, Kenya (2007–2010) (Feikin et al. 2013)[57]**	ALRI	Census derived estimate	NPS or OPS; PCR	··	··	··	··	30	··
**multiple areas, Bangladesh (2010–2014) (Homaira and colleagues, unpublished)**	ARI–Fever; ALRI	Census derived estimate	NS and TS; PCR	··	··	··	··	1.4	[58]
**Basse, the Gambia (2015) (Mackenzie, et al. 2019) [59]**	ALRI	Census derived estimate	NPS and OPS; PCR	··	··	9.0	··	··	··
**Taiwan (2010-2013) (Chi et al. 2020)[60]**	Radiologically confirmed; ALRI	Census derived estimate	NPS; PCR	··	··	··	0.09	0.06	··
**Brisbane, Australia (2010-2014) (Saha et al, 2020)[61]**	ALRI	Defined population base	NS; PCR	··	··	··	··	··	··

Table S13.5. Description of included studies reporting hCFRs (%) of hPIV-associated ALRI cases in children younger than five years (58 studies)*†

Location (reference)	Case definition	Specimen and test	0 –5 m		6–11 m		12–59 m		0–59 m		
			Cases (No.)	hCFR (%)	Cases (No.)	hCFR (%)	Cases (No.)	hCFR (%)	Cases (No.)	hCFR (%)	Published references (for unpublished data)
**São Paulo city, Brazil (2008–2010) (Durigon et al. 2015)[62]**	ARI	NPA; PCR	··	··	··	··	··	··	··	··	··
**Takeo Province and Kampong Cham Province, Cambodia (2007–2010) (Guerrier et al. 2013)[63]**	ALRI	NPA; PCR	··	··	··	··	··	··	51	0	··
**Sa kaeo, Nakhon Phanom, Thailand (2003–2007) (Morgan et al. 2013)[64]**	ARI	NPS and serum specimen; PCR, serologic test and culture	··	··	··	··	··	··	370	0	··
**São Paulo city, Brazil (2005–2007) (Pecchini et al. 2015)[65]**	ARI–Fever; ARI	NP secretion; IFA	··	··	··	··	··	··	45	8.9	··
**Yukon Kuskokwim Delta, USA (2005–2007) (Singleton et al. 2010)[66]**	ALRI	NP specimens; PCR	··	··	··	··	··	··	··	··	··
**Santiago, Chile (2001–2004) (Vega-Briceño et al. 2007)[67]**	ALRI	NPS; DFA	··	··	··	··	··	··	··	··	··
**Pune, India (2002–2004) (Yeolekar et al. 2008)[68]**	ARI	NPA; IFA	··	··	··	··	··	··	··	··	··
**Bamako, Mali (2011–2012) (Benet et al. 2015)[69]**	ALRI	NS; PCR	··	··	··	··	··	··	13	15.4	··
**multi sites, South Africa (2009–2014) (Cohen et al. 2015)[70]**	ALRI	NPA; PCR	··	··	··	··	··	··	952	1.6	··
**multi sites, South Africa (2010–2013) (Cohen et al. 2016)[71]**	ALRI	NPA; PCR	226	2.7	··	··	··	··	··	··	··
**Ho Chi Minh City, Vietnam (2004–2008) (Do et al. 2011)[72]**	ARI	NS, TS, and NPA; PCR	··	··	··	··	··	··	19	0	··
**Ho Chi Minh City, Vietnam (2009–2010) (Do et al. 2016)[73]**	ARI	NPS; PCR	··	··	··	··	··	··	··	··	··
**Madrid, Spain (1994–2000) (García García et al. 2001)[74]**	ALRI; croup	NP secretions; IFA	··	··	··	··	··	··	··	··	··

* ARI: hospitalised acute respiratory infections. ALRI: physician diagnosed acute lower respiratory infections requiring hospital admission. ARI-Fever: hospitalised acute respiratory infections with fever. NS: nasal swab. TS: throat swab. NW: nasal wash. PCR: polymerase chain reaction. NPA: nasopharyngeal aspirate. NPS: nasopharyngeal swab. OPS: oropharyngeal swab. NPW: nasopharyngeal wash. IFA: indirect immunofluorescence assay. DFA: direct immunofluorescence assay. IF: immunofluorescence. EIA: enzyme immunoassay. ELISA: enzyme-linked immunosorbent assay. BAL: bronchoalveolar lavage. APAAP: alkaline phosphatase and monoclonal anti-alkaline phosphatase.

† ··: not available. Some included studies did not provide data for 0 -5 m, 6-11 m, 12-59 m, or 0-59 m while provided data for other age groups (e.g., 0-23 m).

Location (reference)	Case definition	Specimen and test	0 –5 m		6–11 m		12–59 m		0–59 m		
			Cases (No.)	hCFR (%)	Cases (No.)	hCFR (%)	Cases (No.)	hCFR (%)	Cases (No.)	hCFR (%)	Published references (for unpublished data)
**Istanbul, Turkey (2006–2007) (Hatipoglu et al. 2011)[75]**	ALRI–Fever; ALRI	NPS; DFA	··	··	··	··	··	··	15	0	··
**Haryana, India (2009–2011) (Broor et al. 2014)[76]**	ARI	NS and TS; PCR	··	··	··	··	··	··	10	0	··
**multiple sites, Korea (1994–1998) (Kim et al. 2011) [77]**	ALRI; croup	NPA; IFA	··	··	··	··	··	··	··	··	··
**Bueno Aires, Cordoba, Santa Fe, Mar del Plata, Argentina (1993–1994) (Carballal et al. 2001) [78]**	ALRI	NPA; DFA	··	··	··	··	··	··	27	0	··
**Sousse area, Tunisia (2013–2014) (Brini et al. 2017) [79]**	ARI	NPA; PCR	··	··	··	··	··	··	44	15.9	··
**Egypt (2007–2014) (Horton et al. 2017)[80]**	ARI	NPS and OPS; PCR	··	··	··	··	151	1.3	335	3.6	··
**Jordan (2008–2010) (Horton et al. 2017) [80]**	ARI	NPS and OPS; PCR	··	··	··	··	25	0	58	1.7	··
**Oman (2008–2009) (Horton et al. 2017)[80]**	ARI	NPS and OPS; PCR	··	··	··	··	23	0	40	0	··
**Qatar (2008–2009) (Horton et al. 2017)[80]**	ARI	NPS and OPS; PCR	··	··	··	··	1	0	2	0	··
**Yemen (2010–2014) (Horton et al. 2017)[80]**	ARI	NPS and OPS; PCR	··	··	··	··	21	0	64	1.6	··
**Manhica, Mozambique (2006–2007) (O'Callaghan-Gordo et al. 2011) [56]**	ALRI	NPA; PCR	··	··	··	··	··	··	31	3.2	··
**PYNEH and QMH, Hong Kong (2003–2006) (Chiu et al. 2010) [40]**	ARI–Fever	NPA; DFA and culture	··	··	··	··	··	··	74	0	··
**Kunming, China (2009–2010) (郑文静 2011) [81]**	ARI	NPS; PCR	··	··	··	··	··	··	102	0	··
**Rio Grande do Sul, Brazil (1990-2017) (Gregianini, et al. 2019) [82]**	ARI	NPA and NPS; IFA	160	5.6	··	··	··	··	··	··	··
**Kawayan and Caibiran, Philippines (2014–2016) (Oshitani and colleagues, unpublished**	ALRI	NPS; PCR	3	0	5	0	4	0	12	0	42
**Naval, Philippines (Sep 2012–Jul 2016) (Oshitani and colleagues, unpublished)**	ALRI	NPS; PCR	22	0	21	0	30	0	73	0	42
**Muntinlupa, Philippines (Sep 2012–Feb 2015) (Oshitani and colleagues, unpublished)**	ALRI	NPS; PCR	1	100	1	0	1	0	3	33.3	42
**Ospital ng Palawan, Philippines (Aug 2012–Feb 2015) (Oshitani and colleagues, unpublished)**	ALRI	NPS; PCR	8	0	5	0	20	5	33	3	42
**Kilifi, Kenya (2007–2017) (Nokes and colleagues, unpublished)**	ALRI	NPS; PCR	84	2.4	50	2	67	4.5	201	3	43

Location (reference)	Case definition	Specimen and test	0 –5 m		6–11 m		12–59 m		0–59 m		
			Cases (No.)	hCFR (%)	Cases (No.)	hCFR (%)	Cases (No.)	hCFR (%)	Cases (No.)	hCFR (%)	Published references (for unpublished data)
**Buenos Aires, Argentina (Jun 2008–Dec 2010) (Echavarria and colleagues, unpublished)**	ARI	NPA; IFA	3	0	2	0	6	0	11	0	[45]
**multiple areas, Philippines (Jul 2000–Dec 2004) (Lucero and colleagues, unpublished)**	ALRI	Blood, NPS and NPA; Serum and culture	35	2.9	35	0	··	··	··	··	[46]
**Amman, Jordan (Mar 2010–Mar 2013) (Khuri-Bulos and colleagues, unpublished)**	ALRI	Nasal and throat swabs; PCR	45	0	31	0	··	··	··	··	[47]
**multiple areas, Bangladesh (2010–2014) (Homaira and colleagues, unpublished)**	ARI–Fever; ALRI	Nasal and throat swabs; PCR	27	0	16	0	15	0	58	0	[58]
**Matlab, Bangladesh (Jan 2012 – Dec 2013) (Deloria-Knoll and colleagues, unpublished)**	ALRI	NPS/OPS, Induced Sputum; PCR	9	0	8	0	25	0	42	0	[11]
**Basse, Gambia (2012–2013) (Deloria-Knoll and colleagues, unpublished)**	ALRI	NPS/OPS, Induced Sputum; PCR	50	2	43	2.3	39	2.6	132	2.3	[11]
**Lusaka, Zambia (Oct 2011 – Oct 2014) (Deloria-Knoll and colleagues, unpublished)**	ALRI	NPS/OPS, Induced Sputum; PCR	26	7.7	24	12.5	17	11.8	67	10.4	[11]
**Nakhon Phanom, Thailand (Jan 2012–Dec 2013) (Deloria-Knoll and colleagues, unpublished)**	ALRI	NP/OP and induced sputum; PCR	1	0	1	0	2	0	4	0	[11]
**Soweto, South Africa (Aug 2011 – Aug 2013) (Deloria-Knoll and colleagues, unpublished)**	ALRI	NPS/OPS, Induced Sputum; PCR	46	4.3	33	9.1	32	0	111	4.5	[11]
**Sa Kaeo, Thailand (Jan 2012–Dec 2013) (Deloria-Knoll and colleagues, unpublished)**	ALRI	NP/OP and induced sputum; PCR	··	··	3	0	3	0	6	0	[11]
**Dhaka, Bangladesh (Jan 2012 – Dec 2013) (Deloria-Knoll and colleagues, unpublished)**	ALRI	NPS/OPS, Induced Sputum; PCR	6	0	10	0	13	0	29	0	[11]
**Kilifi, Kenya (Aug 2011 – Nov 2011) (Deloria-Knoll and colleagues, unpublished)**	ALRI	NPS/OPS, Induced Sputum; PCR	17	5.9	20	0	35	2.9	72	2.8	[11]
**Bamako, Mali (Jan 2012 – Jan 2014) (Deloria-Knoll and colleagues, unpublished)**	ALRI	NPS/OPS, Induced Sputum; PCR	39	5.1	32	18.8	29	10.3	100	11	[11]
**Rabat, Morocco (Nov 2010–Dec 2011) (Bassat and colleagues, unpublished)**	ALRI	NPA; PCR	21	9.5	20	0	116	3.4	157	3.8	[83]

Location (reference)	Case definition	Specimen and test	0 –5 m		6–11 m		12–59 m		0–59 m		
			Cases (No.)	hCFR (%)	Cases (No.)	hCFR (%)	Cases (No.)	hCFR (%)	Cases (No.)	hCFR (%)	Published references (for unpublished data)
**Buenos Aires, Argentina (2000–2017) (Gentile and colleagues, unpublished)**	ALRI	NPA; IFA	140	2.1	134	5.2	110	3.6	384	3.6	48
**Manhiça, Mozambique (Jan 2011–Jul 2014) (Bassat and colleagues, unpublished)**	ALRI	NPA; PCR	16	0	11	0	18	0	45	0	84
**Iquique, Chile (2012–2013) (Fasce and colleagues, unpublished)**	ALRI	NPA; IF	5	0	2	0	7	0	14	0	49
**Concepcion, Chile (2012–2013) (Fasce and colleagues, unpublished)**	ALRI	NPA; IF	10	0	6	0	16	0	32	0	49
**Soweto, South Africa (Mar 1998–Oct 2005) (Madhi and colleagues, unpublished)**	ALRI	NPA; IFA	35	5.7	32	3.1	21	0	88	3.4	50
**Paarl, South Africa (Jun 2012–Dec 2017) (Zar and colleagues, unpublished)**	ALRI	NPS; PCR	13	7.7	9	0	9	0	31	3.2	25
**Klerksdorp, South Africa (2010–2015) (Cohen and colleagues, unpublished)**	ALRI	NPA; PCR	37	0	31	3.2	49	2	117	1.7	70 71
**Pietermaritzburg, South Africa (2010–2015) (Cohen and colleagues, unpublished)**	ALRI	NPA; PCR	59	1.7	42	0	60	1.7	161	1.2	70 71
**Colorado, United States of America (2010–2016) (Simões and colleagues, unpublished)**	ALRI	NW; PCR (and DFA)	129	0.8	105	1.9	484	1.2	718	1.3	85
**Berlin, Germany (Jan 2010–Dec 2014) (Rath and colleagues, unpublished)**	ALRI	NPS; PCR	52	1.9	38	0	104	0	194	0.5	86
**Taclobal City, Philippines (May 2008–Feb 2015) (Oshitani and colleagues, unpublished)**	ALRI	NPS; PCR	28	14.3	24	4.2	27	3.7	79	7.6	42
**Irbid, Jordan (Jan-Apr 2016) (Awad et al, 2020)[87]**	ARI	NPS; PCR							10	0	··

Table S13.6. Description of included studies reporting proportions of hospitalised hPIV-associated ALRI cases in children younger than five years (168 studies)*†

Location (reference)	Case definition	Specimen and test	0–5 m		6–11 m		12–59 m		0–59 m		
			Tested ALRI (No.)	Proportion of hPIV (%)	Tested ALRI (No.)	Proportion of hPIV (%)	Tested ALRI (No.)	Proportion of hPIV (%)	Tested ALRI (No.)	Proportion of hPIV (%)	Published reference (for unpublished data)
**São Paulo city, Brazil (2008–2010) (Durigon et al. 2015)[62]**	ARI	NPA; PCR	··	··	··	··	··	··	··	··	··
**Paris, France (2002–2004) (El-Hajje et al. 2008)[88]**	ALRI	NPA; IF	41	14.6	··	··	··	··	··	··	··
**Hamburg, Dresden, Freiburg, Bochum, Germany (1999–2001) (Forster et al. 2004)[52]**	ALRI; croup	NP secretion; PCR	··	··	··	··	··	··	··	··	··
**Montpellier, France (2003–2004) (Foulongne et al. 2006) [89]**	ARI	NPA; DFA and viral culture	··	··	··	··	··	··	602	1.5	··
**Buenos Aires, Argentina (CEMIC) (1998–2002) (Galiano et al. 2004) [90]**	ALRI	NPA and NPS; IFA	··	··	··	··	··	··	440	2.3	··
**Aracaju´, Salvador, Recife, and Maceio, Brazil (2012–2013) (Gurgel et al. 2016)[91]**	ALRI	NPA; PCR	··	··	··	··	··	··	··	··	··
**Sa Kaeo and Nakhon Phanom, Thailand (2005–2010) (Hasan et al. 2014)[33]**	ARI	NPS and serum specimens; PCR, serologic test and culture	397	6.3	··	··	··	··	3810	9.1	··
**Milwaukee County, USA (1996–1998) (Henrickson et al. 2004) [92]**	ALRI; croup	mainly NPS; EIA, culture, PCR	··	··	··	··	··	··	2750	12.5	··
**Reims, France (2007–2008) (Huguenin et al. 2012) [93]**	ALRI	NPA; PCR	··	··	··	··	··	··	··	··	··
**Yaounde, Cameroon (2011–2013) (Kenmoe et al. 2016) [94]**	ARI–Fever	NPS; PCR	··	··	··	··	··	··	307	6.8	··

* ARI: hospitalised acute respiratory infections. ALRI: physician diagnosed acute lower respiratory infections requiring hospital admission. ARI-Fever: hospitalised acute respiratory infections with fever. NS: nasal swab. TS: throat swab. NW: nasal wash. PCR: polymerase chain reaction. NPA: nasopharyngeal aspirate. NPS: nasopharyngeal swab. OPS: oropharyngeal swab. NPW: nasopharyngeal wash. IFA: indirect immunofluorescence assay. DFA: direct immunofluorescence assay. IF: immunofluorescence. EIA: enzyme immunoassay. ELISA: enzyme-linked immunosorbent assay. BAL: bronchoalveolar lavage. APAAP: alkaline phosphatase and monoclonal anti-alkaline phosphatase.

† ··: not available. Some included studies did not provide data for 0 -5 m, 6-11 m, 12-59 m, or 0-59 m while provided data for other age groups (e.g., 0-23 m).

Location (reference)	Case definition	Specimen and test	0–5 m		6–11 m		12–59 m		0–59 m		
			Tested ALRI (No.)	Proportion of hPIV (%)	Tested ALRI (No.)	Proportion of hPIV (%)	Tested ALRI (No.)	Proportion of hPIV (%)	Tested ALRI (No.)	Proportion of hPIV (%)	Published reference (for unpublished data)
**Seeb, Oman (2007–2008) (Khamis et al. 2012)** [95]	ARI	NPA; PCR	··	··	··	··	··	··	518	7.7	··
**Kuala Lumpur, Malaysia (1992–2008) (Khor et al. 2012)** [96]	ARI	mixed specimen; DFA and culture	3319	2.9	4241	4	2709	3.4	10269	3.5	··
**Kumasi, Ghana (2008) (Kwofie et al. 2012)** [97]	ALRI	NPS; PCR	30	0	··	··	··	··	128	3.9	··
**Shandong, China (2011–2013) (Liu et al. 2015)** [98]	ARI–Fever	TS; PCR	··	··	··	··	··	··	243	9.5	··
**Shanghai, China (2013–2015) (Lu et al. 2017)** [99]	ALRI	NPA; DFA	··	··	··	··	··	··	··	··	··
**Sioux Lookout, Ontario, Canada (2007–2012) (McCuskee et al. 2014)**[54]	ALRI	··; immunochromatography assay and culture	··	··	··	··	··	··	··	··	··
**São Paulo city, Brazil (2005–2007) (Pecchini et al. 2015)**	ARI–Fever; ARI	NP secretion; IFA	··	··	··	··	··	··	510	8	··
**Chonburi, Thailand (2013–2014) (Pratheepamornkull et al. 2015)** [100]	ALRI	NP specimen; PCR	··	··	··	··	··	··	102	2	··
**Yukon Kuskokwim Delta, USA (2005–2007) (Singleton et al. 2010)**[66]	ALRI	NP specimens; PCR	··	··	··	··	··	··	··	··	··
**Porto Alegre, Brazil (1992) (Straliotto et al. 2002)**	ARI–Fever; ARI	NP secretion; IFA	··	··	··	··	··	··	42	2.4	··
**Zhejiang, China (2001–2006) (Tang et al. 2008)** [101]	ALRI	NPA; IFA	··	··	··	··	··	··	··	··	··
**Taiwan (1997–1999) (Tsai et al. 2001)** [102]	ARI	TS and NPA; Culture	524	0	522	0.8	··	··	··	··	··
**Buenos Aires city and Greater Buenos Aires, Argentina (1998–2002) (Viegas et al. 2004)**[103]	ALRI	NPA; IFA	··	··	··	··	··	··	18561	1.6	··
**Beijing, China (2004–2012) (Wang et al. 2015)**[104]	Fever; ARI	NPA; DFA	8538	6.2	4077	10.3	··	··	21815	7.4	··
**Shenzhen, China (2012–2015) (Wang et al. 2016)**[105]	ARI–Fever	NPS; DFA	··	··	··	··	··	··	··	··	··
**Arizona, USA (2010–2014) (Wansaula et al. 2016)**[106]	ARI–Fever; ALRI	NPS; PCR	··	··	··	··	··	··	17	17.6	··

Location (reference)	Case definition	Specimen and test	0–5 m		6–11 m		12–59 m		0–59 m		
			Tested ALRI (No.)	Proportion of hPIV (%)	Tested ALRI (No.)	Proportion of hPIV (%)	Tested ALRI (No.)	Proportion of hPIV (%)	Tested ALRI (No.)	Proportion of hPIV (%)	Published reference (for unpublished data)
**NVSN sites, USA (2000–2004) (Weinberg et al. 2009)[36]**	Fever; ARI	NS and TS; PCR	1324	4.4	386	8.8	..	..	2798	6.8	..
**Beersheba, Israel (2001–2005) (Wolf et al. 2010)**	ALRI	NPW; DFA and culture	..	..	..	..	..	..	997	3.1	..
**Changsha, China (2010–2011) (Xiao et al. 2016) [107]**	ALRI	NPA; PCR	..	..	..	..	..	..	707	21.4	..
**Milan, Italy (2004–2008) (Zappa et al. 2011)[108]**	ALRI	Pharyngeal swabs; PCR	144	0	36	0	..	..	..	..	..
**Mirzapur, Bangladesh (1993–1994) [28]**	ALRI	NPA; ELISA	..	..	..	..	..	..	..	..	..
**ACH, Abha, Saudi Arabia (1997–2001) (Al-Shehri et al. 2005)[109]**	ALRI	NPA; ELISA and IFA	..	..	..	..	18	33.3	51	17.6	..
**KKUH, Riyadh, Saudi Arabia (1993–1996) (Bakir et al. 1998)[110]**	ALRI–Fever; ALRI	NPA; IFA and culture	..	..	..	..	..	..	1429	3.6	..
**Riyadh, Saudi Arabia (2005–2010) (Bukhari and Elhazmi 2013) [111]**	ALRI	NPA; DFA	342	1.5	131	0	..	..	..	..	..
**Hainan, China (2014) (Chen 2016)**	ALRI	NPA; PCR	..	..	..	..	..	..	..	..	..
**Ho Chi Minh City, Vietnam (2009–2010) (Do et al. 2016)[73]**	ARI	NPS; PCR	..	..	..	..	..	..	..	..	..
**Seoul, Korea (2011–2012) (Eem et al. 2014)[112]**	ARI	NPS; PCR	..	..	..	..	..	..	..	..	..
**Tokyo, Japan (2007–2012) (Hamada et al. 2014)[113]**	ALRI	NS; PCR	..	..	..	..	..	..	..	..	..
**Lanzhou, China (2011) (Huang et al. 2013) [114]**	ARI	TS; PCR	..	..	..	..	..	..	..	..	..
**Haryana, India (2009–2011) (Broor et al. 2014)[37]**	ARI	NS and TS; PCR	..	..	..	..	..	..	245	4.1	..
**Guangzhou, China (2009–2014) (Liao et al. 2015)[115]**	ARI	Pharyngeal swabs; PCR	..	..	..	..	..	..	..	..	..
**Beijing, China (2010–2012) (Liu et al. 2013) [116]**	ARI	TS; PCR	..	..	..	..	..	..	..	..	..
**Perth, Australia (2000–2005) (Moore et al. 2012)[117]**	ALRI	NPA; DFA, PCR and culture	..	..	..	..	..	..	5520	5.4	..

Location (reference)	Case definition	Specimen and test	0–5 m		6–11 m		12–59 m		0–59 m		
			Tested ALRI (No.)	Proportion of hPIV (%)	Tested ALRI (No.)	Proportion of hPIV (%)	Tested ALRI (No.)	Proportion of hPIV (%)	Tested ALRI (No.)	Proportion of hPIV (%)	Published reference (for unpublished data)
**San Luis Potosi´, Mexico (2002–2004) (Noyola et al. 2005)[118]**	ARI	NW; PCR	··	··	··	··	··	··	··	··	··
**Leganes, Madrid, Spain (2005–2008) (Calvo et al. 2010)[119]**	ALRI	NPA; PCR	··	··	··	··	··	··	··	··	··
**Shantou, China (2007) (Ou et al. 2009) [120]**	ALRI	NPA; PCR	··	··	··	··	··	··	345	10.7	··
**Warsaw, Poland (2008–2011) (Pancer et al. 2014)[121]**	ARI	NPS; PCR and EIA	··	··	··	··	··	··	297	6.1	··
**Chongqing, China (2014) (Peng et al. 2015) [122]**	ALRI	NPA; PCR	··	··	··	··	··	··	··	··	··
**Cordoba, Spain (2011) (Rodriguez et al. 2016)[123]**	ARI	NPA; DFA	··	··	··	··	··	··	223	5.8	··
**King George's Medical University, Lucknow, India (2011–2012) (Singh et al. 2014)[124]**	ALRI	NPA; PCR	··	··	··	··	85	0	155	0	··
**Amphoe Takhli, Thailand (1998–2001) (Siritantikorn et al. 2002)[125]**	ALRI; croup	NPA; IFA	··	··	··	··	··	··	421	5.5	··
**Rio de Janeiro, Brazil (1987–1989) (Sutmoller et al. 1995)[126]**	ALRI	NPA; IFA	··	··	··	··	··	··	241	0.8	··
**Paraguay (2009) (Vázquez et al. 2011)[127]**	ALRI	NS, pharyngeal samples, NPA and BAL; PCR	··	··	··	··	··	··	367	6	··
**Hangzhou, China (2001–2003) (Wang et al. 2005) [128]**	ALRI	NPA; DFA	··	··	··	··	··	··	··	··	··
**Kiel, Germany (1996–2000) (Weigl et al. 2005)[39]**	ALRI; croup	NPA; PCR	··	··	··	··	217	4.6	443	2.9	··
**Changsha, China (2007–2008) (Xiao et al. 2012) [129]**	ALRI	NPA; PCR	350	14.3	320	17.5	453	13.9	1123	15	··
**Beijing, China (2011–2012) (Zhang et al. 2015) [130]**	ALRI	Tracheal aspirate; PCR	··	··	··	··	··	··	··	··	··
**Dhaka, Bangladesh (2014–2015) (Bhuyan et al. 2017)[131]**	ARI	NS; PCR	··	··	··	··	43	4.7	200	11	··
**Seoul, Korea (1996–1998) (Ahn et al. 1999) [132]**	ALRI; croup	NPA; IFA	37	29.7	62	29	··	··	··	··	··

Location (reference)	Case definition	Specimen and test	0–5 m		6–11 m		12–59 m		0–59 m		
			Tested ALRI (No.)	Proportion of hPIV (%)	Tested ALRI (No.)	Proportion of hPIV (%)	Tested ALRI (No.)	Proportion of hPIV (%)	Tested ALRI (No.)	Proportion of hPIV (%)	Published reference (for unpublished data)
**Spain (2011–2013) (Cebey-López et al. 2015)[133]**	ALRI	NP specimens; PCR	..	..	..	..	..	..	..	..	..
**London, UK (2009–2012) (Cebey-López et al. 2015) [133]**	ALRI	NP specimens; PCR	..	..	..	..	..	..	..	..	..
**Nicosia, Cyprus (2010–2013) (Richter et al. 2016)[134]**	ARI	NS; PCR	..	..	..	..	..	..	..	..	..
**Egypt (2007–2014) (Horton et al. 2017)[80]**	ARI	NPS and OPS; PCR	..	..	..	..	1486	10.1	3292	10.1	..
**Jordan (2008–2010) (Horton et al. 2017) [80]**	ARI	NPS and OPS; PCR	..	..	..	..	249	9.6	578	9.2	..
**Oman (2008–2009) (Horton et al. 2017) [80]**	ARI	NPS and OPS; PCR	..	..	..	..	220	10.5	473	8.5	..
**Qatar (2008–2009) (Horton et al. 2017) [80]**	ARI	NPS and OPS; PCR	..	..	..	..	6	0	15	6.7	..
**Yemen (2010–2014) (Horton et al. 2017) [80]**	ARI	NPS and OPS; PCR	..	..	..	..	169	11.8	628	10	..
**Manhica, Mozambique (2006–2007) (O'Callaghan-Gordo et al. 2011)[56]**	ALRI	NPA; PCR	..	..	..	..	..	..	807	3.8	..
**Asembo, Kenya (2007–2010) (Feikin et al. 2013)[57]**	ALRI	NPS or OPS; PCR	..	..	..	..	..	..	350	10	..
**Shenzhen, China (2007–2010) (He et al. 2014)[135]**	ARI	NPA; PCR	595	7.9	408	9.1	812	9.1	1815	8.7	..
**Recife, Brazil (2008–2009) (Bezerra et al. 2011)[136]**	ARI	NPA; PCR	..	..	..	..	..	..	211	8.5	..
**Navajo and White Mountain Apache, USA (2009) (Bhat et al. 2013)[21]**	ALRI	NW; PCR	..	..	..	..	..	..	..	..	..
**Cape Town, South Africa (2003–2004) (Smuts 2008) [137]**	ARI	NPA, tracheal aspirate, BAL; IFA	..	..	..	..	..	..	1055	4	..
**Guangdong, China (2010–2011) (Xu et al. 2012)[138]**	ARI	TS; PCR	..	..	..	..	..	..	..	..	..
**Gipuzkoa, Spain (2004–2007) (Cilla et al. 2009)[41]**	ARI; ARI–Fever	NPA; PCR and culture	386	6	153	11.8	..	..	..	..	..
**Changsha, China (2012–2013) (彭颖 2014) [139]**	ALRI	NPA; PCR	143	48.3	159	42.1	293	34.1	595	39.7	..

Location (reference)	Case definition	Specimen and test	0–5 m		6–11 m		12–59 m		0–59 m		
			Tested ALRI (No.)	Proportion of hPIV (%)	Tested ALRI (No.)	Proportion of hPIV (%)	Tested ALRI (No.)	Proportion of hPIV (%)	Tested ALRI (No.)	Proportion of hPIV (%)	Published reference (for unpublished data)
**Shanghai, China (2003–2006) (曾玫 et al. 2008) [140]**	ARI	NPA; DFA	··	··	··	··	··	··	10240	3.8	··
**Jiangxi, China (2011–2012) (付晶晶 et al. 2013) [141]**	ALRI	NPA; DFA	··	··	··	··	··	··	··	··	··
**Wuhan, China (2012–2013) (杜帅先 et al. 2016)[142]**	ARI	NP secretions; DFA	··	··	··	··	··	··	··	··	··
**Yanting, China (2011–2012) (何杨 2015)[143]**	ARI	NPA; PCR	··	··	··	··	··	··	··	··	··
**Wuhan, China (2014) (杨泉 and 席金瓯 2016) [144]**	ARI	NPA; DFA	1342	3.3	1621	4.8	··	··	··	··	··
**Qingyuan, China (2014–2015) (梁大立 et al. 2015) [145]**	ARI	NPS; DFA	··	··	··	··	··	··	··	··	··
**Shaoxing, China (2011–2013) (章建伟 et al. 2014) [146]**	ARI	NPA; DFA	1854	6.9	672	5.2	··	··	··	··	··
**Nanjing, China (2013–2014) (胡剑 et al. 2015) [147]**	ALRI	NP secretions; DFA	240	5.8	94	9.6	··	··	··	··	··
**Zhuzhou, China (2011) (蒋最明 et al. 2013) [148]**	ARI	NPA; DFA	··	··	··	··	··	··	··	··	··
**Nanjing, China (2009–2012) (赵艳丰 et al. 2013) [149]**	ARI	NP secretions; DFA	··	··	··	··	··	··	··	··	··
**Shanghai, China (2000) (车大钿 et al. 2004) [150]**	ALRI	NPA; APAAP	··	··	··	··	··	··	1027	22.7	··
**Mianyang, China (2014–2015) (邓益斌 et al. 2016)[151]**	ARI	NPS; IFA	··	··	··	··	··	··	··	··	··
**Chenzhou, China (2013–2014) (吴琼 et al. 2017) [152]**	ARI–Fever	NS; PCR	··	··	··	··	··	··	489	17.6	··
**Wenzhou, China (2014) (张海邻 et al. 2017) [153]**	ALRI	NPA; DFA	··	··	··	··	··	··	922	15.7	··
**Wuxi, China (2014–2015) (杨俊钧 et al. 2017) [154]**	ARI	NPS; DFA	··	··	··	··	··	··	··	··	··
**Guangzhou, China (2015) (蔡勇 et al. 2017) [155]**	ARI	NPS; PCR	216	7.4	310	8.1	··	··	··	··	··
**Changsha, China (2015) (谢红军 and 李征 2017) [156]**	ARI	NPS; DFA	··	··	··	··	··	··	··	··	··

Location (reference)	Case definition	Specimen and test	0–5 m		6–11 m		12–59 m		0–59 m		
			Tested ALRI (No.)	Proportion of hPIV (%)	Tested ALRI (No.)	Proportion of hPIV (%)	Tested ALRI (No.)	Proportion of hPIV (%)	Tested ALRI (No.)	Proportion of hPIV (%)	Published reference (for unpublished data)
**Jiujiang, China (2016) (赵旦 et al. 2017) [157]**	ARI	NP secretion; DFA	2163	2.8	··	··	··	··	··	··	··
**Yangzhou, China (2013–2015) (金玉 et al. 2017) [158]**	ARI	NP secretion; DFA	567	12.7	435	11	··	··	··	··	··
**Bengbu, China (2015–2016) (阴睿媛 et al. 2017) [159]**	ARI	NP secretion; DFA	229	3.9	122	6.6	··	··	··	··	··
**Guangzhou, China (2009–2010) (颉雅莘 and 邓力) [160]**	ALRI	NPS; PCR	··	··	··	··	··	··	··	··	··
**Shanghai, China (2001–2002) (赵国昌 et al. 2003) [161]**	ALRI	nasotracheal aspiration; APAAP	233	3.4	134	0.7	··	··	··	··	··
**Nanjing, China (2006–2007) (秦铭 et al.) [162]**	ALRI	NPA; DFA	199	1.5	207	4.8	449	9.4	855	6.4	··
**Wenzhou, China (2003–2006) (曹淑彦 et al. 2007) [163]**	ARI	NP secretion; DFA	··	··	··	··	··	··	··	··	··
**Baiyin, China (2012–2013) (于德山 et al. 2017) [164]**	ALRI	NPA; PCR	20	20	93	23.7	··	··	391	18.2	··
**Guangxi, China (2013) (张海琼 and 俞小珍 2015) [165]**	ALRI	NPS; DFA	··	··	··	··	··	··	··	··	··
**Dujiangyan, China (2007-2009) (王世明 2011)[166]**	ALRI	NPA; IFA	··	··	··	··	··	··	··	··	··
**Lanzhou, China (2010–2011) (曹海燕 2013) [167]**	ARI	NPA; PCR	166	21.7	130	36.2	174	20.7	470	25.3	··
**Xi'an, China (1994–1997) (张艳敏 et al.)[168]**	ALRI	NP secretion; APAAP	65	20	52	15.4	··	··	··	··	··
**Dujiangyan, China (2007–2009) (曹淑彦 et al. 2007)[163]**	ALRI	NPA; IFA	··	··	··	··	··	··	··	··	··
**Fuzhou, China (1996–1997) (刘晖 and 陈敏 1999) [169]**	ALRI	NP secretion; APAAP	99	9.1	128	13.3	··	··	··	··	··
**Kunming, China (2005–2007) (李杨方 et al. 2008)[170]**	ALRI	NPA; IFA	··	··	··	··	··	··	··	··	··
**Hangzhou, China (2001–2003) (马晓路 et al. 2005) [171]**	ALRI	NPA; DFA	··	··	··	··	··	··	··	··	··
**Changsha, China (2013–2014) (刘沁 et al. 2015) [172]**	ALRI	NPA; PCR	138	21	142	31	262	23.7	542	24.9	··

**Location (reference)**	**Case definition**	**Specimen and test**	**0–5 m**		**6–11 m**		**12–59 m**		**0–59 m**		
			**Tested ALRI (No.)**	**Proportion of hPIV (%)**	**Tested ALRI (No.)**	**Proportion of hPIV (%)**	**Tested ALRI (No.)**	**Proportion of hPIV (%)**	**Tested ALRI (No.)**	**Proportion of hPIV (%)**	**Published reference (for unpublished data)**
**Chongqing, China (2009–2012) (卢庆彬 2013) [173]**	ARI	NPA; PCR	1028	24.7	506	26.7	739	21.4	2273	24.1	··
**Chenzhou, China (2010) (史文元 et al. 2012)[174]**	ALRI	NP secretions; PCR	··	··	··	··	··	··	··	··	··
**Kunming, China (2005–2006) (吴茜 et al. 2007)[175]**	ALRI	NPA; DFA	··	··	··	··	··	··	··	··	··
**Jinhua, China (2013–2014) (吴远桥 2015) [176]**	ARI	NPS; DFA	411	13.4	324	6.5	··	··	··	··	··
**Zhejiang, China (2006–2010) (张冰 et al. 2012) [177]**	ALRI	NPA; DFA	··	··	··	··	··	··	3932	3.9	··
**Foshan, China (2013–2014) (张巧玲 et al. 2014) [178]**	ALRI	NP specimens; DFA	424	8.5	506	12.3	··	··	1922	10.8	··
**Chengdu, China (2007) (张蕾 2008) [179]**	ALRI	NP specimens; DFA	··	··	··	··	··	··	··	··	··
**Shanghai, China (2011–2012) (张雪清 et al. 2013) [180]**	ALRI	NP secretions; DFA	··	··	··	··	··	··	··	··	··
**Shanghai, China (2016-2017) (Li et al, 2018) [181]**	ARI	NPS or sputum specimens; PCR	··	··	··	··	··	··	··	··	··
**Beijing and Shandoing, China (2012-2015) (Yu et al. 2018) [182]**	ARI	respiratory specimens (NPS, NPA, sputum, bronchoalveolar lavage); PCR	··	··	··	··	··	··	1206	17.2	··
**Wenzhou, China (2014-2016) (Wen, et al. 2019) [183]**	ALRI	NP secretion; PCR	··	··	··	··	··	··	··	··	··
**Shanghai, China (2008-2014) (Zhao, et al. 2019) [184]**	ALRI	NPA; PCR	139	8.6	68	26.5	··	··	··	··	··
**Suzhou, China (2006-2015) (任吟莹, et al. 2019) [185]**	ARI	NPA; DFA	··	··	··	··	··	··	15583	2.8	··
**Dongguan, China (2017-2018) (孙志豪, et al. 2019) [186]**	ALRI	throat swab; PCR	1051	5.4	554	6.7	··	··	··	··	··
**Shijiazhuang, China (2014-2017) (曹丽洁, et al. 2019) [187]**	ARI	NPS; Immunofluorescence assay	··	··	··	··	··	··	··	··	··
**Xiaogan, China (2017) (李正, et al. 2019) [188]**	ARI	NPS; DFA	··	··	··	··	··	··	··	··	··

Location (reference)	Case definition	Specimen and test	0–5 m		6–11 m		12–59 m		0–59 m		
			Tested ALRI (No.)	Proportion of hPIV (%)	Tested ALRI (No.)	Proportion of hPIV (%)	Tested ALRI (No.)	Proportion of hPIV (%)	Tested ALRI (No.)	Proportion of hPIV (%)	Published reference (for unpublished data)
**Huizhou, China (2017) (王春晖, et al. 2019)** [189]	ARI	NS or TS; PCR	··	··	··	··	··	··	··	··	··
**Taiwan (2010-2013) (Chi et al, 2020)**[60]	Radiologically confirmed; ALRI	NPS; PCR	··	··	··	··	··	··	705	3.5	··
**Republic of Korea (South Korea) (2010-2015) (Lee et al, 2020)**[190]	ALRI	NP sample; PCR	··	··	··	··	··	··	23242	17.1	··
**Zunyi, China (2017-2018) (Li et al, 2020)**[191]	Radiologically confirmed; ALRI	NPS, NPA, sputum; DFA	354	12.1	235	14.0	96	5.2	685	11.8	··
**Wenzhou, China (2008-2017) (Wen et al, 2020)**[192]	ALRI	NPA, sputum; DFA	23895	5.1	17556	6.8	36582	4.6	78033	5.3	··
**Kawayan and Caibiran, Philippines (2014–2016) (Oshitani and colleagues, unpublished**	ALRI	NPS; PCR	39	7.7	28	17.9	56	7.1	123	9.8	[42]
**Naval, Philippines (Sep 2012–Jul 2016) (Oshitani and colleagues, unpublished)**	ALRI	NPS; PCR	451	4.4	218	8.7	423	6.6	1092	6.1	[42]
**Muntinlupa, Philippines (Sep 2012–Feb 2015) (Oshitani and colleagues, unpublished)**	ALRI	NPS; PCR	70	1.4	59	1.7	59	1.7	188	1.6	[42]
**Ospital ng Palawan, Philippines (Aug 2012–Feb 2015) (Oshitani and colleagues, unpublished)**	ALRI	NPS; PCR	236	3	166	3	292	5.5	694	4	[42]
**Kilifi, Kenya (2007–2017) (Nokes and colleagues, unpublished)**	ALRI	NPS; PCR	1347	7.9	759	8.3	1265	7.3	3371	7.7	[43]
**Nha Trang city, Viet Nam (2007–2016) (Yoshida and colleagues, unpublished)**	ALRI	NPS; PCR	261	7.3	205	8.3	979	7.4	1445	7.5	[44]
**Buenos Aires, Argentina (Jun 2008–Dec 2010)**	ARI	NPA; IFA	12	8.3	18	5.6	43	11.6	73	9.6	[45]

Location (reference)	Case definition	Specimen and test	0–5 m		6–11 m		12–59 m		0–59 m		
			Tested ALRI (No.)	Proportion of hPIV (%)	Tested ALRI (No.)	Proportion of hPIV (%)	Tested ALRI (No.)	Proportion of hPIV (%)	Tested ALRI (No.)	Proportion of hPIV (%)	Published reference (for unpublished data)
**(Echavarria and colleagues, unpublished)**											
**multiple areas, Philippines (Jul 2000–Dec 2004) (Lucero and colleagues, unpublished)**	ALRI	Blood, NPS and NPA; Serum and culture	233	15	278	12.6	..	..	..	..	[46]
**Amman, Jordan (Mar 2010–Mar 2013) (Khuri-Bulos and colleagues, unpublished)**	ALRI	Nasal and throat swabs; PCR	..	..	..	..	..	..	..	..	[47]
**multiple areas, Bangladesh (2010–2014) (Homaira and colleagues, unpublished)**	ARI–Fever; ALRI	Nasal and throat swabs; PCR	451	6	198	8.1	182	8.2	831	7	[58]
**Matlab, Bangladesh (Jan 2012 – Dec 2013) (Deloria-Knoll and colleagues, unpublished)**	ALRI	NPS/OPS, Induced Sputum; PCR	94	9.6	74	10.8	159	15.7	327	12.8	[11]
**Basse, Gambia (2012–2013) (Deloria-Knoll and colleagues, unpublished)**	ALRI	NPS/OPS, Induced Sputum; PCR	256	19.5	138	31.2	229	17	623	21.2	[11]
**Lusaka, Zambia (Oct 2011 – Oct 2014) (Deloria-Knoll and colleagues, unpublished)**	ALRI	NPS/OPS, Induced Sputum; PCR	314	8.3	143	16.8	133	12.8	590	11.4	[11]
**Nakhon Phanom, Thailand (Jan 2012–Dec 2013) (Deloria-Knoll and colleagues, unpublished)**	ALRI	NP/OP and induced sputum; PCR	9	11.1	13	7.7	51	3.9	73	5.5	[11]
**Soweto, South Africa (Aug 2011 – Aug 2013) (Deloria-Knoll and colleagues, unpublished)**	ALRI	NPS/OPS, Induced Sputum; PCR	431	10.7	212	15.6	223	14.3	866	12.8	[11]
**Sa Kaeo, Thailand (Jan 2012–Dec 2013) (Deloria-Knoll and colleagues, unpublished)**	ALRI	NP/OP and induced sputum; PCR	7	0	11	27.3	33	9.1	51	11.8	[11]
**Dhaka, Bangladesh (Jan 2012 – Dec 2013) (Deloria-**	ALRI	NPS/OPS, Induced Sputum; PCR	42	14.3	47	21.3	109	11.9	198	14.6	[11]

Location (reference)	Case definition	Specimen and test	0–5 m		6–11 m		12–59 m		0–59 m		
			Tested ALRI (No.)	Proportion of hPIV (%)	Tested ALRI (No.)	Proportion of hPIV (%)	Tested ALRI (No.)	Proportion of hPIV (%)	Tested ALRI (No.)	Proportion of hPIV (%)	Published reference (for unpublished data)
**Knoll and colleagues, unpublished)**											
**Kilifi, Kenya (Aug 2011 – Nov 2011) (Deloria-Knoll and colleagues, unpublished)**	ALRI	NPS/OPS, Induced Sputum; PCR	185	9.2	116	17.2	265	13.2	566	12.7	[11]
**Bamako, Mali (Jan 2012 – Jan 2014) (Deloria-Knoll and colleagues, unpublished)**	ALRI	NPS/OPS, Induced Sputum; PCR	297	13.1	151	21.2	211	13.7	659	15.2	[11]
**Rabat, Morocco (Nov 2010– Dec 2011) (Bassat and colleagues, unpublished)**	ALRI	NPA; PCR	100	19	112	16.1	419	24.6	631	22.2	[83]
**Buenos Aires, Argentina (2000–2017) (Gentile and colleagues, unpublished)**	ALRI	NPA; IFA	4674	3	3634	3.7	4016	2.7	12311	3.1	[48]
**Manhiça, Mozambique (Jan 2011–Jul 2014) (Bassat and colleagues, unpublished)**	ALRI	NPA; PCR	114	14	96	11.5	203	6.9	413	9.9	[84]
**Iquique, Chile (2012–2013) (Fasce and colleagues, unpublished)**	ALRI	NPA; IF	312	3.2	148	5.4	217	6	677	4.6	[49]
**Concepcion, Chile (2012–2013) (Fasce and colleagues, unpublished)**	ALRI	NPA; IF	216	4.6	85	7.1	163	9.8	464	6.9	[49]
**Tehran, Iran (Islamic Republic of) (2008–2009) (Vahid and colleagues, unpublished)**	ARI–Fever	Throat swabs and washes; PCR	··	··	··	··	··	··	80	18.8	[193]
**Tehran, Iran (Islamic Republic of) (2017) (Vahid and colleagues, unpublished)**	ARI–Fever	NP secretions; IFA	··	··	··	··	··	··	100	26	··
**Tehran, Iran (Islamic Republic of) (Sep 2012– Sep 2013) (Vahid and colleagues, unpublished)**	ARI–Fever	Throat swabs; PCR	··	··	··	··	··	··	78	15.4	[194]

Location (reference)	Case definition	Specimen and test	0–5 m		6–11 m		12–59 m		0–59 m		
			Tested ALRI (No.)	Proportion of hPIV (%)	Tested ALRI (No.)	Proportion of hPIV (%)	Tested ALRI (No.)	Proportion of hPIV (%)	Tested ALRI (No.)	Proportion of hPIV (%)	Published reference (for unpublished data)
**Tehran, Iran (Islamic Republic of) (Jan 2003 to Jan 2004) (Vahid and colleagues, unpublished)**	ARI–Fever	NP secretions; IFA	11	18.2	30	43.3	55	18.2	96	26	[195]
**Tehran, Iran (Islamic Republic of) (Oct 1998–Oct 2000) (Vahid and colleagues, unpublished)**	ARI	NPS; Culture	··	··	··	··	111	18	200	17.5	[196]
**Soweto, South Africa (Mar 1998–Oct 2005) (Madhi and colleagues, unpublished)**	ALRI	NPA; IFA	962	3.6	605	5.3	1035	2	2602	3.4	[50]
**Kamalapur, Bangladesh (2013–2014) (Brooks and colleagues, unpublished)**	ALRI	NPW; PCR	19	5.3	16	12.5	32	0	67	4.5	[24]
**Paarl, South Africa (Jun 2012–Dec 2017) (Zar and colleagues, unpublished)**	ALRI	NPS; PCR	102	12.7	42	21.4	57	15.8	201	15.4	[25]
**Kathmandu and surrouning disctricts, Nepal (Jan 2006–Jan 2008) (Strand and colleagues, unpublished)**	ALRI	NPA; PCR	248	8.1	173	6.9	··	··	··	··	[26]
**Klerksdorp, South Africa (2010–2015) (Cohen and colleagues, unpublished)**	ALRI	NPA; PCR	504	7.3	269	11.5	486	10.1	1259	9.3	[70 71]
**Pietermaritzburg, South Africa (2010–2015) (Cohen and colleagues, unpublished)**	ALRI	NPA; PCR	883	6.7	442	9.5	746	8	2164	7.4	[70 71]
**Berlin, Germany (Jan 2010–Dec 2014) (Rath and colleagues, unpublished)**	ALRI	NPS; PCR	730	7.1	424	9	1358	7.7	2512	7.7	[86]
**Taclobal City, Philippines (May 2008–Feb 2015) (Oshitani and colleagues, unpublished)**	ALRI	NPS; PCR	816	2.9	510	3.9	1094	2.3	2420	2.9	[42]

Table S13.7. Description of included studies reporting hospital admission rates of hPIV-associated ALRI with hypoxaemia (13 studies)*†

Location (reference)	Case Definition	Denominator type	Specimen and test	0–5 m	6–11 m	11–23 m	24–59 m	0–59 m	Published references (for unpublished data)
**Kawayan and Caibiran, Philippines (2014–2016) (Oshitani and colleagues, unpublished**	ALRI	Defined population base	NPS; PCR	0	0	0	0	0	42
**Kilifi, Kenya (2007–2017) (Nokes and colleagues, unpublished)**	ALRI	Census derived estimate	NPS; PCR	2.5	1.2	0.7	0.1	0.6	43
**Nha Trang city, Viet Nam (2007–2016) (Yoshida and colleagues, unpublished)**	ALRI	Census derived estimate	NPS; PCR	··	··	0.2	0	0.1	44
**Buenos Aires, Argentina (Jun 2008–Dec 2010) (Echavarria and colleagues, unpublished)**	ARI	Defined population base	NPA; IFA	3	0	··	··	0.9	45
**Amman, Jordan (Mar 2010–Mar 2013) (Khuri-Bulos and colleagues, unpublished)**	ALRI	Census derived estimate	NS and TS; PCR	0.1	0.1	0	··	··	47
**Basse, Gambia (2012–2013) (Deloria-Knoll and colleagues, unpublished)**	ALRI	Census derived estimate	NP/OP and induced sputum; PCR	0.9	0.3	0.1	0.1	0.2	11
**Nakhon Phanom, Thailand (Jan 2012–Dec 2013) (Deloria-Knoll and colleagues, unpublished)**	ALRI	Census derived estimate	NP/OP and induced sputum; PCR	··	··	0	0	0.1	11
**Sa Kaeo, Thailand (Jan 2012–Dec 2013) (Deloria-Knoll and colleagues, unpublished)**	ALRI	Census derived estimate	NP/OP and induced sputum; PCR	··	··	0	0	0.1	11
**Buenos Aires, Argentina (2000–2017) (Gentile and colleagues, unpublished)**	ALRI	Defined population base	NPA; IFA	··	··	3.2	1	4	48
**Manhiça, Mozambique (Jan 2011–Jul 2014) (Bassat and colleagues, unpublished)**	ALRI	Defined population base	NPA; PCR	0.7	0.8	0.1	0.1	0.2	84
**Soweto, South Africa (Mar 1998–Oct 2005) (Madhi and colleagues, unpublished)**	ALRI	Defined population base	NPA; IFA	1.3	1	0.2	0	0.3	50
**Kamalapur, Bangladesh (2013–2014) (Brooks and colleagues, unpublished)**	ALRI	Defined population base	NPW; PCR	0.8	0	0	0	0.3	24
**Paarl, South Africa (Jun 2012–Dec 2017) (Zar and colleagues, unpublished)**	ALRI	Defined population base	NPS; PCR	6	0	0	0	0.8	25

* ARI: hospitalised acute respiratory infections. ALRI: physician diagnosed acute lower respiratory infections requiring hospital admission. NS: nasal swab. TS: throat swab. PCR: polymerase chain reaction. NPA: nasopharyngeal aspirate. NPS: nasopharyngeal swab. OPS: oropharyngeal swab. NPW: nasopharyngeal wash. IFA: indirect immunofluorescence assay.

† ··: not available.

Appendix 14. Meta-estimates by narrow age groups

Table S14.1 Incidence rates of hPIV-associated ALRI cases (per 1,000 children per year) in children younger than five years*

	0-27 d		1-2 m		3-5 m		6-11 m		12-23 m		24-59 m	
	**No of studies**	**Incidence rate**	**No of studies**	**Incidence rate**	**No of studies**	**Incidence rate**	**No of studies**	**Incidence rate**	**No of studies**	**Incidence rate**	**No of studies**	**Incidence rate**
**High child mortality setting**	4	1.8 (0-263)	5	9.3 (1.8-46.4)	5	62.9 (38-102.5)	6	77.9 (61.8-97.7)	7	60.3 (50-72.7)	5	14.9 (8.1-27.1)
**Lower middle income**	1	..	4	4.6 (0.9-22.2)	4	52.2 (32-84.1)	5	79.1 (59.1-105)	6	62.1 (49.9-76.9)	4	14 (6.6-29.8)

* Rates were not adjusted for missing hPIV-4.

Table S14.2 Incidence rates of hPIV-associated severe ALRI cases (per 1,000 children per year) by narrow age groups. [*]

	0-27 d		1-2 m		3-5 m		6-11 m		12-23 m		24-59 m	
	**No of studies**	**Incidence rate**	**No of studies**	**Incidence rate**	**No of studies**	**Incidence rate**	**No of studies**	**Incidence rate**	**No of studies**	**Incidence rate**	**No of studies**	**Incidence rate**
**High child mortality setting**	3	2.3 (0-265.2)	3	5.1 (0.2-118.3)	3	19.8 (2.9-123)	3	28.2 (8.2-92.2)	4	8.9 (1.6-49.2)	3	7.3 (4.1-13.1)
**Low and lower middle income**	3	2.3 (0-265.2)	3	5.1 (0.2-118.3)	3	19.8 (2.9-123)	3	28.2 (8.2-92.2)	4	8.9 (1.6-49.2)	3	7.3 (4.1-13.1)

[*] Rates were not adjusted for missing hPIV-4.

Table S14.3 Hospital admission rates of hPIV-associated ALRI (per 1,000 children per year) by narrow age groups.[*]

	0-27 d		1-2 m		3-5 m		6-11 m		12-23 m		24-59 m	
	**No of studies**	**Incidence rate**	**No of studies**	**Incidence rate**	**No of studies**	**Incidence rate**	**No of studies**	**Incidence rate**	**No of studies**	**Incidence rate**	**No of studies**	**Incidence rate**
**High child mortality setting**	8	1.5 (0.2-8.8)	10	4.8 (2.9-7.9)	10	5.7 (4-8.1)	10	4.4 (3-6.4)	11	1.3 (1.1-1.6)	10	0.3 (0.2-0.6)
**Low child mortality setting**	1	..	0	..	0	..	5	1.8 (0.8-4.1)	9	1.1 (0.5-2.3)	10	0.4 (0.2-0.9)
**Low and lower middle income**	5	2.1 (1.3-3.5)	6	4.9 (2.4-9.6)	6	6.3 (3.9-10.2)	7	3.4 (1.6-7)	8	1 (0.5-1.7)	6	0.3 (0.2-0.5)
**Upper middle income**	4	0.4 (0-31.3)	4	5.1 (2.2-11.5)	4	4.7 (3.2-6.8)	5	3.4 (1.8-6.6)	7	1.3 (0.8-2.2)	8	0.4 (0.1-1.1)
**High income**	0	..	0	..	0	..	3	3 (1.6-5.7)	5	1.5 (0.6-3.7)	6	0.3 (0.1-0.9)

[*] Rates were not adjusted for missing hPIV-4.

Table S14.4 Hospital admission rates of hPIV-associated ALRI with hypoxaemia (per 1,000 children per year) by narrow age groups. *

	0-27 d		1-2 m		3-5 m		6-11 m		12-23 m		24-59 m	
	**No of studies**	**Incidence rate**	**No of studies**	**Incidence rate**	**No of studies**	**Incidence rate**	**No of studies**	**Incidence rate**	**No of studies**	**Incidence rate**	**No of studies**	**Incidence rate**
**High child mortality setting**	6	0.5 (0-13)	7	0.6 (0.1-3)	7	1.7 (1.2-2.4)	7	0.8 (0.5-1.4)	8	0.2 (0.1-0.5)	8	0 (0-0.1)
**Low child mortality setting**	1	..	0	..	0	..	2	0.1 (0-0.2)	4	0.1 (0-2)	3	0 (0-7)
**Low and Lower middle income**	5	1 (0-178.7)	5	0.5 (0-5.3)	5	1.8 (1.2-2.6)	6	0.3 (0.1-0.9)	7	0.1 (0-0.4)	6	0 (0-0.2)
**Upper middle income**	2	0.5 (0-383.3)	2	0.8 (0.2-3.1)	2	1.6 (0.8-3.2)	3	0.9 (0.5-1.7)	5	0.1 (0-2.2)	5	0 (0-0.7)

* Rates were not adjusted for missing hPIV-4.

Table S14.5 hPIV-associated ALRI in-hospital proportion meta-estimates by narrow age groups*

	0-27 d		1-2 m		3-5 m		6-11 m		12-23 m		24-59 m	
	**No of studies**	**Proportion (%)**	**No of studies**	**Proportion (%)**	**No of studies**	**Proportion (%)**	**No of studies**	**Proportion (%)**	**No of studies**	**Proportion (%)**	**No of studies**	**Proportion (%)**
**High child mortality setting**	13	5.7 (3.8-8.5)	24	6.4 (4.8-8.3)	24	9.1 (7.2-11.5)	24	10.8 (8.5-13.7)	25	8.4 (6.3-11.1)	24	7.2 (5.2-9.8)
**Low child mortality setting**	15	1.8 (0.9-3.3)	9	3.8 (2.4-5.9)	10	5.8 (4.4-7.5)	40	9.4 (6.9-12.7)	23	9.8 (6.8-13.8)	17	7.2 (5.5-9.3)
**Low and Lower middle income**	11	5.1 (3.1-8.3)	20	6.4 (4.7-8.7)	20	8.8 (6.6-11.5)	19	10.8 (8-14.3)	21	8.3 (6-11.3)	19	7.6 (5.5-10.4)
**Upper middle income**	14	2.2 (1.1-4.5)	10	4.7 (2.8-7.8)	11	7.6 (5.5-10.4)	36	11.1 (8.6-14.4)	17	10.8 (7.2-15.8)	15	6.9 (4.7-10.1)
**High income**	3	1.7 (0.2-12.4)	3	5.8 (4.1-8.1)	3	6.6 (4.5-9.7)	9	4.4 (1.6-11.4)	10	7.9 (4.6-13)	7	7.2 (5-10.1)

* Pooled estimates using all available data on in-hospital proportions of hPIV-associated ALRI.

GATHER
Guidelines for Accurate and Transparent Health Estimates Reporting

Appendix 15. Checklist of information that should be included in new reports of global health estimates

Item #	Checklist item	Reported on page #
**Objectives and funding**		
**1**	Define the indicator(s), populations (including age, sex, and geographic entities), and time period(s) for which estimates were made.	summary; P4
**2**	List the funding sources for the work.	summary
**Data Inputs**		
For all data inputs from multiple sources that are synthesized as part of the study:		
**3**	Describe how the data were identified and how the data were accessed.	P4-5
**4**	Specify the inclusion and exclusion criteria. Identify all ad-hoc exclusions.	P5
**5**	Provide information on all included data sources and their main characteristics. For each data source used, report reference information or contact name/institution, population represented, data collection method, year(s) of data collection, sex and age range, diagnostic criteria or measurement method, and sample size, as relevant.	P4-5; appendix P17-23; 40-59
**6**	Identify and describe any categories of input data that have potentially important biases (e.g., based on characteristics listed in item 5).	Appendix P8, P30-38
For data inputs that contribute to the analysis but were not synthesized as part of the study:		
**7**	Describe and give sources for any other data inputs.	P20; P40-59
For all data inputs:		
**8**	Provide all data inputs in a file format from which data can be efficiently extracted (e.g., a spreadsheet rather than a PDF), including all relevant meta-data listed in item 5. For any data inputs that cannot be shared because of ethical or legal reasons, such as third-party ownership, provide a contact name or the name of the institution that retains the right to the data.	Data have been presented in the supplementary material. Data will be made available on Edinburgh Datashare (https://datashare.is.ed.ac.uk/) later.
**Data analysis**		
**9**	Provide a conceptual overview of the data analysis method. A diagram may be helpful.	Appendix P9.
**10**	Provide a detailed description of all steps of the analysis, including mathematical formulae. This description should cover, as relevant, data cleaning, data pre-processing, data adjustments and weighting of data sources, and mathematical or statistical model(s).	P5-7; appendix P15-23; 25-26.
**11**	Describe how candidate models were evaluated and how the final model (s) were selected.	P7; appendix p10-14, 16, 19, 23.
**12**	Provide the results of an evaluation of model performance, if done, as well as the results of any relevant sensitivity analysis.	Appendix p10-14, 16-23.
**13**	Describe methods for calculating uncertainty of the estimates. State which sources of uncertainty were, and were not, accounted for in the uncertainty analysis.	P6
**14**	State how analytic or statistical source code used to generate estimates can be accessed.	Major code used in this study will be made available upon request.
**Results and Discussion**		
**15**	Provide published estimates in a file format from which data can be efficiently extracted.	Estimates can be easily extracted in main table and supplementary table. Main tables will be provided on Edinburgh Datashare (https://datashare.is.ed.ac.uk/) later.
**16**	Report a quantitative measure of the uncertainty of the estimates (e.g. uncertainty intervals).	Uncertainty was reported for burden estimates throughout.
**17**	Interpret results in light of existing evidence. If updating a previous set of estimates, describe the reasons for changes in estimates.	Not applicable
**18**	Discuss limitations of the estimates. Include a discussion of any modelling assumptions or data limitations that affect interpretation of the estimates.	P10-11; appendix P17.

References

1. Troeger C, Blacker B, Khalil IA, et al. Estimates of the global, regional, and national morbidity, mortality, and aetiologies of lower respiratory infections in 195 countries, 1990–2016: a systematic analysis for the Global Burden of Disease Study 2016. *The Lancet Infectious Diseases* 2018; **18**(11): 1191-210.
2. McAllister DA, Liu L, Shi T, et al. Global, regional, and national estimates of pneumonia morbidity and mortality in children younger than 5 years between 2000 and 2015: a systematic analysis. *The Lancet Global Health* 2018.
3. Wang X, Li Y, O'Brien KL, et al. Global burden of respiratory infections associated with seasonal influenza in children under 5 years in 2018: a systematic review and modelling study. *The Lancet Global Health* 2020; **8**(4): e497-e510.
4. UNICEF. Pneumonia care-seeking 2016. https://data.unicef.org/resources/pneumonia-care-seeking-interactive-dashboard/ (accessed 1 Oct 2019).
5. Centers for Disease Control and Prevention, National Center for Health Statistics. Underlying Cause of Death 1999-2017 on CDC WONDER Online Database. December 2018 http://wonder.cdc.gov/ucd-icd10.html (accessed 23 November 2019).
6. Bennett A, Eisele T, Keating J, Yukich J. Global Trends in Care Seeking and Access to Diagnosis and Treatment of Childhood Illnesses. Rockville, Maryland, USA: ICF International, 2015.
7. Najnin N, Bennett CM, Luby SP. Inequalities in care-seeking for febrile illness of under-five children in urban Dhaka, Bangladesh. *Journal of health, population, and nutrition* 2011; **29**(5): 523-31.
8. Onyango D, Kikuvi G, Amukoye E, Omolo J. Risk factors of severe pneumonia among children aged 2-59 months in western Kenya: a case control study. *The Pan African medical journal* 2012; **13**: 45.
9. Ferdous F, Ahmed S, Das SK, et al. Pneumonia mortality and healthcare utilization in young children in rural Bangladesh: a prospective verbal autopsy study. *Tropical Medicine and Health* 2018; **46**(1): 17.
10. Ahmed M, Aleem MA, Roguski K, et al. Estimates of seasonal influenza-associated mortality in Bangladesh, 2010-2012. *Influenza Other Respir Viruses* 2018; **12**(1): 65-71.
11. Pneumonia Etiology Research for Child Health Study Group (PERCH). Causes of severe pneumonia requiring hospital admission in children without HIV infection from Africa and Asia: the PERCH multi-country case-control study. *Lancet (London, England)* 2019; **394**(10200): 757-79.
12. Benet T, Sanchez Picot V, Messaoudi M, et al. Microorganisms Associated With Pneumonia in Children <5 Years of Age in Developing and Emerging Countries: The GABRIEL Pneumonia Multicenter, Prospective, Case-Control Study. *Clinical infectious diseases : an official publication of the Infectious Diseases Society of America* 2017; **65**(4): 604-12.
13. Salzberg NT, Sivalogan K, Bassat Q, et al. Mortality Surveillance Methods to Identify and Characterize Deaths in Child Health and Mortality Prevention Surveillance Network Sites. *Clinical infectious diseases : an official publication of the Infectious Diseases Society of America* 2019; **69**(Supplement_4): S262-s73.
14. CHAMPS. Child Health and Mortality Prevention Surveillance. https://champshealth.org/ (accessed 6 Oct 2019).
15. Wang X, Li Y, O'Brien KL, et al. Global burden of respiratory infections associated with seasonal influenza in children under 5 years in 2018: a systematic review and modelling study. *The Lancet Global Health*.
16. WHO. Life tables by WHO region. July, 2017 2017 (accessed Sep 12 2017).
17. Sterne JA, White IR, Carlin JB, et al. Multiple imputation for missing data in epidemiological and clinical research: potential and pitfalls. *Bmj* 2009; **338**: b2393.
18. Rubin DB. Multiple imputation for nonresponse in surveys. New York: Wiley; 1987.
19. Honaker J, King G, Blackwell M. Amelia II: A Program for Missing Data. *J Stat Softw* 2011; **45**(7): 47.

20. Broor S, Parveen S, Bharaj P, et al. A prospective three-year cohort study of the epidemiology and virology of acute respiratory infections of children in rural India. *PLoS One* 2007; **2**(6): e491.
21. Bhat N, Tokarz R, Jain K, et al. A prospective study of agents associated with acute respiratory infection among young American Indian children. *Pediatr Infect Dis J* 2013; **32**(8): e324-33.
22. Krishnan A, Kumar R, Broor S, et al. Epidemiology of viral acute lower respiratory infections in a community-based cohort of rural north Indian children. *Journal of global health* 2019; **9**(1): 010433-.
23. Hansen CL, McCormick BJJ, Azam SI, et al. Substantial and sustained reduction in under-5 mortality, diarrhea, and pneumonia in Oshikhandass, Pakistan: evidence from two longitudinal cohort studies 15 years apart. *BMC Public Health* 2020; **20**(1): 759.
24. Nasreen S, Luby SP, Brooks WA, et al. Population-Based Incidence of Severe Acute Respiratory Virus Infections among Children Aged <5 Years in Rural Bangladesh, June–October 2010. *PLOS ONE* 2014; **9**(2): e89978.
25. le Roux DM, Myer L, Nicol MP, Zar HJ. Incidence and severity of childhood pneumonia in the first year of life in a South African birth cohort: the Drakenstein Child Health Study. *The Lancet Global Health*; **3**(2): e95-e103.
26. Mathisen M, Strand TA, Sharma BN, et al. RNA viruses in community-acquired childhood pneumonia in semi-urban Nepal; a cross-sectional study. *BMC Med* 2009; **7**: 35.
27. Williams JV, Harris PA, Tollefson SJ, et al. Human metapneumovirus and lower respiratory tract disease in otherwise healthy infants and children. *New England Journal of Medicine* 2004; **350**(5): 443-50.
28. The importance of viral infection in pneumonia among children under age 2 years. *HSB* 2006; **4**(4).
29. Sarna M, Lambert SB, Sloots TP, et al. Viruses causing lower respiratory symptoms in young children: findings from the ORChID birth cohort. *Thorax* 2018; **73**(10): 969-79.
30. Kusel MM, de Klerk NH, Holt PG, Kebadze T, Johnston SL, Sly PD. Role of respiratory viruses in acute upper and lower respiratory tract illness in the first year of life: a birth cohort study. *Pediatr Infect Dis J* 2006; **25**(8): 680-6.
31. Havers FP, Fry AM, Goswami D, et al. Population-based Incidence of Childhood Pneumonia Associated with Viral Infections in Bangladesh. *Pediatric Infectious Disease Journal* 2019; **38**(4): 344-50.
32. Ali A, Akhund T, Warraich GJ, et al. Respiratory Viruses Associated With Severe Pneumonia in Children Under 2 Years Old in a Rural Community in Pakistan. *Journal of Medical Virology* 2016; **88**(11): 1882-90.
33. Hasan R, Rhodes J, Thamthitiwat S, et al. Incidence and etiology of acute lower respiratory tract infections in hospitalized children younger than 5 years in rural Thailand. *Pediatric Infectious Disease Journal* 2014; **33**(2): e45-52.
34. Jain S, Williams DJ, Arnold SR, et al. Community-acquired pneumonia requiring hospitalization among U.S. children. *New England Journal of Medicine* 2015; **372**(9): 835-45.
35. Banerji A, Bell A, Mills EL, et al. Lower respiratory tract infections in Inuit infants on Baffin Island. *CMAJ* 2001; **164**(13): 1847-50.
36. Weinberg GA, Hall CB, Iwane MK, et al. Parainfluenza virus infection of young children: estimates of the population-based burden of hospitalization. *Journal of Pediatrics* 2009; **154**(5): 694-9.
37. Broor S, Dawood FS, Pandey BG, et al. Rates of respiratory virus-associated hospitalization in children aged <5 years in rural northern India. *Journal of Infection* 2014; **68**(3): 281-9.
38. Ahmed JA, Katz MA, Auko E, et al. Epidemiology of respiratory viral infections in two long-term refugee camps in Kenya, 2007-2010. *BMC Infectious Diseases* 2012; **12**: 7.

39. Weigl JA, Puppe W, Belke O, Neususs J, Bagci F, Schmitt HJ. The descriptive epidemiology of severe lower respiratory tract infections in children in Kiel, Germany. *Klinische Padiatrie* 2005; **217**(5): 259-67.
40. Chiu SS, Chan K-H, Chen H, et al. Virologically confirmed population-based burden of hospitalization caused by respiratory syncytial virus, adenovirus, and parainfluenza viruses in children in Hong Kong. *Pediatr Infect Dis J* 2010; **29**(12): 1088-92.
41. Cilla G, Onate E, Perez-Yarza EG, Montes M, Vicente D, Perez-Trallero E. Hospitalization rates for human metapneumovirus infection among 0- to 3-year-olds in Gipuzkoa (Basque Country), Spain. *Epidemiology and infection* 2009; **137**(1): 66-72.
42. Ueno F, Tamaki R, Saito M, et al. Age-specific incidence rates and risk factors for respiratory syncytial virus-associated lower respiratory tract illness in cohort children under 5 years old in the Philippines. *Influenza and Other Respiratory Viruses* 2019; **13**(4): 339-53.
43. Berkley JA, Munywoki P, Ngama M, et al. Viral etiology of severe pneumonia among Kenyan infants and children. *JAMA - Journal of the American Medical Association* 2010; **303**(20): 2051-7.
44. Yoshida L-M, Suzuki M, Thiem VD, et al. Population based cohort study for pediatric infectious diseases research in Vietnam. *Tropical medicine and health* 2014; **42**(2 Suppl): 47-58.
45. Marcone DN, Durand LO, Azziz-Baumgartner E, et al. Incidence of viral respiratory infections in a prospective cohort of outpatient and hospitalized children aged ≤5 years and its associated cost in Buenos Aires, Argentina. *BMC Infectious Diseases* 2015; **15**(1): 447.
46. Lucero MG, Nohynek H, Williams G, et al. Efficacy of an 11-valent pneumococcal conjugate vaccine against radiologically confirmed pneumonia among children less than 2 years of age in the Philippines: a randomized, double-blind, placebo-controlled trial. *Pediatr Infect Dis J* 2009; **28**(6): 455-62.
47. Halasa N, Williams J, Faouri S, et al. Natural history and epidemiology of respiratory syncytial virus infection in the Middle East: Hospital surveillance for children under age two in Jordan. *Vaccine* 2015; **33**(47): 6479-87.
48. Gentile A, Lucion MF, Juarez MdV, et al. Burden of Respiratory Syncytial Virus Disease and Mortality Risk Factors in Argentina: 18 Years of Active Surveillance in a Children's Hospital. *The Pediatric Infectious Disease Journal* 2019; **38**(6).
49. Sotomayor V, Fasce RA, Vergara N, De la Fuente F, Loayza S, Palekar R. Estimating the burden of influenza-associated hospitalizations and deaths in Chile during 2012-2014. *Influenza Other Respir Viruses* 2018; **12**(1): 138-45.
50. Madhi SA, Kuwanda L, Cutland C, Klugman KP. The Impact of a 9-Valent Pneumococcal Conjugate Vaccine on the Public Health Burden of Pneumonia in HIV-Infected and -Uninfected Children. *Clinical Infectious Diseases* 2005; **40**(10): 1511-8.
51. Cohen C, Walaza S, Moyes J, et al. Epidemiology of viral-associated acute lower respiratory tract infection among children <5 years of age in a high HIV prevalence setting, South Africa, 2009-2012. *Pediatric Infectious Disease Journal* 2015; **34**(1): 66-72.
52. Forster J, Ihorst G, Rieger CHL, et al. Prospective population-based study of viral lower respiratory tract infections in children under 3 years of age (the PRI.DE study). *European Journal of Pediatrics* 2004; **163**(12): 709-16.
53. Ji W, Zhang T, Zhang X, et al. The epidemiology of hospitalized influenza in children, a two year population-based study in the People's Republic of China. *BMC health services research* 2010; **10**: 82.
54. McCuskee S, Kirlew M, Kelly L, Fewer S, Kovesi T. Bronchiolitis and pneumonia requiring hospitalization in young first nations children in Northern Ontario, Canada. *Pediatr Infect Dis J* 2014; **33**(10): 1023-6.
55. Olabarrieta I, Gonzalez-Carrasco E, Calvo C, Pozo F, Casas I, Garcia-Garcia ML. Hospital admission due to respiratory viral infections in moderate preterm, late preterm and term infants during their first year of life. *Allergologia et Immunopathologia* 2015; **43**(5): 469-73.

56. O'Callaghan-Gordo C, Bassat Q, Morais L, et al. Etiology and epidemiology of viral pneumonia among hospitalized children in rural Mozambique: a malaria endemic area with high prevalence of human immunodeficiency virus. *Pediatr Infect Dis J* 2011; **30**(1): 39-44.
57. Feikin DR, Njenga MK, Bigogo G, et al. Viral and bacterial causes of severe acute respiratory illness among children aged less than 5 years in a high malaria prevalence area of western Kenya, 2007-2010. *Pediatr Infect Dis J* 2013; **32**(1): e14-9.
58. Homaira N, Luby SP, Hossain K, et al. Respiratory Viruses Associated Hospitalization among Children Aged <5 Years in Bangladesh: 2010-2014. *PLOS ONE* 2016; **11**(2): e0147982.
59. Mackenzie GA, Vilane A, Salaudeen R, et al. Respiratory syncytial, parainfluenza and influenza virus infection in young children with acute lower respiratory infection in rural Gambia. *Scientific reports* 2019; **9**(1): 17965.
60. Chi H, Huang YC, Liu CC, et al. Characteristics and etiology of hospitalized pediatric community-acquired pneumonia in Taiwan. *Journal of the Formosan Medical Association* 2020; **119**(10): 1490-9.
61. Saha S, Grimwood K, Lambert SB, Sarna M, Ware RS. Parainfluenza Virus Infection in an Australian Community-based Birth Cohort. *The Pediatric Infectious Disease Journal* 2020; **39**(9): e284-e7.
62. Durigon GS, Oliveira DBL, Felicio MCC, et al. Poor outcome of acute respiratory infection in young children with underlying health condition in Brazil. *International journal of infectious diseases : IJID : official publication of the International Society for Infectious Diseases* 2015; **34**: 3-7.
63. Guerrier G, Goyet S, Chheng ET, et al. Acute viral lower respiratory tract infections in Cambodian children: clinical and epidemiologic characteristics. *Pediatr Infect Dis J* 2013; **32**(1): e8-13.
64. Morgan OW, Chittaganpitch M, Clague B, et al. Hospitalization due to human parainfluenza virus–associated lower respiratory tract illness in rural Thailand. *Influenza and other respiratory viruses* 2013; **7**(3): 280-5.
65. Pecchini R, Berezin EN, Souza MC, et al. Parainfluenza virus as a cause of acute respiratory infection in hospitalized children. *Brazilian Journal of Infectious Diseases* 2015; **19**(4): 358-62.
66. Singleton RJ, Bulkow LR, Miernyk K, et al. Viral respiratory infections in hospitalized and community control children in Alaska. *Journal of Medical Virology* 2010; **82**(7): 1282-90.
67. Vega-Briceño Le, Pulgar B D, Potin S M, Ferres G M, Sánchez D I. Características clínicas y epidemiológicas de la infección por virus parainfluenza en niños hospitalizados. *Rev Chilena Infectol* 2007; **24**(5): 377-83.
68. Yeolekar LR, Damle RG, Kamat AN, Khude MR, Simha V, Pandit AN. Respiratory viruses in acute respiratory tract infections in Western India. *Indian Journal of Pediatrics* 2008; **75**(4): 341-5.
69. Benet T, Sylla M, Messaoudi M, et al. Etiology and Factors Associated with Pneumonia in Children under 5 Years of Age in Mali: A Prospective Case-Control Study. *PLoS ONE [Electronic Resource]* 2015; **10**(12): e0145447.
70. Cohen AL, Sahr PK, Treurnicht F, et al. Parainfluenza Virus Infection Among Human Immunodeficiency Virus (HIV)-Infected and HIV-Uninfected Children and Adults Hospitalized for Severe Acute Respiratory Illness in South Africa, 2009-2014. *Open forum infectious diseases* 2015; **2**(4): ofv139-ofv.
71. Cohen C, Moyes J, Tempia S, et al. Epidemiology of Acute Lower Respiratory Tract Infection in HIV-Exposed Uninfected Infants. *Pediatrics* 2016; **137**(4).
72. Do AHL, van Doorn HR, Nghiem MN, et al. Viral etiologies of acute respiratory infections among hospitalized Vietnamese children in Ho Chi Minh City, 2004–2008. *PloS one* 2011; **6**(3): e18176.
73. Do LAH, Bryant JE, Tran AT, et al. Respiratory syncytial virus and other viral infections among children under two years old in southern Vietnam 2009-2010: Clinical characteristics and disease severity. *PLoS ONE* 2016; **11 (8) (no pagination)**(e0160606).

74. García García ML, Ordobás Gabin M, Calvo Reya C, et al. Infecciones virales de vías respiratorias inferiores en lactantes hospitalizados: etiología, características clínicas y factores de riesgo. *An Esp Pediatr* 2001; **55**(2): 101-7.
75. Hatipoglu N, Somer A, Badur S, et al. Viral etiology in hospitalized children with acute lower respiratory tract infection. *Turkish Journal of Pediatrics* 2011; **53**(5): 508-16.
76. Broor S, Dawood FS, Pandey BG, et al. Rates of respiratory virus-associated hospitalization in children aged <5 years in rural northern India. *Journal of Infection* 2014; **68**(3): 281-9.
77. Kim C, Ahmed JA, Eidex RB, et al. Comparison of Nasopharyngeal and Oropharyngeal Swabs for the Diagnosis of Eight Respiratory Viruses by Real-Time Reverse Transcription-PCR Assays. *PLOS ONE* 2011; **6**(6): e21610.
78. Carballal G, Videla CM, Espinosa MA, et al. Multicentered study of viral acute lower respiratory infections in children from four cities of Argentina, 1993-1994. *J Med Virol* 2001; **64**(2): 167-74.
79. Brini I, Guerrero A, Hannachi N, et al. Epidemiology and clinical profile of pathogens responsible for the hospitalization of children in Sousse area, Tunisia. *PLOS ONE* 2017; **12**(11): e0188325.
80. Horton KC, Dueger EL, Kandeel A, et al. Viral etiology, seasonality and severity of hospitalized patients with severe acute respiratory infections in the Eastern Mediterranean Region, 2007–2014. *PLOS ONE* 2017; **12**(7): e0180954.
81. 郑文静. 昆明地区儿童呼吸道感染病原学和临床流行病学研究 [硕士]: 昆明医学院; 2011.
82. Gregianini TS, Seadi CF, Zavarize Neto LD, et al. A 28-year study of human parainfluenza in Rio Grande do Sul, Southern Brazil. *Journal of Medical Virology* 2019; **91**(8): 1423-31.
83. Jroundi I, Mahraoui C, Benmessaoud R, et al. A comparison of human metapneumovirus and respiratory syncytial virus WHO-defined severe pneumonia in Moroccan children. *Epidemiology and infection* 2016; **144**(3): 516-26.
84. Nhampossa T, Mandomando I, Acacio S, et al. Diarrheal Disease in Rural Mozambique: Burden, Risk Factors and Etiology of Diarrheal Disease among Children Aged 0–59 Months Seeking Care at Health Facilities. *PLOS ONE* 2015; **10**(5): e0119824.
85. Czaja CA, Cockburn MG, Colborn K, et al. Evaluation of rates of laboratory-confirmed influenza hospitalization in rural and urban census tracts over eight influenza seasons. *Preventive Medicine* 2020; **139**: 106184.
86. Rath B, Conrad T, Myles P, et al. Influenza and other respiratory viruses: standardizing disease severity in surveillance and clinical trials. *Expert Review of Anti-infective Therapy* 2017; **15**(6): 545-68.
87. Awad S, Khader Y, Mansi M, et al. Viral Surveillance of Children with Acute Respiratory Infection in Two Main Hospitals in Northern Jordan, Irbid, during Winter of 2016. *J Pediatr Infect Dis* 2020; **15**(1): 1-10.
88. El-Hajje M-J, Moulin F, de Suremain N, et al. La fre´quence du virus respiratoire syncytial et des autres virus respiratoires dans les hospitalisations de l’enfant Une enqueˆ te de 3 ans. *Presse Med* 2008; **37**(1 Pt 1): 37-43.
89. Foulongne V, Guyon G, Rodiere M, Segondy M. Human metapneumovirus infection in young children hospitalized with respiratory tract disease. *Pediatric Infectious Disease Journal* 2006; **25**(4): 354-9.
90. Galiano M, Videla C, Puch SS, Martínez A, Echavarría M, Carballal G. Evidence of human metapneumovirus in children in Argentina. *J Med Virol* 2004; **72**(2): 299-303.
91. Gurgel RQ, Bezerra PG, Duarte Mdo C, et al. Relative frequency, Possible Risk Factors, Viral Codetection Rates, and Seasonality of Respiratory Syncytial Virus Among Children With Lower Respiratory Tract Infection in Northeastern Brazil. *Medicine* 2016; **95**(15): e3090.
92. Henrickson KJ, Hoover S, Kehl KS, Hua W. National disease burden of respiratory viruses detected in children by polymerase chain reaction. *Pediatr Infect Dis J* 2004; **23**(1 Suppl): S11-8.

93. Huguenin A, Moutte L, Renois F, et al. Broad respiratory virus detection in infants hospitalized for bronchiolitis by use of a multiplex RT-PCR DNA microarray system. *Journal of Medical Virology* 2012; **84**(6): 979-85.
94. Kenmoe S, Tchendjou P, Vernet MA, et al. Viral etiology of severe acute respiratory infections in hospitalized children in Cameroon, 2011-2013. *Influenza and other Respiratory Viruses* 2016; **10**(5): 386-93.
95. Khamis FA, Al-Kobaisi MF, Al-Areimi WS, Al-Kindi H, Al-Zakwani I. Epidemiology of respiratory virus infections among infants and young children admitted to hospital in Oman. *Journal of Medical Virology* 2012; **84**(8): 1323-9.
96. Khor C-S, Sam I-C, Hooi P-S, Quek K-F, Chan Y-F. Epidemiology and seasonality of respiratory viral infections in hospitalized children in Kuala Lumpur, Malaysia: a retrospective study of 27 years. *BMC Pediatrics* 2012; **12**(1): 32.
97. Kwofie TB, Anane YA, Nkrumah B, Annan A, Nguah SB, Owusu M. Respiratory viruses in children hospitalized for acute lower respiratory tract infection in Ghana. *Virology Journal* 2012; **9**: 78.
98. Liu Y, Li N, Zhang S, Zhang F, Lian H, Hu R. Parainfluenza Virus 5 as Possible Cause of Severe Respiratory Disease in Calves, China. *Emerg Infect Dis* 2015; **21**(12): 2242-4.
99. Lu AZ, Shi P, Wang LB, Qian LL, Zhang XB. Diagnostic value of nasopharyngeal aspirates in children with lower respiratory tract infections. *Chinese Medical Journal* 2017; **130**(6): 647-51.
100. Pratheepamornkull T, Ratanakorn W, Samransamruajkit R, Poovorawan Y. Causative Agents of Severe Community Acquired Viral Pneumonia among Children in Eastern Thailand. *Southeast Asian Journal of Tropical Medicine & Public Health* 2015; **46**(4): 650-6.
101. Tang LF, Wang TL, Tang HF, Chen ZM. Viral pathogens of acute lower respiratory tract infection in China. *Indian Pediatr* 2008; **45**(12): 971-5.
102. Tsai HP, Kuo PH, Liu CC, Wang JR. Respiratory viral infections among pediatric inpatients and outpatients in Taiwan from 1997 to 1999. *J Clin Microbiol* 2001; **39**(1): 111-8.
103. Viegas M, Barrero PR, Maffey AF, Mistchenko AS. Respiratory viruses seasonality in children under five years of age in Buenos Aires, Argentina: a five-year analysis. *The Journal of infection* 2004; **49**(3): 222-8.
104. Wang F, Zhao LQ, Zhu RN, et al. Parainfluenza Virus Types 1, 2, and 3 in Pediatric Patients with Acute Respiratory Infections in Beijing During 2004 to 2012. *Chinese Medical Journal* 2015; **128**(20): 2726-30.
105. Wang H, Zheng Y, Deng J, et al. Prevalence of respiratory viruses among children hospitalized from respiratory infections in Shenzhen, China. *Virology Journal* 2016; **13**: 39.
106. Wansaula Z, Olsen SJ, Casal MG, et al. Surveillance for severe acute respiratory infections in Southern Arizona, 2010-2014. *Influenza and other Respiratory Viruses* 2016; **10**(3): 161-9.
107. Xiao NG, Duan ZJ, Xie ZP, et al. Human parainfluenza virus types 1-4 in hospitalized children with acute lower respiratory infections in China. *J Med Virol* 2016; **88**(12): 2085-91.
108. Zappa A, Canuti M, Frati E, et al. Co- circulation of genetically distinct human metapneumovirus and human bocavirus strains in young children with respiratory tract infections in Italy. *Journal of Medical Virology* 2011; **83**(1): 156-64.
109. Al-Shehri MA, Sadeq A, Quli K. Bronchiolitis in Abha, Southwest Saudi Arabia: viral etiology and predictors for hospital admission. *West Afr J Med* 2005; **24**(4): 299-304.
110. Bakir TM, Halawani M, Ramia S. Viral aetiology and epidemiology of acute respiratory infections in hospitalized Saudi children. *Journal of Tropical Pediatrics* 1998; **44**(2): 100-3.
111. Bukhari EE, Elhazmi MM. Viral agents causing acute lower respiratory tract infections in hospitalized children at a tertiary care center in Saudi Arabia. *Saudi Medical Journal* 2013; **34**(11): 1151-5.
112. Eem YJ, Bae EY, Lee JH, Jeong DC. Risk factors associated with respiratory virus detection in infants younger than 90 days of age. [Korean]. *Korean Journal of Pediatric Infectious Diseases* 2014; **21**(1): 22-8.

113. Hamada H, Ogura A, Hotta C, Wakui T, Ogawa T, Terai M. [Epidemiological study of respiratory viruses detected in patients under two years old who required admission because of lower respiratory disease]. *Kansenshogaku Zasshi* 2014; **88**(4): 423-9.
114. Huang G, Yu D, Mao N, et al. Viral Etiology of Acute Respiratory Infection in Gansu Province, China, 2011. *PLoS ONE* 2013; **8 (5) (no pagination)**(e64254).
115. Liao X, Hu Z, Liu W, et al. New Epidemiological and Clinical Signatures of 18 Pathogens from Respiratory Tract Infections Based on a 5-Year Study. *PLoS ONE [Electronic Resource]* 2015; **10**(9): e0138684.
116. Liu W-K, Liu Q, Chen D-H, et al. Epidemiology and clinical presentation of the four human parainfluenza virus types. *BMC infectious diseases* 2013; **13**(1): 28.
117. Moore HC, de Klerk N, Keil AD, et al. Use of data linkage to investigate the aetiology of acute lower respiratory infection hospitalisations in children. *Journal of Paediatrics & Child Health* 2012; **48**(6): 520-8.
118. Noyola DE, Alpuche-Solís AG, Herrera-Díaz A, Soria-Guerra RE, Sánchez-Alvarado J, López-Revilla R. Human metapneumovirus infections in Mexico: epidemiological and clinical characteristics. *J Med Microbiol* 2005; **54**(Pt 10): 969-74.
119. Calvo C, Pozo F, Garcia-Garcia ML, et al. Detection of new respiratory viruses in hospitalized infants with bronchiolitis: a three-year prospective study. *Acta Paediatrica* 2010; **99**(6): 883-7.
120. Ou S-Y, Lin G-Y, Wu Y, Lu X-D, Lin C-X, Zhou R-B. [Viral pathogens of acute lower respiratory tract infection in hospitalized children from East Guangdong of China]. *Zhongguo Dang Dai Er Ke Za Zhi* 2009; **11**(3): 203-6.
121. Pancer KW, Gut W, Abramczuk E, Lipka B, Litwinska B. Non-influenza viruses in acute respiratory infections among young children. High prevalence of HMPV during the H1N1V.2009 pandemic in Poland. *Przeglad Epidemiologiczny* 2014; **68**(4): 627-32.
122. Peng Y, Shu C, Fu Z, Li Q-B, Liu Z, Yan L. [Pathogen detection of 1 613 cases of hospitalized children with community acquired pneumonia]. *Zhongguo Dang Dai Er Ke Za Zhi* 2015; **17**(11): 1193-9.
123. Rodriguez PE, Adamo MP, Paglini MG, Moreno L, Camara JA, Camara A. [Monoinfection of human Metapneumovirus in Cordoba: first clinical and epidemiological research in children with respiratory infection in 2011]. *Revista de la Facultad de Ciencias Medicas de Cordoba* 2016; **73**(3): 170-5.
124. Singh AK, Jain A, Jain B, et al. Viral aetiology of acute lower respiratory tract illness in hospitalised paediatric patients of a tertiary hospital: One year prospective study. *Indian Journal of Medical Microbiology* 2014; **32**(1): 13-8.
125. Siritantikorn S, Puthavathana P, Suwanjutha S, et al. Acute viral lower respiratory infections in children in a rural community in Thailand. *J Med Assoc Thai* 2002; **85 Suppl 4**: S1167-75.
126. Sutmoller F, Ferro ZP, Asensi MD, Ferreira V, Mazzei IS, Cunha BL. Etiology of acute respiratory tract infections among children in a combined community and hospital study in Rio de Janeiro. *Clinical infectious diseases : an official publication of the Infectious Diseases Society of America* 1995; **20**(4): 854-60.
127. Vázquez C, Candia C, Figueredo S, et al. Detección de metapneumovirus humano en niños menores de 5 años hospitalizados en Paraguay. *Pediatr (Asunción)* 2011; **38**(3): 199-204.
128. Wang T-l, Chen Z-m, Tang H-f, Tang L-f, Zou C-c. [Viral etiology of pneumonia in children]. *Zhejiang Da Xue Xue Bao Yi Xue Ban* 2005; **34**(6): 566-9, 73.
129. Xiao N-G, Zhang B, Duan Z-J, et al. [Viral etiology of 1165 hospitalized children with acute lower respiratory tract infection]. *Zhongguo Dang Dai Er Ke Za Zhi* 2012; **14**(1): 28-32.
130. Zhang TG, Li AH, Lyu M, Chen M, Huang F, Wu J. Detection of respiratory viral and bacterial pathogens causing pediatric community-acquired pneumonia in Beijing using real-time PCR. *Chronic Diseases and Translational Medicine* 2015; **1**(2): 110-6.

131. Bhuyan GS, Hossain MA, Sarker SK, et al. Bacterial and viral pathogen spectra of acute respiratory infections in under-5 children in hospital settings in Dhaka city. *PLoS ONE* 2017; **12 (3) (no pagination)**(e0174488).
132. Ahn JM, Choi SY, Kim DS, Kim KH. Clinical manifestation of human metapneumovirus infection in Korean children. [Korean]. *Korean Journal of Pediatric Infectious Diseases* 2013; **20**(1): 28-35.
133. Cebey-López M, Herberg J, Pardo-Seco J, et al. Viral Co-Infections in Pediatric Patients Hospitalized with Lower Tract Acute Respiratory Infections. *PLoS One* 2015; **10**(9): e0136526-e.
134. Richter J, Panayiotou C, Tryfonos C, et al. Aetiology of Acute Respiratory Tract Infections in Hospitalised Children in Cyprus. *PLOS ONE* 2016; **11**(1): e0147041.
135. He Y, Lin G-Y, Wang Q, et al. A 3-year prospective study of the epidemiology of acute respiratory viral infections in hospitalized children in Shenzhen, China. *Influenza and Other Respiratory Viruses* 2014; **8**(4): 443-51.
136. Bezerra PGM, Britto MCA, Correia JB, et al. Viral and atypical bacterial detection in acute respiratory infection in children under five years. *PloS one* 2011; **6**(4): e18928-e.
137. Smuts H. Human coronavirus NL63 infections in infants hospitalised with acute respiratory tract infections in South Africa. *Influenza & Other Respiratory Viruses* 2008; **2**(4): 135-8.
138. Xu L, He X, Zhang D-m, et al. Surveillance and genome analysis of human bocavirus in patients with respiratory infection in Guangzhou, China. *PLoS One* 2012; **7**(9): e44876-e.
139. 彭颖. 2012-2013 年长沙地区急性下呼吸道感染住院儿童病毒谱流行学调查 [硕士]: 湖南师范大学; 2014.
140. 曾玫, 王晓红, 俞蕙, 朱启镕. 上海地区儿童急性呼吸道病毒感染的流行特征. *中华传染病杂志* 2008; **26**(9): 527-32.
141. 付晶晶, 柯江维, 李红, 黄蓉, 刘志强, 段荣. 引起儿童急性下呼吸道感染的常见病毒分析. *中华医院感染学杂志* 2013; **23**(15): 3689-91.
142. 杜帅先, 陈海婧, 马玲, 马红玲, 李辰, 胡丽华. 儿童呼吸道病毒感染现状分析. *临床血液学杂志(输血与检验)* 2016; (01).
143. 何杨. 2011 ～2012 年某院门诊及住院患儿呼吸道病毒感染特征分析. *中国妇幼保健* 2015; **30**(3): 382-4.
144. 杨泉, 席金瓯. 直接免疫荧光法检测儿童呼吸道病毒抗原的结果分析. *国际检验医学杂志* 2016; **37**(16): 2331-2,3.
145. 梁大立, 陆灶其, 徐淼玲, 朱振杰. 七种呼吸道病毒抗原检测在儿童呼吸道感染中的分析. *实用检验医师杂志* 2015; **7**(4): 216-20.
146. 章建伟, 王卓英, 钟永兴. 0 ~ 2 岁婴幼儿呼吸道病毒监测及临床特征分析. *中华全科医学* 2014; **12**(7): 1087-9.
147. 胡剑, 赵凯, 朱颋. 苏州地区儿科急性下呼吸道感染住院儿童病毒病原学回顾性研究. *黑龙江医学* 2015; (10).
148. 蒋最明, 彭俊, 顾敏, 刘佳强, 纪青. 1410 例儿童呼吸道感染病原体分析. *中国感染控制杂志* 2013; **12**(2): 129-31.
149. 赵艳丰, 雷忠英, 王琳, et al. 南京地区住院儿童副流感病毒感染监测. *临床儿科杂志* 2013; **31**(1): 52-4.
150. 车大钿, 陆权, 陆敏, 季芳, 童海燕. 2000 年上海地区儿童急性下呼吸道感染的病原学研究. *中国当代儿科杂志* 2004; **6**(2): 136-8.
151. 邓益斌, 王惠敏, 肖玉荣, 成华, 潘攀. 儿科住院患儿常见的呼吸道感染非细菌病原体检测结果分析. *重庆医学* 2016; **45**(24): 3429-31.
152. 吴琼, 陈礼娟, 黄新泉, 欧书腾, 刘子菁, 范楚平. 郴州地区 5 岁以下住院儿童严重急性呼吸道感染病毒病原学研究基金项目:郴州市科技局资助重点项目(CZ2013065);郴州市第一人民医院重点项目(N2013-005).通讯作者:范楚平. *医学理论与实践* 2017; (7): 943-5,51.

153. 张海邻, 陈小芳, 吕芳芳, et al. 多重 PCR 技术检测儿童下呼吸道感染病毒和不典型病原体的价值. *温州医科大学学报* 2017; (11): 791-5,800.
154. 杨俊钧, 胡锡池, 严子禾. 无锡地区急性呼吸道病毒感染住院儿童的病原学分析. *昆明医科大学学报* 2017; (3): 119-22.
155. 蔡勇, 陈德晖, 刘文宽, 王群, 陈晓雯, 周荣. 广州地区急性呼吸道感染住院儿童病毒病原谱. *中国医学创新* 2017; (21): 19-22.
156. 谢红军, 李征. 小儿急性呼吸道感染 3309 例病毒抗原检测及分析. *湖南师范大学学报（医学版）* 2017; (1): 52-5.
157. 赵旦, 吴文蓉, 高凯华. 2016 年九江地区儿童呼吸道病毒感染病原学分析. *实验与检验医学* 2017; (4): 562-4.
158. 金玉, 刘艳, 丁允淇, 徐晓群. 扬州地区住院儿童急性呼吸道感染病毒病原学分析. *吉林医学* 2017; (9): 1707-8.
159. 阴睿媛, 徐家丽, 刘欣, 刘猛, 顾蕊, 李亚楠. 569 例呼吸道感染住院儿童 7 种常见呼吸道病毒病原学分析. *中国微生态学杂志* 2017; (6): 684-8.
160. 颉雅苹, 邓力. 1815 例小儿下呼吸道感染病原学分析. *广州医药*; **042**(003): 24-6.
161. 赵国昌, 王晓红, 朱启镕. 上海地区儿童急性肺炎病原学和临床流行病学研究. *中国感染与化疗杂志* 2003; **3**(3).
162. 秦铭, 田曼, 夏雯, 王慧云, 史圣云, 陈倩. 儿童社区获得性肺炎的病原学研究. *临床儿科杂志*; (4): 50-3.
163. 曹淑彦, 陈小芳, 李孟荣, 蔡晓红, 李昌崇, 董琳. 急性呼吸道感染住院患儿副流感病毒的检测及分析. *临床儿科杂志* 2007; (10): 841-4.
164. 于德山, 任丽丽, 陈建华, et al. 白银市 5 岁以下急性下呼吸道感染住院儿童病原学分析. *中国病毒病杂志* 2017; **7**(05): 360-5.
165. 张海琼, 俞小珍. 3496 例下呼吸道感染住院儿童的病毒病原学分析. *现代预防医学* 2015; **42**(03): 437-9+44.
166. 王世明. 小儿肺炎病原学探讨. *医学信息（中旬刊）* 2011; **24**(9): 4262-3.
167. 曹海燕. 兰州地区 2010-2011 年呼吸道感染住院患儿病原学研究 [硕士]: 兰州大学; 2013.
168. 张艳敏, 冯玉珍, 罗树舫, 雷春莲. 小儿下呼吸道感染病毒病原学动态变化的研究. *陕西医学杂志*; (3): 4-6.
169. 刘晖, 陈敏. 福州地区肺炎患儿呼吸道分泌物多种病毒检测. *海峡预防医学杂志* 1999.
170. 李杨方, 吴茜, 倪林仙, et al. 新生儿感染性肺炎病原学检测及临床研究. *中国新生儿科杂志* 2008; **23**(3): 137-40.
171. 马晓路, 徐迎春, 郑季彦, 陈学军. 新生儿肺炎的病原及临床研究. *预防医学* 2005; **17**(1): 6-8.
172. 刘沁, 张兵, 谢志萍, et al. 长沙地区急性下呼吸道感染住院儿童的病毒病原学分析. *湖南师范大学学报（医学版）* 2015; (1): 26-31.
173. 卢庆彬. 儿童急性呼吸道感染病毒流行特征与基因特征研究; 2013.
174. 史文元, 祝伟宏, 何志刚, 徐桂珍, 李莉萍. 病毒性肺炎患儿的病原学特点分析. *医学信息* 2012; **25**(3): 93.
175. 吴茜, 倪林仙, 李杨芳, et al. 急性下呼吸道感染患儿病毒病原学分析. *中国实用儿科杂志* 2007; **22**(12): 938-9.
176. 吴远桥. 儿童急性呼吸道病毒感染 1 200 例的抗原检测及分析. *中国儿童保健杂志* 2015; **23**(11): 1216-8.
177. 张冰, 王晓, 张微, 陈旭央. 儿童急性下呼吸道病毒感染的临床流行特征. *浙江医学* 2012; **34**(4): 250-2,5,后插 1.
178. 张巧玲, 钟斌才, 唐永梅. 三水地区急性呼吸道感染儿童的常见病毒谱分析. *检验医学与临床* 2014; (14).

179. 张蕾. 儿童下呼吸道感染的病毒病原检测分析 [硕士]: 泸州医学院

西南医科大学; 2008.
180. 张雪清, 胡骏, 宁小晓, 高淑芳, 王蕾. 2425 例小儿呼吸道感染 7 种常见病毒检出情况分析. *检验医学* 2013; **28**(7): 602-5.
181. Li J, Tao Y, Tang M, et al. Rapid detection of respiratory organisms with the FilmArray respiratory panel in a large children's hospital in China. *BMC Infectious Diseases* 2018; **18 (1) (no pagination)**(510).
182. Yu J, Xie Z, Zhang T, et al. Comparison of the prevalence of respiratory viruses in patients with acute respiratory infections at different hospital settings in North China, 2012-2015. *BMC Infectious Diseases* 2018; **18**(1): 72.
183. Wen S, Lv F, Chen X, et al. Application of a nucleic acid-based multiplex kit to identify viral and atypical bacterial aetiology of lower respiratory tract infection in hospitalized children. *J Med Microbiol* 2019; **68**(8): 1211-8.
184. Zhao Y, Lu R, Shen J, Xie Z, Liu G, Tan W. Comparison of viral and epidemiological profiles of hospitalized children with severe acute respiratory infection in Beijing and Shanghai, China. *BMC Infectious Diseases* 2019; **19**(1): 729.
185. 任吟莹, 顾文婧, 张新星, et al. 苏州地区儿童急性呼吸道感染病毒病原回顾性分析. *中华实用儿科临床杂志* 2019; **34**(4): 254-9.
186. 孙志豪, 张荣华, 钟超珍. 东莞市 2017-2018 年儿童下呼吸道感染病例监测结果. *分子诊断与治疗杂志* 2019; **11**(4): 329-37.
187. 曹丽洁, 帅金凤, 刘建华, et al. 2014-2017 年石家庄地区住院儿童急性呼吸道感染病毒病原学分析. *中华实验和临床病毒学杂志* 2019; **33**(4): 400-4.
188. 李正, 李萍, 袁文娟, 邱燕, 吴皖, 陈杰. 孝感市住院儿童呼吸道病毒感染病原学分析. *中国病毒病杂志* 2019; **9**(2): 154-7.
189. 王春晖, 廖惠贞, 李旭艳, 曹碧红. 不同年龄段儿童急性呼吸道病原体感染情况分析. *医学检验与临床* 2019; **30**(3): 16-8.
190. Lee E, Kim CH, Lee YJ, et al. Annual and seasonal patterns in etiologies of pediatric community-acquired pneumonia due to respiratory viruses and Mycoplasma pneumoniae requiring hospitalization in South Korea. *BMC Infectious Diseases* 2020; **20**(1): 132.
191. Li X, Zhang W, Yao S, et al. Prevalence and clinical significance of common respiratory pathogens in the upper respiratory tract of children with community-acquired pneumonia in Zunyi, China. *Pediatric Pulmonology* 2020; **55**(9): 2437-43.
192. Wen S, Yu M, Zheng G, et al. Changes in the etiology of viral lower respiratory tract infections in hospitalized children in Wenzhou, China: 2008-2017. *J Med Virol* 2020; **92**(8): 982-7.
193. Shatizadeh S, Yavarian J, Rezaie F, Mahmoodi M, Naseri M, Mokhtari Azad T. Epidemiological and clinical evaluation of children with respiratory virus infections. *Med J Islam Repub Iran* 2014; **28**: 102-.
194. Sultani M, Mokhtari Azad T, Eshragian M, et al. Multiplex SYBR Green Real-Time PCR Assay for Detection of Respiratory Viruses. *Jundishapur journal of microbiology* 2015; **8**(8): e19041-e.
195. Mamishi S, Kalantari N, Hashemi F. B, Khotaie G, Siadati S.A. Immunofluorescence method in parainfiuenza acute respiratory infection. *Tehran University Medical Journal* 2008; **65**(10): 45-9.
196. Rahbarimanesh AA, Karimi S, Gilani M. RELATIVE FREQUENCY OF PARAINFLUENZA INFECTION IN PATIENTS WITH RESPIRATORY INFECTIONS. *Acta medica Iranica* 2004; **42**: 281-4.
